# Novel Benzylidene Thiazolidinedione Derivatives as Partial PPARγ Agonists and their Antidiabetic Effects on Type 2 Diabetes

**DOI:** 10.1038/s41598-017-14776-0

**Published:** 2017-10-31

**Authors:** Sabina Yasmin, Fabio Capone, Antonio Laghezza, Fabrizio Dal Piaz, Fulvio Loiodice, Viswanathan Vijayan, Velmurugan Devadasan, Susanta K. Mondal, Özlem Atlı, Merve Baysal, Ashok K. Pattnaik, Venkatesan Jayaprakash, Antonio Lavecchia

**Affiliations:** 1 Department of Pharmaceutical Sciences & Technology, Birla Institute of Technology, Mesra, Ranchi, Jharkhand 835 215 India; 2 Department of Pharmacy, “Drug Discovery” Laboratory, University of Napoli “Federico II”, Via D. Montesano, 49, 80131 Napoli, Italy; 3 Department of Pharmacy & Drug Sciences, University of Bari “Aldo Moro”, via Orabona 4, 70125 Bari, Italy; 4 Department of Pharmacy, University of Salerno, Via Giovanni Paolo II, 132, 84084 Fisciano, Italy; 5 Centre of Advanced Study in Crystallography and Biophysics, University of Madras, Maraimalai (Guindy) Campus, 600 025 Chennai, India; 6 TCG Lifesciences Ltd, Block-EP&GP, BIPL, Tower-B, Saltlake, Sector-V, Kolkata, 700091 West Bengal India; 7 Department of Pharmaceutical Toxicology, Faculty of Pharmacy, Anadolu University, Yunus Emre Kampüsü, 26470 Eskişehir, Turkey

## Abstract

Peroxisome proliferator-activated receptor γ (PPARγ) has received significant attention as a key regulator of glucose and lipid homeostasis. In this study, we synthesized and tested a library of novel 5-benzylidene-thiazolidin-2,4-dione (BTZD) derivatives bearing a substituent on nitrogen of TZD nucleus (compounds **1a**-**1k**, **2i**-**10i**, **3a**, **6a**, and **8a**-**10a**). Three compounds (**1a**, **1i**, and **3a**) exhibited selectivity towards PPARγ and were found to be weak to moderate partial agonists. Surface Plasmon Resonance (SPR) results demonstrated binding affinity of **1a**, **1i** and **3a** towards PPARγ. Furthermore, docking experiments revealed that BTZDs interact with PPARγ through a distinct binding mode, forming primarily hydrophobic contacts with the ligand-binding pocket (LBD) without direct H-bonding interactions to key residues in H12 that are characteristic of full agonists. In addition, **1a**, **1i** and **3a** significantly improved hyperglycemia and hyperlipidaemia in streptozotocin-nicotinamide (STZ-NA)-induced diabetic rats at a dose of 36 mg/kg/day administered orally for 15 days. Histopathological investigations revealed that microscopic architecture of pancreatic and hepatic cells improved in BTZDs-treated diabetic rats. These findings suggested that **1a**, **1i** and **3a** are very promising pharmacological agents by selectively targeting PPARγ for further development in the clinical treatment of type 2 diabetes mellitus.

## Introduction

Type 2 diabetes mellitus (T2DM), formerly referred to as non-insulin dependent diabetes mellitus (NIDDM), has become a major public health issue that affects more than 285 million people worldwide^1^. It is estimated that by 2030 around 552 million people will have diabetes^2^. T2DM is a complex heterogeneous group of metabolic disorders resulting in high blood glucose due to impaired insulin secretion or insulin resistance. The major factors contributing to the development of abnormal insulin secretion or insulin resistance include genetic defects, aging, viral infection (Cytomegalovirus, Coxsackie B4 virus), environmental factors, sedentary lifestyle combined with high calorie intake^3^. T2DM leads to further long term complications such as neuropathy, nephropathy, cataracts and accelerated atherosclerosis leading to increased risk of myocardial infarction^4–6^. In fact, the prevention and control of associated complications are a serious and challenging therapeutic problem, as these factors represent the leading causes of mortality and morbidity for diabetic patients^7^.

Peroxisome Proliferator-Activated Receptors (PPARs) represent a group of transcription factors that, upon binding of a ligand, induce the expression of genes involved in the regulation of glucose homeostasis and lipid metabolism and for this reason they have been considered suitable targets for the treatment of metabolic disorders^8^. In humans, three different subtypes of these nuclear receptors have been identified: PPARα (NR1C1), PPARβ (also known as PPARδ) (NR1C2), and PPARγ (NR1C3)^9^. PPARα, that is the target of hypolipidemic drugs fibrates, is highly expressed in brown adipose tissue, liver, kidney, heart and skeletal muscle and activates a genetic program leading to β-oxidation of fatty acids^10,11^. PPARβ/δ is localized ubiquitously but its function remains as the least understood amongst PPAR subtypes although numerous studies have highlighted the physiological importance of PPARβ/δ in the regulation of fatty acid catabolism and energy homeostasis^12,13^. PPARγ is expressed in three different forms PPARγ1, PPARγ2 and PPARγ3^14^. PPARγ1 is expressed in heart, kidney, pancreas, colon, spleen; PPARγ2 is expressed in adipose tissue and PPARγ3 is predominantly found in white adipose tissue, macrophages and large intestine^15^. PPARγ acts as a key factor in various metabolic processes and plays an important role in the regulation of insulin tissue sensitivity and in the management of glucose and lipid uptake and storage^16^. Being expressed in many components of the vascular and immune system, PPARγ also exerts anti-inflammatory and anti-atherosclerotic actions^17–19^. This nuclear receptor is the target of thiazolidinedione class of insulin sensitizing drugs, used in T2DM^20^. The antihyperglycemic properties of thiazolidinediones (TZDs) came into existence by description of ciglitazone in the early 1980s and were followed by a few more glitazones in subsequent years (Fig. S1)^21–24^. Due to the associated toxicity^25–27^, some of these drugs as well as other PPARγ full agonists have been abandoned during clinical trials or withdrawn from the market^28^. Nowadays, therefore, new drugs acting as partial agonists, even weak or moderate, or selective modulators of PPARγ (SPPARγM) are being developed with the goal of retaining the beneficial effects while reducing the adverse effects^29–34^. In the past, very few 5-benzylidene-thiazolidin-2,4-dione (BTZD) derivatives have been explored for their glucose lowering and selective PPARγ modulator activity^35–39^.

In search for less toxic derivatives of TZDs, in the present paper we synthesized a library of novel BTZD derivatives bearing a substituent on the nitrogen atom of TZD nucleus (compounds **1a**-**1k**, **2i**-**10i**, **3a**, **6a**, and **8a**-**10a**) and tested them for their ability to bind and activate PPARγ by SPR experiments and transactivation assays, respectively. Among these derivatives, we identified three novel BTZD derivatives as PPARγ partial agonists (**1a**, **1i**, and **3a**). In addition, we demonstrated by means of docking experiments that BTZDs utilize a binding mode distinct from other reported PPARγ ligands, without making direct contact with any residues of helix 12. Moreover, we investigated the antidiabetic effects of **1a**, **1i**, and **3a** in streptozotocin-nicotinamide (STZ-NA)-induced diabetic rats, which proved that BTZDs improved metabolic parameters and lipid profile. These compounds had also favourable effect to inhibit the histopathological changes of the pancreas and kidney in STZ-NA-induced diabetic rats. Finally, we studied the oral bioavailability of **1a**, **1i**, and **3a** in terms of Caco-2 and MDCK-II permeability (P_app,A–B_) and the P-gp efflux ratio (P_app,B–A_/P_app,A–B_). The *in vitro* intrinsic half-life (T_1/2,int_) and apparent intrinsic clearance (CL_int,app_) was also measured after incubation with human liver microsomes at 37.5 °C. In conclusion, BTZD derivatives may represent novel antidiabetics agents with fewer side effects than PPARγ full agonists.

## Results

### Chemistry

**Figure 1:**
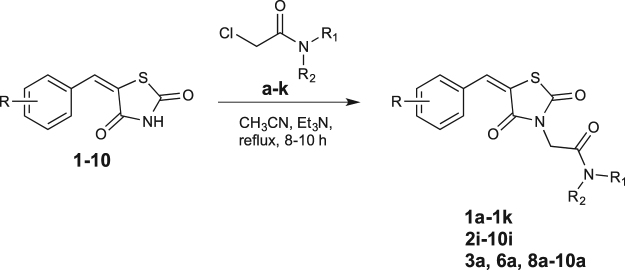
Synthetic route for novel BTZD derviatives.

BTZDs (**1–10**) were prepared by Knoevenagel condensation of thiazolidinedione with appropriate benzaldehydes in the presence of piperidine as catalyst as previously reported^40,41^. 2-Chloroacetamide derivatives (**a**-**k**) were prepared by the reaction of 2-chloroacetylchloride with appropriate amines in the presence of triethylamine in chloroform^42,43^ (Fig. 1). Coupling of BTZDs with 2-chloroacetamides in the presence of triethylamine in acetonitrile provided final compounds. Known intermediates were confirmed by comparing their melting point with the literature value and final compounds were characterized by ^1^H-NMR, ^13^C-NMR and ESI-MS data.

**Figure 2:**
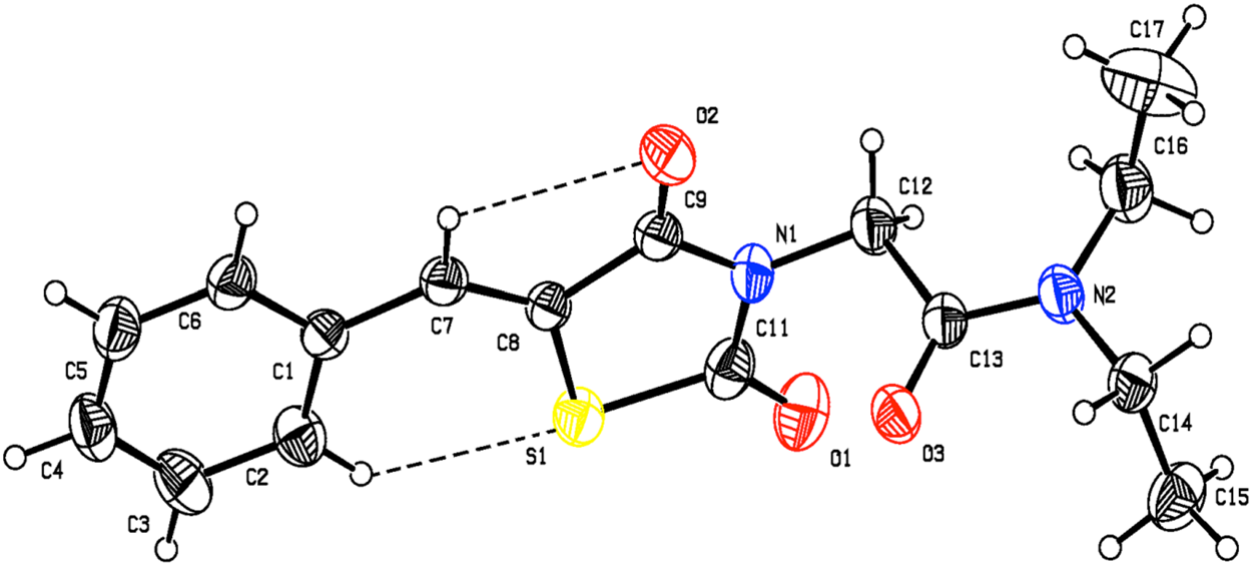
Molecular structure of **1i** showing the atom labelling and displacement ellipsoids drawn at 30% probability level.

The structure of compound **1i** was confirmed by X-ray crystallography (Fig. 2 and Table S1 of Supplementary Material). The compound crystallizes in the monoclinic P2_1_/c space group with four molecules in the unit cell (a = 7.1761(4) Å, b = 26.1554(11) Å, c = 9.0667(4) Å, α = 90°, β = 112.127(3)°, γ = 90° and Z = 4). In the crystal structure, the TZD ring adopts a planar conformation with a maximum deviation of 0.0182(3) Å for C9. The mean planes of the thiazolidine and phenyl rings are inclined to one another by 5.74(7)°. The TZD and phenyl rings lie in plane, as evidenced from the torsion angles C6-C1-C7-C8 = 175.6° and C1-C7-C8-C9 = −178.5°. The oxygen atom (O1) is oriented *syn-periplanar* to C12 [C12-N1-C11-O1 = 5.1°] and *anti-periplanar* to C9 [C9-N1-C11-O1 = 179.7°]. The oxygen atom (O2) is oriented *syn-periplanar* to C7 [C7-C8-C9-O2 = −2.0°] and *anti-periplanar* to S1 [S1-C8-C9-O2 = 178.7°]. The carbonyl group (C13/O3) is oriented *syn-periplanar* to C14 [C14-N2-C13-O3 = −0.2°] and *anti-periplanar* to C16 [C16-N2-C13-O3 = −174.5°]. The 2-aminoacetamide moiety is in extended conformation, which can be seen from the torsion angle value of N1-C12-C13-N2 = −175.3°. Finally, the two torsion angles of the diethylamine group are C13-N2-C16-C17 = −104.9° and C13-N2-C14-C15 = −84.0°, respectively (Table S2 of Supplementary Material).

As shown in Fig. 2, molecule **1i** has C-H···S and C-H···O intramolecular H-bonds forming a S(6) and S(5) ring motif. In addition, van der Waals forces also control the molecular packing, as shown in Fig. S2 of Supplementary Material. In the crystal, the molecules are stacked in layers, held together by π-π interactions, with Cg1 and Cg2 distance of 3.7435(13) Å (symmetry code Cg1 = x, y, z; Cg2 = 1 − x, −y, 2 − z.) between the centroids of two adjacent thiazolidine rings, as shown in Fig. S3 of Supplementary Material.

### Transactivation induced by compounds 1a, 1i, and 3a as selective PPARγ partial agonists

**Figure 3:**
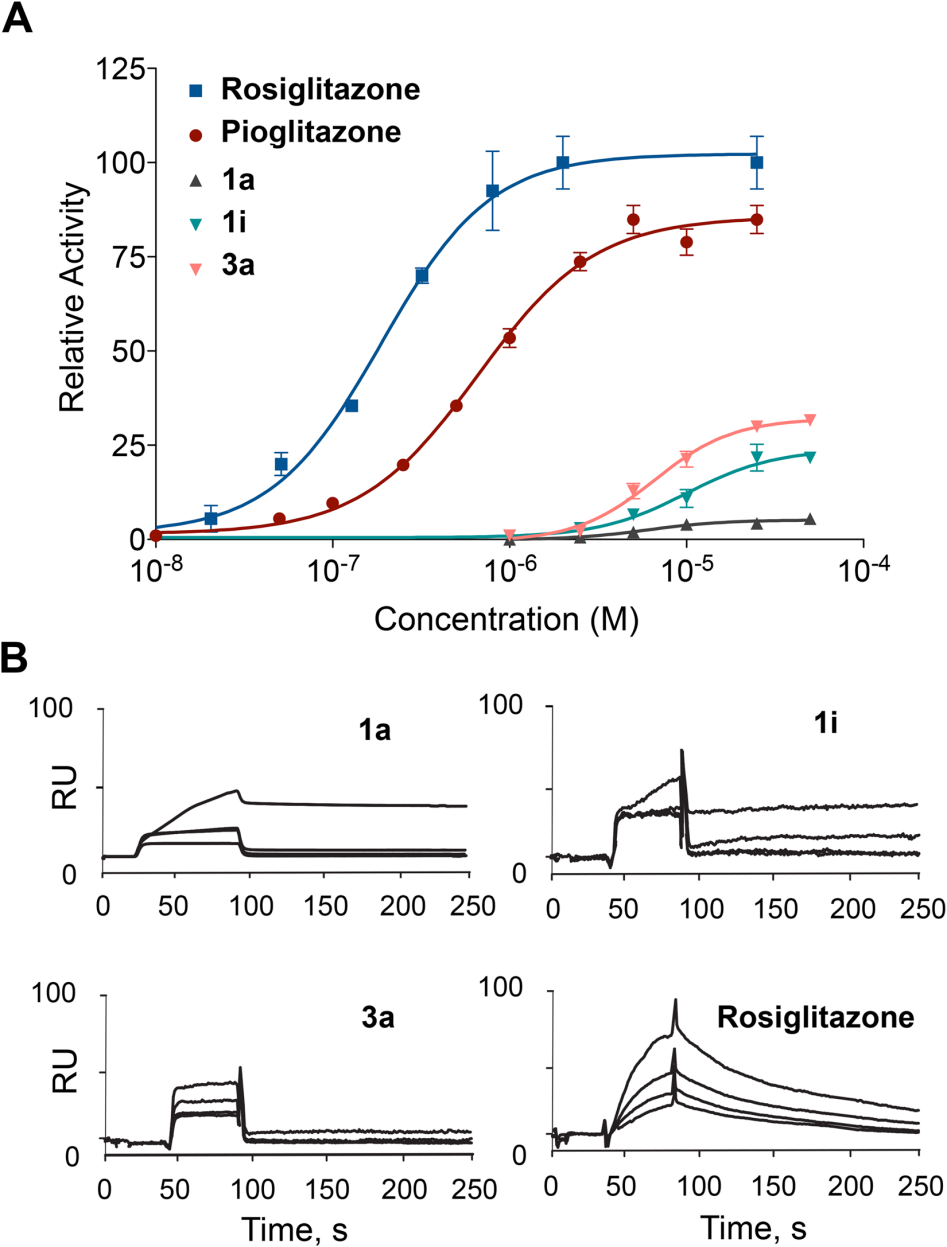
Characterization of **1a**, **1i**, and **3a** binding to PPARγ. (**A**) Concentration-response curves of Rosiglitazone, Pioglitazone, **1a**, **1i**, and **3a** in HepG2 cells co-transfected with pGal5TKpGL3 reporter and pGal4-hPPARγ-LBD. (**B**) SPR sensorgrams achieved injecting different concentrations (from 0.5 to 10 µM) of compounds **1a**, **1i**, and **3a** on immobilized PPARγ. The positive control Rosiglitazone was injected at concentrations from 0.1 to 100 nM.

A first batch of eleven compounds (**1a**-**1k**, Table 1) bearing an unsubstituted benzylidene moiety and different substituents on the acetamide nitrogen of TZD was evaluated for agonist activity on the human PPARα (hPPARα), PPARγ (hPPARγ), and PPARβ/δ (hPPARβ/δ) subtypes. For this purpose, GAL-4 PPAR chimeric receptors were expressed in transiently transfected HepG2 cells according to a previously reported procedure^44,45^. At first, the activity of these compounds was evaluated at two concentrations (5 μM and 25 μM) and compared with that of the corresponding reference agonists (Wy-14,643 for PPARα, Rosiglitazone for PPARγ and L-165,041 for PPARβ/δ) whose maximum induction was defined as 100%. Only **1a** and **1i** showed a weak to moderate selective activity towards PPARγ and, therefore, for these compounds a concentration-response curve was carried out. As displayed in Fig. 3A, their EC_50_ values were 7.0 ± 1.3 μM and 9.6 ± 0.8 μM with E_max_ 5.3 ± 0.3% and 23 ± 5%, respectively. In this figure, the concentration-response of Pioglitazone is also reported showing EC_50_ = 0.65 ± 0.01 μM and E_max_ = 80 ± 2%. Although weak or moderate, the PPARγ activity of **1a** e **1i** is not to be underestimated in that the well-known metaglidasen, having EC_50_ and E_max_ around 10 μM and 10% respectively, is still investigated as a useful agent for the treatment of T2DM and hyperglycemia, as demonstrated by some recent patents^46,47^. These results evidence the importance of flexible alkyl chains on the acetamide nitrogen atom of TZD moiety; in fact, all other derivatives present a rigid aliphatic or aromatic cycle that is probably unable to adapt its own conformation for accommodating into the PPARγ binding site. At this stage, with the aim to evaluate the influence of benzylidene moiety on PPAR activity, we introduced different substituents on the aromatic ring of **1a** and **1i** by preparing five analogues of the former (**3a**, **6a**, **8a**-**10a**) and nine analogues of the latter (**2i**-**10i**). These compounds (Table 2) were tested, again, at two concentrations (5 μM and 25 μM) towards all three PPAR subtypes. Only **3a**, bearing a chlorine atom in the para position of benzylidene, exhibited a selective activity towards PPARγ and its concentration-response curve (Fig. 3A) provided EC_50_ = 5.9 ± 0.6 μM and E_max_ = 31.6 ± 1.0% values.

**Table 1:** Chemical structure of compounds 1a-1k.


Code	R_1_	R_2_
**1a**	−H	−(CH_2_)_3_CH_3_
**1b**	−H	−*cyc*−C_3_H_5_
**1c**	−H	−*cyc*−C_4_H_7_
**1d**	−H	−*cyc*−C_5_H_9_
**1e**	−H	−*cyc*−C_6_H_11_
**1f**	−H	−C_6_H_5_
**1g**	−H	−CH_2_−C_6_H_5_
**1h**	−H	−(CH_2_)_2_−C_6_H_5_
**1i**	−C_2_H_5_	−C_2_H_5_
**1j**	−(CH_2_)_5_−	
**1k**	−(CH_2_)_2_−O−(CH_2_)_2_−

**Table 2:** Chemical structure of compounds 3a, 6a, 8a-10a and 2i-10i.


Code	R	Code	R
**3a**	4−Cl	**4i**	4−F
**6a**	4−OH	**5i**	3−OH
**8a**	4−OCH_3_	**6i**	4−OH
**9a**	4−CH_3_	**7i**	3−OCH_3_
**10a**	*thiophen-2-yl	**8i**	4−OCH_3_
**2i**	3−Cl	**9i**	4−CH_3_
**3i**	4−Cl	**10i**	*thiophen-2-yl

*thiophene ring replaces substituted phenyl ring.

The ability of BTZDs **1a**, **1i** and **3a** to interact with PPARγ was assessed by SPR-based Biacore binding assays, using Rosiglitazone as a positive control, as reported elsewhere^48^. Briefly, different concentrations (from 0.5 to 10 µM) of each compound were injected on a sensor chip, covalently modified by PPARγ. The resulting sensorgrams (Fig. 3B) showed that all the three compounds have some affinity towards PPARγ, as demonstrated by the measured K_D_ of 38.3 ± 1.8 µM, 35.7 ± 2.6 µM and 32.0 ± 1.5 µM, respectively. However, these K_D_ values are significantly lower than that measured for Rosiglitazone (320.5 ± 15.2 nM).

### BTZDs 1a, 1i and 3a recognize PPARγ in a unique binding mode

To attempt to understand the molecular mechanism underlying the interaction of **1a**, **1i** and **3a** with PPARγ and give an explanation to the significant increase of efficacy for **3a** compared to **1a**, we undertook docking studies into the PPARγ ligand-binding domain (LBD) using the GOLD Suite docking package^49^ in combination with the ChemPLP^50^ scoring function (rescoring with ChemScore)^51^. We selected the GQ-16/PPARγ crystal structure (PDB ID: 3T03)^32^ as a template for docking experiments, mainly based on the similarity of the structure features between our candidate compounds and GQ-16, both containing an BTZD portion. Analysis of the GQ-16/PPARγ complex revealed that the ligand is H-bonded via four water molecules to the protein: via W200 to Y327 (H5) and via W210, W211, and W61 to S342 (β-sheet), L340 (β-sheet), and R288 (H3). Accordingly, the four intervening water molecules were included in the docking experiments. Alternative conformations of F282 (H3), R288 (H3), F363 (H7) and Y473 (H12) side chains have been previously observed in PPARγ crystal structures complexed with several ligands, highlighting the innate plasticity of the PPARγ LBD^52–54^. Accordingly, the R288 side chain was allowed to move during the docking experiments. Validation test runs demonstrated that GOLD could reproduce the crystal bound conformation of GQ-16 and provide high alignment accuracy with low RMSD (0.74 Å, CHEMscore fitness = 33.8, CHEMscore ΔG^bind^ = −39.8 kJ/mol).

**Figure 4:**
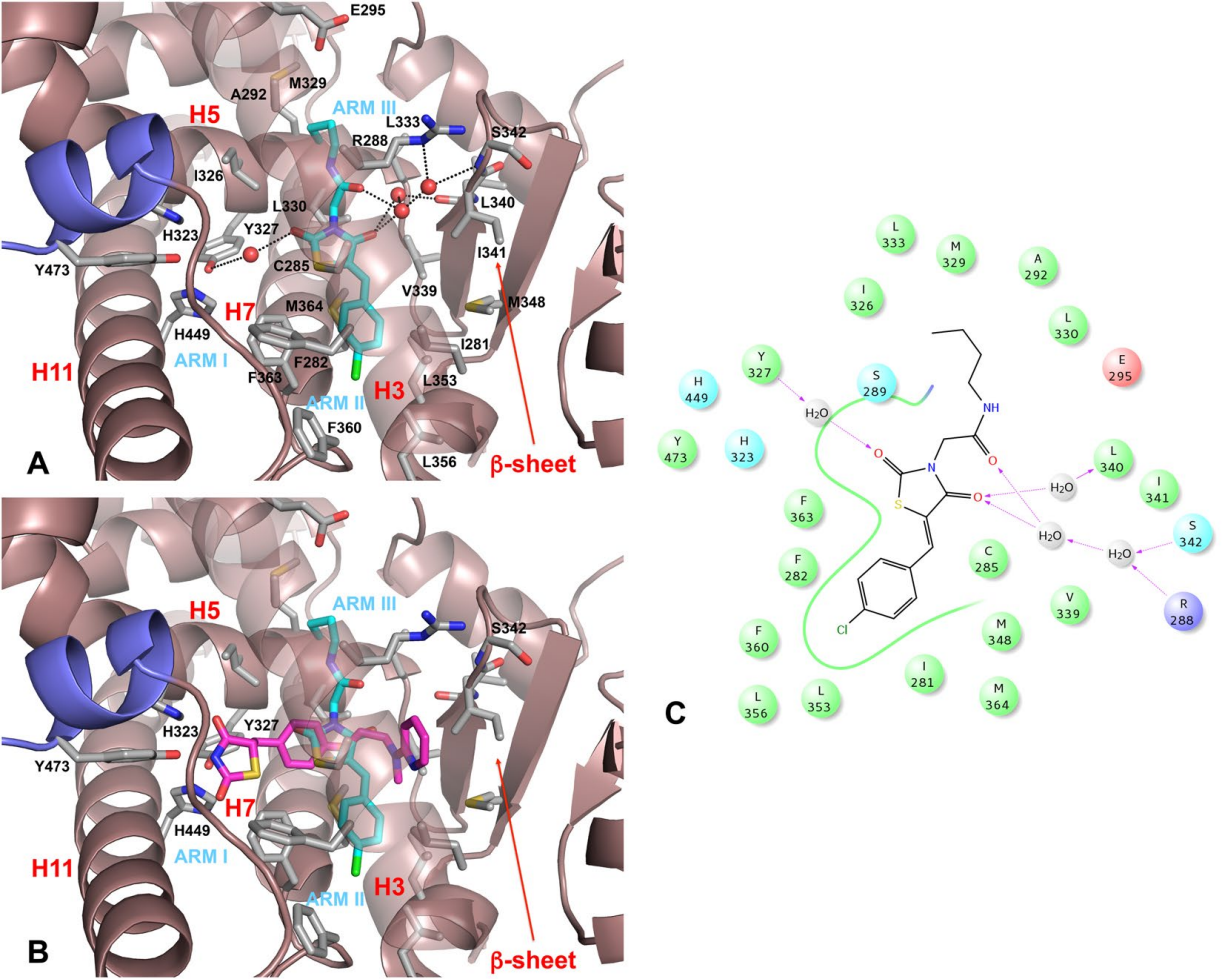
In silico docking of compound **3a** into the PPARγ binding pocket. (**A**) Binding mode of compound **3a** (partial agonist, cyan sticks) into the PPARγ binding site represented as a dirtyviolet ribbon model. Only amino acids located within 4 Å of the bound ligand are displayed (white sticks) and labeled. H12 is shown in slate. H-bonds discussed in the text are depicted as dashed black lines. The water molecules bridging the interaction of the ligand with the protein are displayed as red spheres. (**B**) Cα superposition of the complexes of PPARγ with compound **3a** and Rosiglitazone (a full agonist, magenta sticks, PDB code: 2PRG). The LBD of PPARγ consists of a hydrophobic entrance (arm III) that branches off into two subsites: the polar arm I, which is extended toward H12, and the hydrophobic arm II, which is located between helix H3 and β-sheet. The partial agonists occupy mainly arm II and arm III of the PPARγ LBD, but the full agonists occupy mainly arm I and arm II. (**C**) 2D ligand-interaction diagram of **3a**. Positively charged amino acids are represented with dark blue circles, polar amino acids are represented with light blue circles and hydrophobic amino acids are represented with green circles. Hydrogen bonds are depicted with purple arrows–dashed arrows for H-bonds involving amino acid side chain and regular arrows for H-bonds involving amino acid backbone.

Compounds **1a**, **1i** and **3a** were found to fit well within the PPARγ binding pocket. However, they assumed a binding mode different from that of Rosiglitazone and other known PPARγ agonists. The binding mode of **3a** as a representative example of all three compounds bound to PPARγ is shown in Fig. 4A. Although Rosiglitazone bind in a “U-shaped” conformation, wrapping around helix 3, our compounds bind in the LBD parallel to H3 (Fig. 4B). Given this binding mode, **1a**, **1i** and **3a** do not contact the three key residues, H323 at helix 5, H449 at helix 11, and Y473 from the AF-2 helix in arm I of the LBD, whose interaction with the acidic head groups of TZDs may contribute to the receptor activation. Instead, they lie between H3 and the β-sheet (arms II and III of LBD), an area which is preferentially occupied by synthetic partial agonists such as PA-082^54^, MRL-24, nTZDpa, and BVT.13^55^. Three water molecules participate in an indirect H-bond network that involves both the TZD 4-oxo group and the amide C=O group of the N3-alkyl substituent of three ligands and the NH backbone of (β-sheet), the C=O backbone of L340 (β-sheet) and the NE atom of R288. The TZD 2-oxo group makes an indirect water-mediated interaction with the OH group of Y327, with a distance of 3.0 Å. The remaining interactions between ligands and protein are basically hydrophobic. In particular, the thiazolidinone ring contacts residues C285 (H3), L330 (H5), V339 (β-sheet), I341 (β-sheet), and M364 (H8). Significant interactions between the TZD sulfur atom with the sulfurs of the two specific residues C285 and M364 are at the distances 2.8 and 3.3 Å, respectively. The N3-alkyl substituents occupy the arm III of the PPARγ LBD cavity and make hydrophobic contacts with E295 (H3), A292 (H3), R288, M329 (H5), I326 (H5), L330 and L333 (H5). The benzylidene ring reaches into the pocket below H5 and forms hydrophobic interactions with I281 (H3), F282, C285, M348 (β-sheet), L353 (H6), L356 (H6/H7 loop), F360 (H7), F363 and M364 residues. Moreover, it engages in a parallel-displaced π-stacking interaction with the aromatic ring of F363 of H7. It is interesting to note that residues L356 and F360 appear in a suitable position to make lipophilic contacts with the *p*-chlorine atom of compound **3a**, thus rationalizing the increase of efficacy of **3a** compared to **1a** and **1i**.

### Cytotoxicity of 1a, 1i, and 3a against NIH/3T3 cells cells

**Figure 5:**
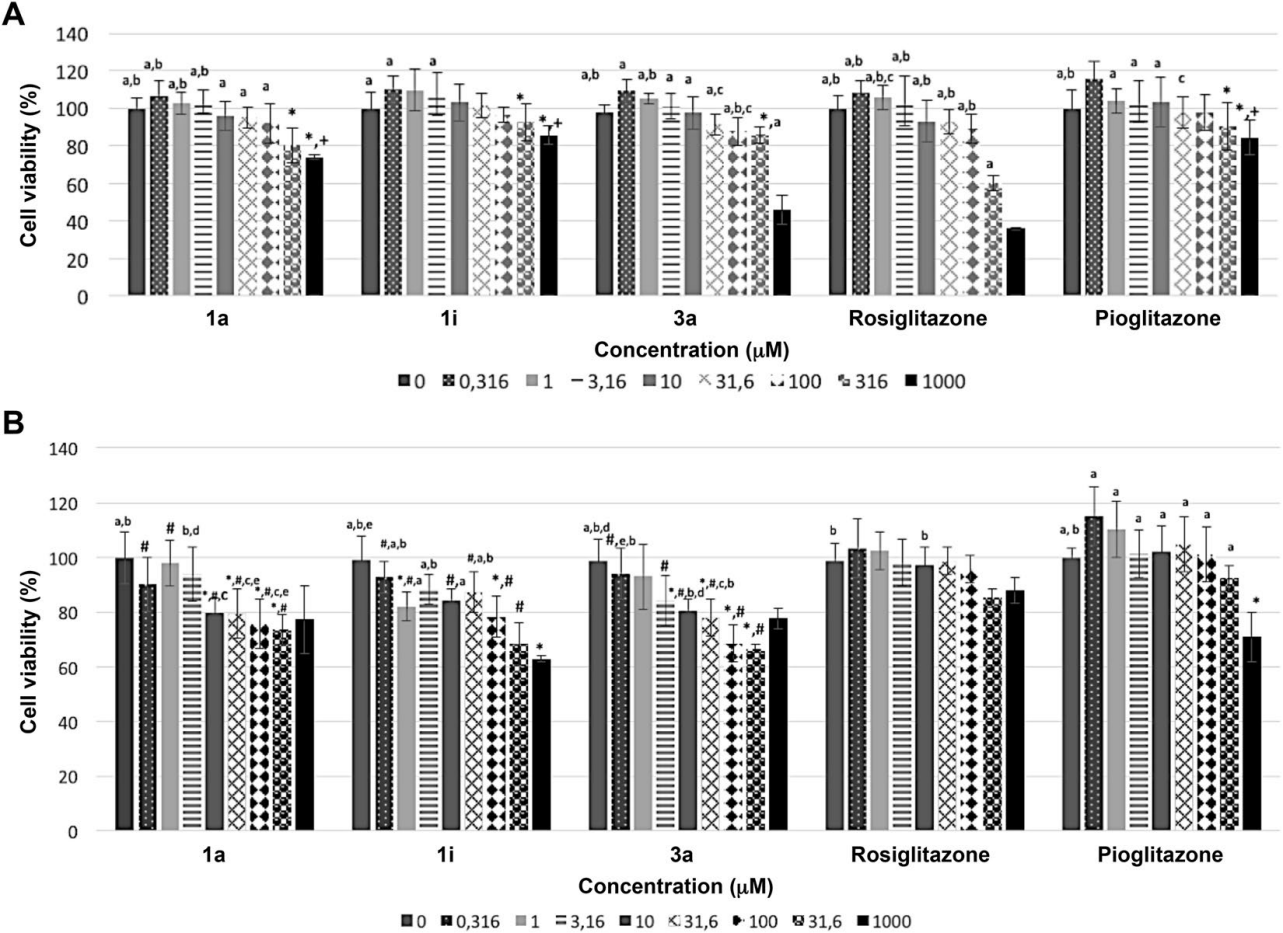
Cell viability % of **1a**, **1i** and **3a** against NIH/3T3 (**A**) and HepG2 (**B**) Cells. Data were analysed by two-way ANOVA followed by Tukey test and expressed as mean ± SEM. ^a^indicates *p* < 0.05 *vs* 1000 μM; ^b^indicates *p* < 0.05 *vs* 316 μM, ^c^indicates *p* < 0.05 *vs* control; ^d^indicates *p* < 0.05 *vs* 100 μM; ^e^indicates *p* < 0.05 *vs* 10 μM; *indicates *p* < 0.05 *vs* Rosiglitazone; ^+^indicates *p* < 0.05 *vs* **3a**; ^#^indicates *p* < 0.05 *vs* Pioglitazone.

The cytotoxic effects of BTZDs **1a**, **1i** and **3a** on NIH-3T3 cells were subjected to cytotoxicity evaluation by XTT method as reported earlier^56^ (Fig. 5A). All three compounds were found having cytotoxicity at doses near or above 1000 µM (Table 3), but did not show any apparent toxicity at their effective doses. **3a** was found to be the most potent among them, even though the three compounds shared very similar therapeutic values. According to cell viability data, **1a**, **1i** and **3a** provided significantly higher viability than Rosiglitazone *in vitro*, especially at 316 and 1000 μM (Fig. 5A). At these concentrations, all of the compounds decreased cell viability in a dose-dependent manner. Also, **1i** was the least cytotoxic compound among all of them. Therefore, **1a**, **1i** and **3a** are valuable in terms of their safety and efficacy according to the results of *in vitro* studies.

**Table 3:** Cytotoxicity data of compounds **1a**, **1i** and **3a**.

Compounds	EC_50_ (µM)	IC_50_ (µM)	IC_50_/EC_50_ (µM)
**1a**	7.00 ± 1.3	>1000	142.85
**1i**	9.60 ± 0.8	>1000	105.16
**3a**	5.90 ± 0.6	966 ± 10	163.73
**Rosiglitazone**	0.04 ± 0.02	662 ± 18	16550.00
**Pioglitazone**	0.65 ± 0.01	>1000	1538.46

### Hepatotoxicity assessment of compounds 1a, 1i and 3a against HepG2 cells

Given that the first generation TZDs for T2DM treatment, like ciglitazone and troglitazone, were found to be highly hepatotoxic and thus showed limited clinical application and in consequence were rapidly withdrawn from the market after reports of severe liver failure and death^57^, a very critical point for PPARγ ligands to be considered as gold standard for T2DM drug discovery is that they did not present hepatotoxicity. For this reason, we investigated the hepatotoxicity of **1a**, **1i** and **3a** on HepG2 cells in a concentration range of 0–1000 μM (Fig. 5B). HepG2 cells are considered as a useful model to study *in vitro* xenobiotic metabolism and liver toxicity. In particular, HepG2 cells are able to activate and detoxify xenobiotics and, they are the reason why these cells are intensively used in toxicology^58,59^. Hepatotoxicity was determined using the XTT assay. According to our results, all of the compounds had IC_50_ values greater than 1000 μM, which is far above their EC_50_ values. Among the tested compounds, Rosiglitazone and Pioglitazone were found to possess increased cell viability (Fig. 5B). At 1000 μM, **1a** and **3a** did not show any significant difference compared with Rosiglitazone and also reached higher viability values than Pioglitazone. Compound **1i** only slightly reduced cell viability (63.97 ± 3.93%) at the highest tested concentration. Thus, **1a**, **1i** and **3a** did not possess any hepatotoxicity risk at the tested concentrations, exhibiting a wide therapeutical safety range.

### Antidiabetic activity of compounds 1a, 1i and 3a on STZ-NA-induced type 2 diabetic rats

**Figure 6:**
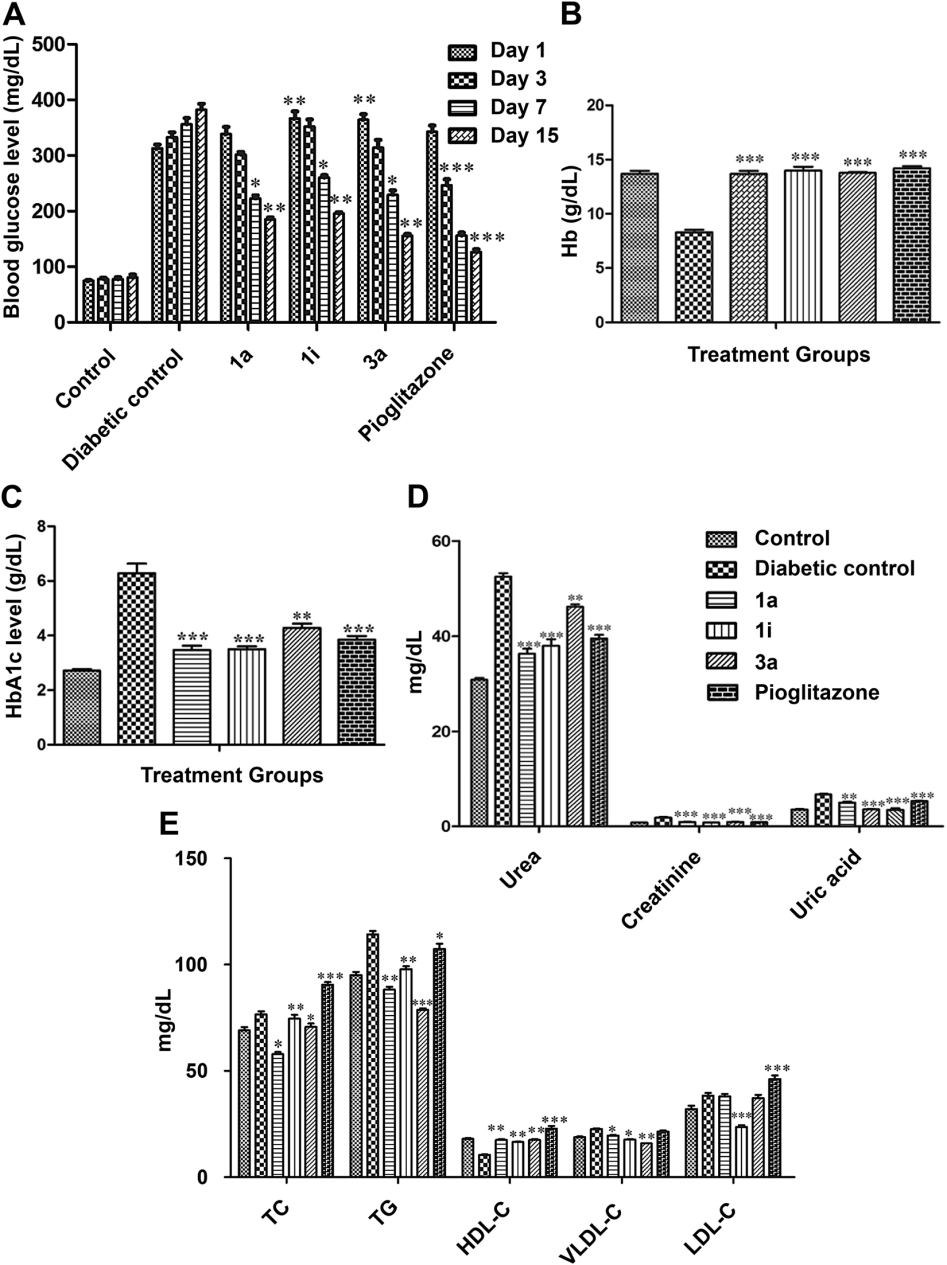
*In vivo* antidiabetic activity of **1a**, **1i**, **3a** in STZ-NA induced diabetic rats. Effects of compounds **1a**, **1i**, **3a**, and Pioglitazone on the blood glucose levels (**A**), Hb (**B**), HbA1c (**C**), serum urea, creatinine, and uric acid (**D**), and TC, TG, LDL and VLDL (**E**). Data were analysed by one way ANOVA followed by Dunnett’s ‘t’ test and expressed as mean ± SEM from six observations; ^***^indicates *p* < 0.001 *vs* diabetic control, ^**^indicates *p* < 0.01 *vs* diabetic control.

PPARγ partial agonists **1a**, **1i** and **3a** were investigated for their antidiabetic activity in a Streptozotocin-Nicotinamide (STZ-NA)-induced diabetic rat model. The test dose was fixed based on the predicted LD_50_ value^60^. A dose that is less than 1/20^th^ of predicted LD_50_ was used for the study. Diabetic rats were orally treated with the test compounds at a dose of 36 mg/kg body weight for a period of 15 days. Fasting blood glucose of control, diabetic-untreated and diabetic-treated rats were tested on Days 1, 3, 7 and 15 of the treatment period (Fig. 6A).

After administration for 15 days, the blood glucose levels of diabetic rats treated with compounds **1a**, **1i**, **3a**, and Pioglitazone were significantly reduced compared with the blood glucose levels of the diabetic control rats. The results of *in vitro* study were consistent with *in vivo* results indicating that these compounds exerted the blood glucose lowering effect by activating PPARγ receptors. In particular, the data for 15^th^ Day showed that the activity of compound **3a** was equivalent to the standard drug Pioglitazone used in the study.

Total hemoglobin (Hb), Glycosylated hemoglobin (HbA1c), lipid profile [total cholesterol (TC), triglyceride (TG), high-density lipoprotein cholesterol (HDL-C), low-density lipoprotein cholesterol (LDL-C) and very low-density lipoprotein cholesterol (VLDL-C)], urea, creatinine and uric acid levels were also measured before sacrificing the animals. Hb levels were significantly decreased in disease control (STZ + NA) when compared to normal control. **1a**-, **1i**-, **3a**- and Pioglitazone-treated groups showed a significant increase in Hb levels (Fig. 6B). HbA1c is a reliable index of glycemic control in diabetes^61^. Glycosylation of Hb is directly proportional to fasting blood glucose levels^62^. In this study, STZ-NA-induced diabetic rats showed high levels of HbA1c compared to normal control. Administration of **1a**, **1i** and **3a** significantly prevented the increase in HbA1c and this could be due to decrease in glucose levels (Fig. 6C). STZ-NA diabetic animals also displayed increased plasma levels of renal function parameters such as urea, creatinine, and uric acid, when compared to normal control. It was observed that all the active compounds significantly (p < 0.001) diminished the levels of urea, creatinine and uric acid compared to the mean values of diabetic control group (Fig. 6D). Diabetes mellitus is also associated with profound alterations in the plasma lipid profile^63^. An elevation in plasma concentrations of TG and LDL-C associated with marked reduction of VLDL-C are most typical feature of T2DM. Compared to normal control, disease control (STZ + NA) showed a significant increase in TC, TG, VLDL-C and LDL-C levels and a decrease in HDL-C level. The **1a**-, **1i**-, and **3a**-treated groups showed an increase in HDL-C and a significant decrease in TC, T, VLDL-C, and LDL-C when compared to disease control (STZ + NA) (Fig. 6E). Collectively, these data clearly indicate the antidiabetic potential of BTZD derivatives **1a**, **1i** and **3a**.

**Figure 7:**
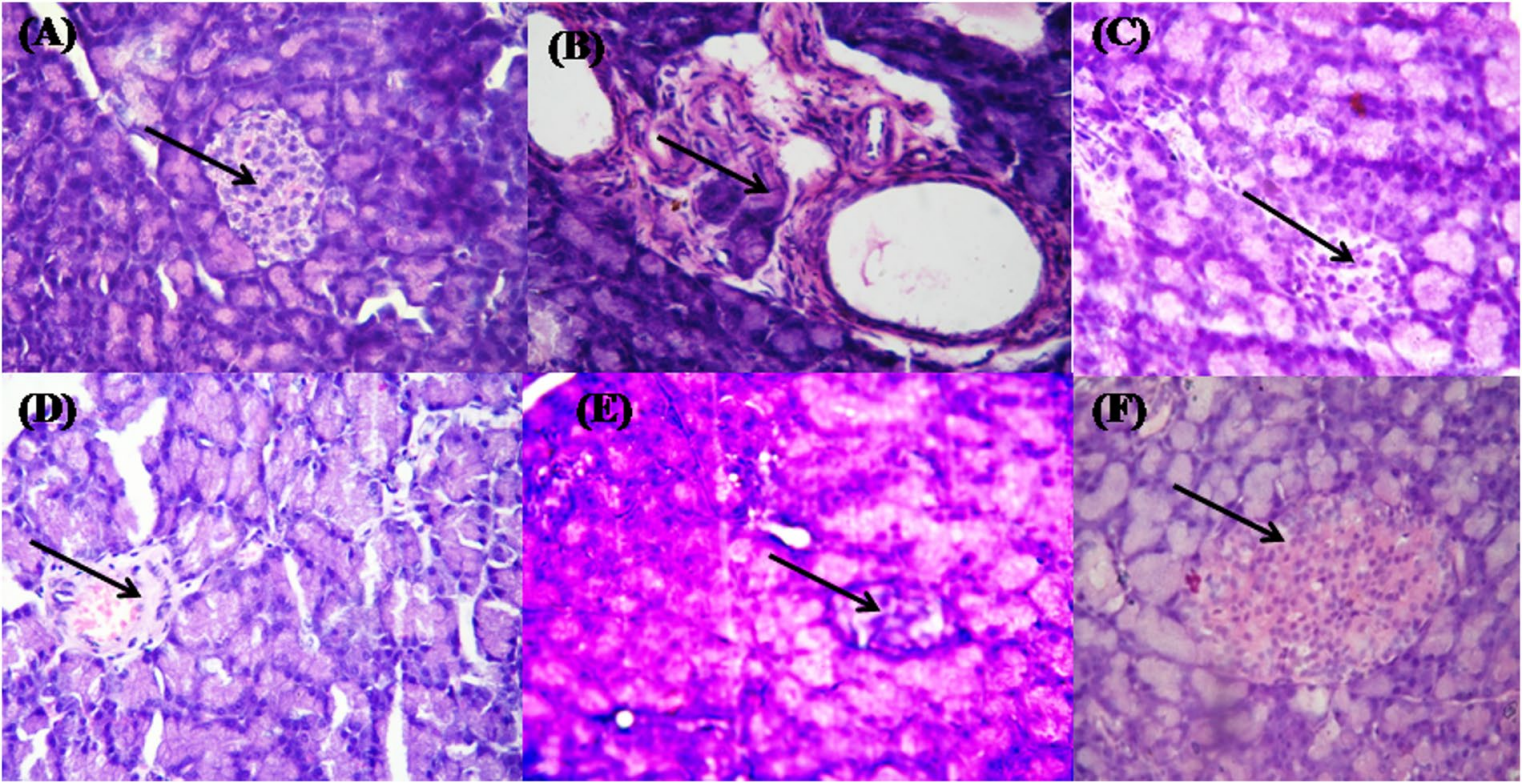
Histopathological changes in the pancreatic islets of normal and STZ-NA-induced diabetic rats. (**A**) Pancreatic islet architecture of the normal control rats. (**B**) STZ-NA-induced diabetic control rats show pancreatic islet damage. (**C**) STZ-NA-induced diabetic rats treated with compound **1a** (36 mg/kg body weight); (**D**) STZ-NA induced diabetic rats treated with compound **1i** (36 mg/kg body weight); (**E**) STZ-NA-induced diabetic rats treated with compound **3a** (36 mg/kg body weight); (**F**) STZ-induced diabetic rats treated with Pioglitazone (36 mg/kg body weight). Examinations were carried out at 40x magnifications with hematoxylin-eosin’s stain.

**Figure 8:**
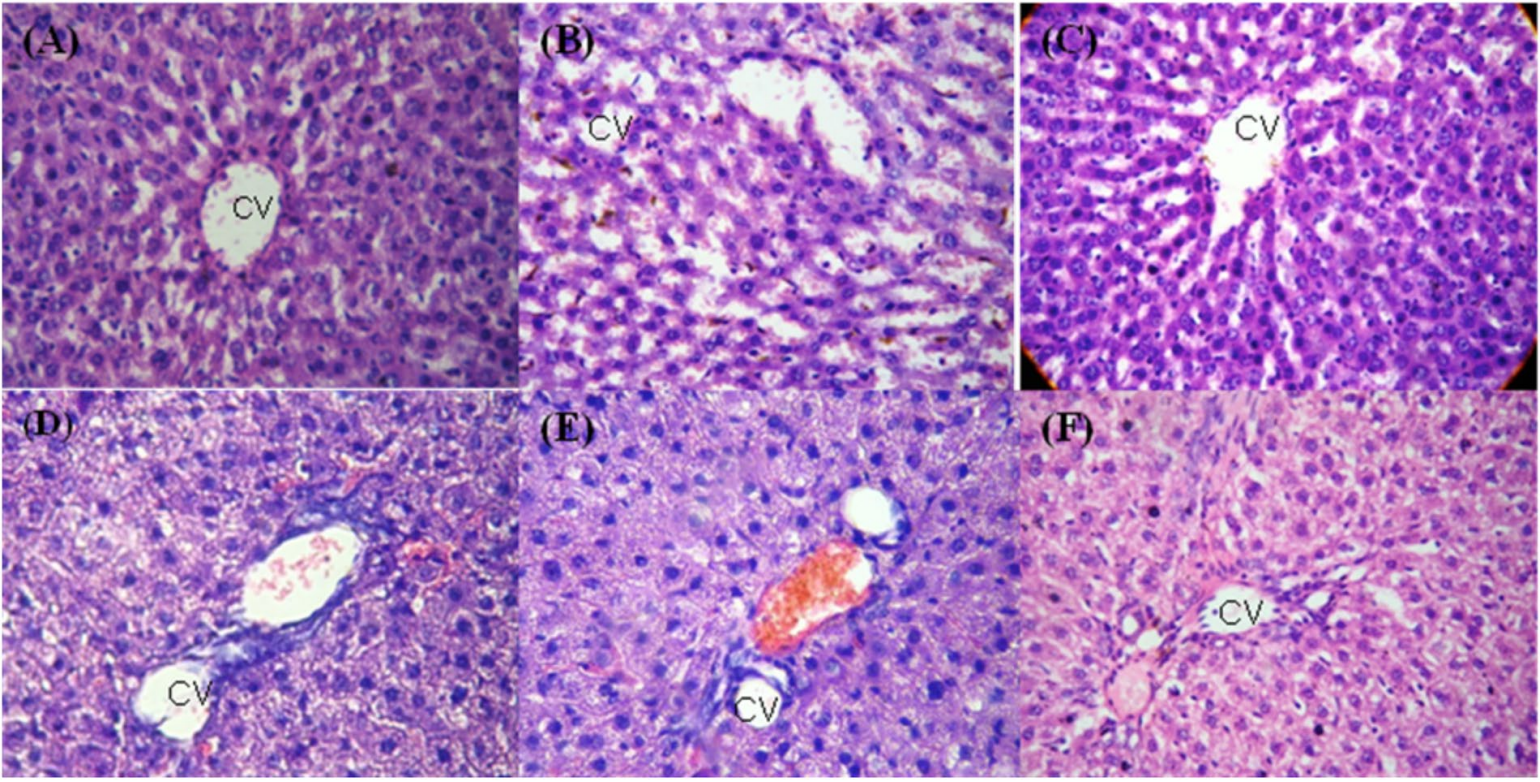
Histopathological changes in the liver of normal and STZ-NA-induced diabetic rats. (**A**) Normal Control. (**B**) Diabetic control. (**C**) STZ-NA-induced diabetic rats treated with compound **1a** (36 mg/kg body weight); (**D**) STZ-NA-induced diabetic rats treated with compound **1i** (36 mg/kg body weight); (**E**) STZ-NA-induced diabetic rats treated with compound **3a** (36 mg/kg body weight); (**F**) STZ-induced diabetic rats treated with Pioglitazone (36 mg/kg body weight). Examinations were carried out at 40x magnifications with hematoxylin-eosin’s stain.

A histological study was carried out by using light microscopy in order to detect whether the treatment with **1a**, **1i** and **3a** had an effect on the STZ-NA-induced diabetic pancreas and liver tissues. The histopathological examination of untreated diabetic rats showed a significant damage to pancreatic islets and degranulation of β-cells when compared to normal control pancreatic histology (Fig. 7), which had normal proportion of islets of Langerhans and acinar cells. The histological changes in diabetic rats were restored to near normal by treatments with **1a**, **1i**, **3a** and Pioglitazone. In case of liver histopathological studies in experimental rats, the liver’s histological structure (Fig. 8A) was normal in the control group, with a central vein and surrounding hepatocytes. Figure 8B shows a liver with clear hepato-cellular necrosis and marked vacuolization with reducing nuclei size and disordered liver structure in STZ-NA-induced diabetic rats. Rats treated with **1a**, **3a** (Fig. 8C and E) and Pioglitazone (Fig. 8F) showed no hepatic abnormalities, and the arrangements of the hepatocytes in the liver were almost normal. In case of **1i**-treated rats (Fig. 8E), the damage to the normal hepatocytes was observed more when compared with normal group.

### Pharmacokinetic profile of 1a, 1i and 3a

Aiming to identify if **1a**, **1i** and **3a** could be suitable candidates for *in vivo* experiments, we profiled them in pharmacokinetic assays *in vitro*. The oral bioavailability of compounds in terms of Caco-2 and MDCK-II permeability and the P-glycoprotein (P-gp) efflux ratio were assessed^64^. The *in vitro* intrinsic half-life (T_1/2,int_) and the apparent intrinsic clearance (CL_int,app_) of compounds were also measured after incubation with human liver microsomes at 37.5 °C^65^. Apparent permeability coefficients (P_app_) from A-to-B (apical to basolater of the cell monolayers) and B-to-A (basolateral to apical of the cell monolayers) were obtained by measuring the amount of the compound transported from the donor compartment at 2 μM to the receiver compartment after 150 min incubation. Quantification was done by LC-MS/MS. P-gp substrate activity was assessed by the efflux ratio (the ratio of P_app_ in the B-to-A direction over that in the A-to-B direction). As showed in Tables 4 and 5, compound **1i** exhibited asymmetric transport across both Caco-2 and MDCK-II monolayers (P_app,A–B_ of 23.0 × 10^−6^ cm/sec for Caco-2 and 16.3 × 10^−6^ cm/sec for MDCK-II; P_app,B–A_ of 51.2 × 10^−6^ cm/sec for Caco-2 and 11.9 × 10^−6^ cm/sec for MDCK-II), suggesting that it may be orally absorbed in both mouse model and human studies. An efflux ratio (P_app,B–A_/P_app,A–B_) of >2 indicates that a compound may be a potential substrate for permeability glycoprotein (P-gp) or other active transporters. P-gp is an ATP-dependent transporter on the apical plasma membrane of enterocytes that functions to limit the entry of drugs into the cell. Compounds **1a**, **1i** and **3a** showed efflux ratios less than 2.0 in MDCK-II cells, indicating that they are not potential substrates for P-gp and therefore have improved potential for oral absorption. In contrast, the efflux ratios were slightly higher than 2.0 in Caco-2 cells, suggesting the three compounds retained weak P-gp substrate activity.

**Table 4:** *In vitro* Caco-2 permeability data of compounds.

Compound	Avg P_app_ × 10^−6^ cm/sec; n = 2			
	A to B	B to A	Efflux Ratio (B > A/A > B)	Permeability Rank^1^
**1a**	2.4	14.3	2.1	Low
**1i**	23.0	51.2	2.7	High
**3a**	1.6	19.3	3.2	Low

All permeability values are represented as Avg P_app_ × 10^−6^ cm/sec. ^1^Permeability ranking is based on following criteria (in hours): Low, P_app,A-B_ < 5 × 10^−6^ cm/sec; High, P_app,A-B_ ≥ 5 × 10^−6^ cm/sec. Cell growth media, DMEM, 10% FBS, Gentamycin; test conc: 2 µM; incubation period, 150 min; apical/donor pH, 6.5/7.4.

**Table 5:** *In vitro* MDCK-II permeability data of compounds.

Compound	Avg P_app_ × 10^−6^ cm/sec; n = 2			
	A to B	B to A	Efflux Ratio (B > A/A > B)	Permeability Rank^1^
**1a**	1.8	2.5	1.4	Low
**1i**	16.3	11.9	0.7	High
**3a**	0.8	0.4	0.5	Low

All permeability values are represented as Avg P_app_ × 10^−6^ cm/sec. ^1^Permeability ranking is based on following criteria (in hours): Low, P_app,A-B_ < 5 × 10^−6^ cm/sec; High, P_app,A-B_ ≥ 5 × 10^−6^ cm/sec. Cell growth media, MEM-alpha with MEM NEAA-glutamine-penicillin-streptomycin; test conc: 2 µM; incubation period, 150 min; apical/donor pH, 7.4/7.4; NA, not applicable.

Since the liver is the primary site of drug metabolism, subcellular fractions of liver microsomes are useful *in vitro* models of hepatic clearance as they still contain many of the drug metabolizing enzymes found in the liver (notably, the cytochrome P450 enzymes). Highly cleared compounds *in vitro* are likely to be rapidly cleared *in vivo* resulting in a short duration of action. In the human liver microsomal stability study, compound **1i** was found to possess T_1/2,int_ of 92.55 min, with an *in vitro* CL_int,app_ of 18.71 µL/min/mg, which is an indicator of good metabolic stability (Table 6). In contrast, compound **1a** exhibited low metabolic stability with T_1/2,int_ of 10.20 min and CL_int,app_ of 169.81 µL/min/mg.

**Table 6:** *In vitro* Human liver microsomal stability data.

Compounds	T_1/2,int_ (min)	CL_int,app_ (µL/min/mg)
**1a**	10.20	169.81
**1i**	92.55	18.71
**3a**	53.29	32.50

CL_int,app_ range: 14.4–577.5 µL/min/mg; T_1/2,int_ range: 3–120 min; LM conc: 0.4 mg/mL.

## Discussion

PPARγ is the pharmacological target of the insulin-sensitizing TZDs that have been widely used in the treatment of T2D. TZDs function as selective PPARγ ligands and induce transcription of PPARγ-targeted genes^20^. However, they are associated with various undesirable and severe side effects, including weight gain, fluid retention, edema, congestive heart failure, and bone fracture^25–27^. These limitations have raised substantial alarms and significantly compromised their future in many countries^28^. Therefore, it is critical to develop novel partial PPARγ agonists or SPPARγM that retain effective insulin-sensitizing action while minimizing the potential side effects, as demonstrated by recent studies in animal models and in clinical trials^29–34^.

In this study, we synthesized and gave the structural characteristics of a series of new BTZD derivatives bearing a substituent on the nitrogen atom of TZD nucleus, namely compounds **1a**-**1k**, **2i**-**10i**, **3a**, **6a**, and **8a**-**10a**. Transactivation assays and SPR-based Biacore determinations indicated that compounds **1a**, **1i**, and **3a** bound to PPARγ with low affinity compared with the well-characterized PPARγ agonists Rosiglitazone and Pioglitazone. The partial PPARγ agonist activity of these compounds may offer a distinct benefit because a number of studies have shown that partial PPARγ agonists, including selective SPPARγM, generate fewer side effects than full agonists^29–34^.

Docking experiments revealed that **1a**, **1i**, and **3a** interact with PPARγ through a distinct binding mode, utilizing a north-south orientation, parallel to H3. Unlike full agonists, such as the TZDs or tyrosine analogs, BTZD derivatives show no direct H-bond interactions with the AF2 helix in arm I portion of the binding pocket but instead lie between H3 and the β-sheet, extending from arm II to arm III of the LBD. **1a**, **1i**, and **3a** stabilize H3 and the β-sheet of PPARγ through water-mediated interactions between their TZD 4-oxo groups and both the NH backbone of S342 and the C=O backbone of L340. A further water-mediated H-bond is formed between the TZD 2-oxo group of ligands and the Y327 side chain. Moreover, compounds make numerous hydrophobic and van der Waals contacts with side chains of residues from H3, H5, H6 and H7.

Co-crystallography, mutagenesis and hydrogen/deuterium exchange (HDX) studies have all displayed that PPARγ full agonists activate the receptor through direct interactions with residues H323, H449 and Y473^55,66–68^. These interactions place the AF2 helix in a conformation that facilitates the recruitment of coactivator proteins and the activation of the transcriptional machinery. In contrast, partial agonists such as PA-082^54^, MRL-24, nTZDpa, and BVT.13^55^ activate PPARγ by a H12-independent mechanism^55,69,70^, i.e they do not bind to PPARγ by the network of H-bonds used by full agonists but preferentially stabilize other regions of the LBD, especially H3 and the β-sheet region, and/or promote modifications in the structure and dynamics of the flexible region known as Ω-loop^71–74^. This causes a lower degree of H12 stabilization, which affects the recruitment of coactivators and, in turn, decreases the transcriptional activity of PPARγ^52,55^. The lack of direct interaction between the H12 helix of the receptor and BTZD derivatives together with the stabilization of H3 and the β-sheet of PPARγ may explain the partial agonist/modulator activities of **1a**, **1i**, and **3a** observed *in vitro*.

The results of our *in vitro* studies showed that compounds **1a**, **1i**, and **3a** not only weakened toxicity on NIH-3T3 cells compared with the full PPARγ agonist Pioglitazone but also exhibited no hepatotoxicity risk at the tested concentrations. The antidiabetic and antilipidaemic activity of BTZDs were also evaluated by *in vivo* experiments in a STZ-NA-induced diabetic rat model. This disease model is characterized by moderate stable hyperglycemia, glucose intolerance, and reduced pancreatic insulin stores, and shares a number of syndromes with T2D patients^75^. In this study, administration of **1a**, **1i**, and **3a** for 15 days in STZ-NA-induced diabetic rats significantly decreased serum blood glucose compared to control diabetic rats after 15 days of treatment. Compound **3a** showed a glucose-lowering effect similar to Pioglitazone. Moreover, BTZDs-treated diabetic rats significantly decreased the level of HbA1C and increased Hb, which might be the result of an improvement in the glucose metabolism. Serum urea, uric acid and creatinine level demonstrate the role of **1a**, **1i**, and **3a** in renal dysfunction. An augmented level of urea, uric acid and creatinine cause the osmotic diuresis and depletion of extracellular fluid volume in the diabetic condition in the serum of STZ-NA-induced diabetic rats^76^. **1a**, **1i**, and **3a** treated groups showed a declined level of urea, uric acid, and creatinine, thus revealing a their positive effect in renal dysfunction.

It is well known that augmentation in TC and TG, LDL-C, VLDL-C, and the decline in the HDL-C are regarded as most common abnormalities in disease control (STZ + NA) group. In our study, the diabetic rats treated with **1a**, **1i**, and **3a** showed an elevation in HDL-C and reduction in LDL-C and VLDL-C as evidenced by decreased levels of TC and TG, confirming the positive role of these compounds in increased insulin action on cholesterol and fatty acid biosynthesis and intestinal cholesterol absorption.

Histopathological findings of the pancreas and liver segments of BTZDs-treated diabetic rats revealed numerous pathological signs. Our findings demonstrated that the treatment of STZ-NA-induced diabetic rats with **1a**, **1i** and **3a** restored to nearly normal the islets of Langerhans containing β-cells. Distorted central vein and hepatocytes were reinstated to normal in liver of STZ-NA-induced diabetic treated rats when compared to the toxic control. A good metabolic stability in liver microsomes, optimum Caco-2 and MDCK-II permeability, and pharmacokinetic profile in preclinical species are the major characteristics for a potential and safe drug molecule that could be further extrapolated in humans before clinical trials. In the present study, we tried to establish the *in vitro* pharmacokinetics, permeability, and efflux concerns for compounds **1a**, **1i** and **3a**. Our *in vitro* findings indicated that **1i** displays a good metabolic stability in human liver microsomes (T_1/2,int_ = 92.55 min and CL_int,app_ = 169.81 μL/min/mg), and a high Caco-2 and MDCK-II permeability with small efflux ratio. Taken together, as selective partial PPARγ agonists, BTZD derivatives **1a**, **1i** and **3a** improved not only the homeostasis of glucose metabolism, but also that of the lipid metabolism, suggesting that these compounds are very promising pharmacological agents by selectively targeting PPARγ for further development in the clinical treatment of T2D.

## Experimental Procedures

### Synthesis of the BTZD derivatives

All the synthetic work was done procuring required laboratory and analytical grade chemicals, reagents and solvents. All reactions were carried out in oven-dried glassware under nitrogen atmosphere. Chemicals and solvents were of reagent grade and purchased from Sigma Aldrich/Merck/Spectro-chem/CDH. Completions of reactions were monitored on pre-coated TLC plates (silica gel 60 F-254, Merck™, KGaA, Germany). Melting points were determined on an OPTIMELT automated system apparatus and were uncorrected. Intermediates were characterized by their melting points. Compounds were purified by recrystallization using suitable solvents. Final compounds were characterized by their ^1^H NMR (300 MHz, ECX-500, JEOL and 400 MHz, VNMRS400), ^13^C NMR (400 MHz, VNMRS400), in either CDCl_3_ or DMSO-*d*_6_ as a solvent. Mass spectra were recorded by WATERS-Q-T of Premier-HAB213 using the electrospray ionization-mass spectrometry (ESI-MS) technique. General procedure for the preparation of the BTZD derivatives, as well as NMR-H, NMR-C^13^ and ESI-MS spectra of compounds **1a**-**k**, **3a**, **6a**, **8a**-**10a**, **2i**-**10i** were supplied in Supplementary Material (Figs. S4–S77).

### Transcriptional transactivation assay

Reference compounds, the medium, and other cell culture reagents were purchased from Sigma-Aldrich (Milan, Italy).

#### Plasmids

The expression vectors expressing the chimeric receptor containing the yeast Gal4-DNA binding domain fused to either the human PPARα, PPARγ, or PPARδ LBD and the reporter plasmid for these Gal4 chimeric receptors (pGal5TKpGL3) containing five repeats of the Gal4 response elements upstream of a minimal thymidine kinase promoter that is adjacent to the luciferase gene were described previously^77^.

#### Cell culture and transfections

Human hepatoblastoma cell line HepG2 (Interlab Cell Line Collection, Genoa, Italy) was cultured in minimum essential medium (MEM) containing 10% of heat-inactivated foetal bovine serum, 100 U of penicillin G/mL, and 100 μg of streptomycin sulfate/mL at 37 °C in a humidified atmosphere of 5% CO_2_. For transactivation assays, 10^5^ cells per well were seeded in a 24-well plate and transfections were performed after 24 h with CAPHOS, a calcium phosphate method, according to the manufacturer’s guidelines. Cells were transfected with expression plasmids encoding the fusion protein Gal4-PPARα-LBD, Gal4-PPARγ-LBD, or Gal4-PPARδ-LBD (30 ng), pGal5TKpGL3 (100 ng), and pCMVβgal (250 ng). Four hours after transfection, cells were treated for 20 h with the indicated ligands and reference compounds in duplicate. As a preliminary assay, all compounds were tested at two concentrations (5 and 25 μM); afterwards, only for ligands showing weak or moderate efficacy a concentration-response curve was carried out. Luciferase activity in cell extracts was determined by a luminometer (VICTOR^3^ V Multilabel Plate Reader, PerkinElmer). β-Galactosidase activity was determined using ortho-nitro-phenyl-β-D-galactopyranoside as described previously^78^. All transfection experiments were repeated at least twice.

### Surface Plasmon Resonance

SPR analyses were carried out on a BIACORE 3000 instrument (GE-Healthcare). A PPARγ, surface was generated using a research-grade CM5 sensor chips (GE Healthcare). The protein (100 µg/ml in 10 mM CH_3_COONa, pH 5.0) was immobilized using a standard amine-coupling protocol, to obtain densities of 4 kRU. Testing compounds were dissolved in 100% DMSO to obtain 10 mM solutions, and then diluted in PBS containing variable amounts of DMSO, achieving a final DMSO concentration of 0.1%. For each molecule a five-point concentration series (0.5 µM, 1 µM, 2,5 µM, 5 µM and 10 µM) was set up. SPR experiments and data elaboration were carried out as reported elsewhere^48^.

### Computational chemistry

Molecular modeling and graphics manipulations were performed using Maestro 10.5 (Schrödinger, LLC, New York, NY, 2016) and UCSF-CHIMERA 1.8.1 software packages, (http://www.cgl.ucsf.edu/chimera), running on an E4 Computer Engineering E1080 workstation E4 Computer Engineering E1080 workstation provided with an Intel Xeon processor. GOLD Suite 5.4.1 docking package (CCDC Software Limited: Cambridge, U.K., 2008)^49^ was used for all docking calculations. Figures were generated using Pymol 1.8.2 (Schrödinger, LLC, New York, NY, 2016).

#### Protein and Ligand Preparation

The coordinates of PPARγ in complex with GQ-16 (PDB code: 3T03)^32^, recovered from Brookhaven Protein Database^79^, were employed for the automated docking experiments. The protein setup was carried out using the Protein Preparation Wizard implemented in Maestro. Hydrogen atoms were added to the protein consistent with the neutral physiologic pH. Arginine and lysine side chains were considered as cationic at the guanidine and ammonium groups, and the aspartic and glutamic residues were considered as anionic at the carboxylate groups. The protonation and flip states of the imidazole rings of the histidine residues were adjusted together with the side chain amides of glutamine and asparagine residues in a H-bonding network optimization process. Successively, the protein hydrogens only were minimized using the Impref module of Impact with the OPLS_2005 force field. Initial coordinates of compounds **1a**, **1i** and **3a** were constructed by using the Molecular Builder module in Maestro. The structures were energy-minimized using Macromodel 10.8 (Schrödinger, LLC, New York, NY, 2016) using the MMFF force field with the steepest descent (1000 steps) followed by truncated Newton conjugate gradient (500 steps) methods. Partial atomic charges were computed using the OPLS-AA force field.

#### Docking Simulations

Docking of **1a**, **1i** and **3a** to PPARγ was performed with the GOLD software, which uses a genetic algorithm (GA) for determining the docking modes of ligands and proteins. The coordinates of the cocrystallized ligand GQ-16 were chosen as active-site origin. The active-site radius was set equal to 10 Å. Four explicit water molecules were allowed to toggle on or off during the individual docking runs (i.e., these waters were not automatically present in the binding site, but were included if their presence strengthened the interaction of the ligand with the receptor, as determined by the scoring function)^80^. Mobility of R288 side chain was established using the flexible side chains option in the GOLD front end, which incorporates the Lovell rotamer library^81^. Each GA run used the default parameters of 100 000 genetic operations on an initial population of 100 members divided into five subpopulations, with weights for crossover, mutation, and migration being set to 95, 95, and 10, respectively.

GOLD allows a user-definable number of GA runs per ligand, each of which starts from a different orientation. For these experiments, the number of GA runs was set to 1000 without the option of early termination, and scoring of the docked poses was performed with the original ChemPLP scoring function rescoring with ChemScore^51^. The final receptor-ligand complex for each ligand was chosen interactively by selecting the highest scoring pose that was consistent with the experimentally derived information about the binding mode of the ligand.

### Cytotoxicity assessment

The 2,3-bis(2-methoxy-4-nitro-5-sulfophenyl)-2H-tetrazolium-5-carboxanilide (XTT) assay is based on the mitochondrial succinate dehydrogenase activity, which is only active in cells with an intact metabolism. XTT is reduced to soluble formazan by this enzyme and formazan is quantified and directly proportional to the number of viable cells. NIH/3T3 mouse embryonic fibroblast (ATCC^®^CRL-1658™) cell line was used to investigate the cytotoxicity of the compounds. NIH/3T3 cells were incubated according to the instructions of the supplier at 37 °C in a humidified atmosphere of 95% air and 5% CO_2_. NIH/3T3 cells were seeded at 1 × 10^4^ cells into each well of 96-well plates. After 24 hours of incubation, compounds were added to the wells at the concentration range between 1 mM and 0,000316 mM concentrations (1000; 316; 100; 31.6; 10; 3.16; 1; 0.0316 µM) in quadruplicates. The XTT assay (Xenometrix, Allschwil, Switzerland) was performed according to the manufacturer’s instructions, after 24 h incubation with the compounds. The absorbance was determined after 2 h incubation at 480 nm with a reference wavelength of 680 nm using a microplate reader (BioTek, USA). Inhibition % was calculated for each concentration of the compounds according to the formula below obtained from the instructions of the manufacturer and IC_50_ values were estimated by plotting a dose response curve of the inhibition % versus test compound concentrations. IC_50_/EC_50_ values were calculated, values >1000 μM were accepted as 1000. Cell viability % was calculated according to growth control absorbance values.

$$ \% \,Inhibition=100-\,\frac{(Corrected\,mean\,OD\,sample)}{Corrected\,mean\,OD\,solvent\,controls}\times 100$$

The stock solutions of the compounds were prepared in DMSO and further dilutions were made with fresh culture medium. The final DMSO concentration was under 0.1%. Rosiglitazone and Pioglitazone were used as positive controls. All data were obtained from 3 independent experiments in quadruplicates.

### *In vitro* hepatotoxicity assessment

In order to test the compounds for their hepatotoxicity, XTT assay was carried out on HepG2 human hepatocellular liver carcinoma cell line (ATCC® HB-8065™) as shown above. Cells were incubated according to the instructions of the supplier at 37 °C in a humidified atmosphere of 95% air and 5% CO_2_ and they were seeded at 1 × 10^4^ cells into each well of 96-well plates. After 24 hours of incubation, eight different concentrations of the compounds were added to the wells (1000; 316; 100; 31.6; 10; 3.16; 1; 0.316 µM) in quadruplicates. The final DMSO concentration was set at <0.1%. Rosiglitazone and Pioglitazone were used as positive controls. IC_50_ values and cell viability % were calculated.

### Animals and drug treatments

Male Wistar strain rats weighing about 160–200 g were used for animal studies. The experimental protocol was approved by the institutional animal ethical committee (no. PROV/BIT/PH/IAEC/04/2016) of the Department of Pharmaceutical Sciences and Technology, Birla Institute of Technology, Mesra, Ranchi, in accordance with National Institutes of Health Guide for Care and Use of Laboratory animals. Animals were housed in polypropylene cages containing wood shaving as bedding material, and maintained in the departmental animal house at 26 ± 2 °C and 44–55% relative humidity with a natural light/dark cycle, respectively. Rats were provided with rodent diet and water *ad libitum*. After initial duration of 12 h fast, the animals were rendered diabetic by a single intraperitonial administration of NA in normal saline at a dose of 230 mg/kg body weight, that was followed by freshly prepared solution of STZ in 0.1 M citrate buffer (pH 4.5) at a dose of 60 mg/kg body. The animals were allowed to drink 5% glucose solution overnight to prevent STZ-NA-induced hypoglycemia^82,83^. The rats were considered as diabetic if their blood glucose levels were above 250 mg/dL on the 3^rd^ day after STZ-NA injection. The rats were divided into four groups of six animals in each group. Control animals received normal saline (Group I). Diabetic rats received STZ-NA injection (Group II). Diabetic rats orally fed with standard (Pioglitazone) and test drugs (**1a**, **1i** and **3a**) as 0.25% suspension in carboxymethyl cellulose at a dose of 36 mg/kg for 15 days (Groups III and IV). The blood glucose level of each group was checked at 1, 3, 7 and 15 Day by withdrawing blood from tail vein of the animal using glucose meter (ACCu-Chek active, Roche, Diagnostics USA).

#### Biochemical determinations

After pharmacological screening for antidiabetic activity, the animals were subjected to overnight fasting and blood was collected from retro-orbital region under light anaesthesia for performing various biochemical experiments. All the biochemical experiments were performed using commercially available kits. Total Hb was estimated by cyanomethaemoglobin method^84^, while HbA1c was estimated from whole blood using commercially available kits following the manufacturer guidelines (ERBA Diagnostics Inc, Accurex Biomedical Pvt. Ltd., Coral clinical systems, India). TG concentration was determined by GPO-POD enzymatic-colorimetric method^85^. TC level was measured by the end point, CHOD-POD colorimetric methods^86^. HDL-C was assayed by the method reported earlier^87^. VLDL-C and LDL-C in plasma were calculated by the Friedewald formula: VLDL-C = TG/5; LDL-C = TC-(HDL-C + VLDL-C)^88^. Levels of serum urea, uric acid, and creatinine were measured using commercially available kits^89^.

#### Histopathological study

Rats were sacrificed under light anesthesia using diethylether at the end of the pharmacological and biochemical studies. Pancreas and liver tissues were dissected, washed in ice cold physiological saline, fixed in a 15% buffered neutral formalin solution and, finally, embedded into paraffin blocks. Then, the tissue was sliced out into sections of 5 µm thickness by a rotator microtome and stained with hematoxylin-eosin. Obtained sections were examined by a Leica DME microscope, and representative photomicrographs were taken by a 7.1 megapixel Canon Power Shot S70 digital camera.

### Pharmacokinetic profiling

The three active compounds **1a**, **1i** and **3a** were subjected to *in vitro* Caco-2 and MDCK-II permeability studies, as well as metabolic stability assays based on earlier reported procedures^64,65^.

### Statistical analysis

Statistical analysis was carried out using GraphPad Prism 5.0 software for windows (San Diego, California, USA). Results were expressed as mean ± S.E.M. (standard error of the mean) for six rats in each group. The data was analysed by one way analysis of variance (ANOVA) followed by Dunnett’ multiple variance test. Differences were considered statistically significant at the levels of P < 0.05.

## Electronic supplementary material

Supplementary Material
