# Supplementary material for: Novel Benzylidene Thiazolidinedione Derivatives as Partial PPARγ Agonists and their Antidiabetic Effects on Type 2 Diabetes
Source: Sci Rep. 2017 Oct 31;7:14453. doi: 10.1038/s41598-017-14776-0 (PMC5663708; doi:10.1038/s41598-017-14776-0)
Supplement: Supplementary file 1 — Supplementary Material [file 41598_2017_14776_MOESM1_ESM.pdf]

Supplementary Information

Novel Benzylidene Thiazolidinedione Derivatives as Partial PPARγ Agonists and their Antidiabetic Effects on Type 2 Diabetes

Sabina Yasmin[1], Fabio Capone[2], Antonio Laghezza[3], Fabrizio Dal Piaz[4], Fulvio Loiodice[3], Viswanathan Vijayan[5], Velmurugan Devadasan[5], Susanta K. Mondal[6], Özlem Atlı[7], Merve Baysal[7], Ashok K. Pattnaik[1], Venkatesan Jayaprakash[1*], Antonio Lavecchia[2*]

[1]Department of Pharmaceutical Sciences & Technology, Birla Institute of Technology, Mesra, Ranchi, Jharkhand 835 215, India.

[2]Department of Pharmacy, "Drug Discovery" Laboratory, University of Napoli "Federico II", Via D. Montesano, 49, 80131 Napoli, Italy

[3]Department of Pharmacy & Drug Sciences, University of Bari "Aldo Moro", via Orabona 4, 70125 Bari, Italy.

[4]Department of Pharmacy, University of Salerno, Via Giovanni Paolo II, 132, 84084 Fisciano, Italy

[5]Centre of Advanced Study in Crystallography and Biophysics, University of Madras, Maraimalai (Guindy) Campus, Chennai, India.

[6]TCG Lifesciences Ltd, Block-EP&GP, BIPL, Tower-B, Saltlake, Sector-V, Kolkata, West Bengal, India.

[7]Department of Pharmaceutical Toxicology, Faculty of Pharmacy, Anadolu University, 26470 Eskisehir, Turkey.

*Correspondence: drvenkatesanj@gmail.com (V.J.), antonio.lavecchia@unina.it (A.L.)

Supplemental Figures and Tables

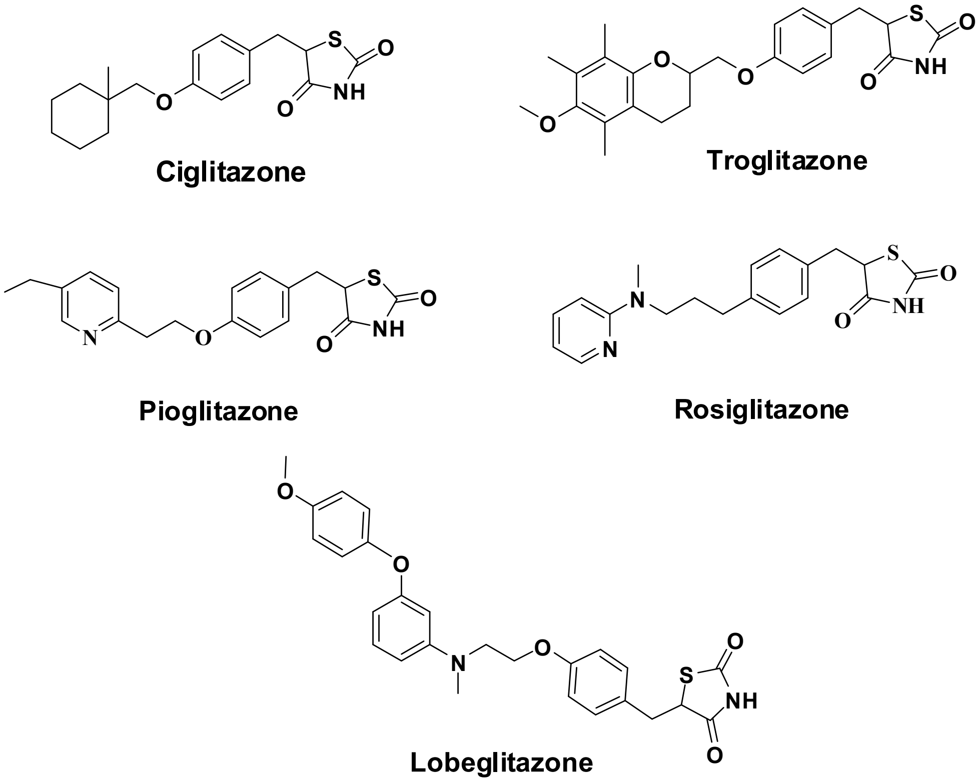

Figure S1. FDA-approved glitazones for the treatment of T2DM.

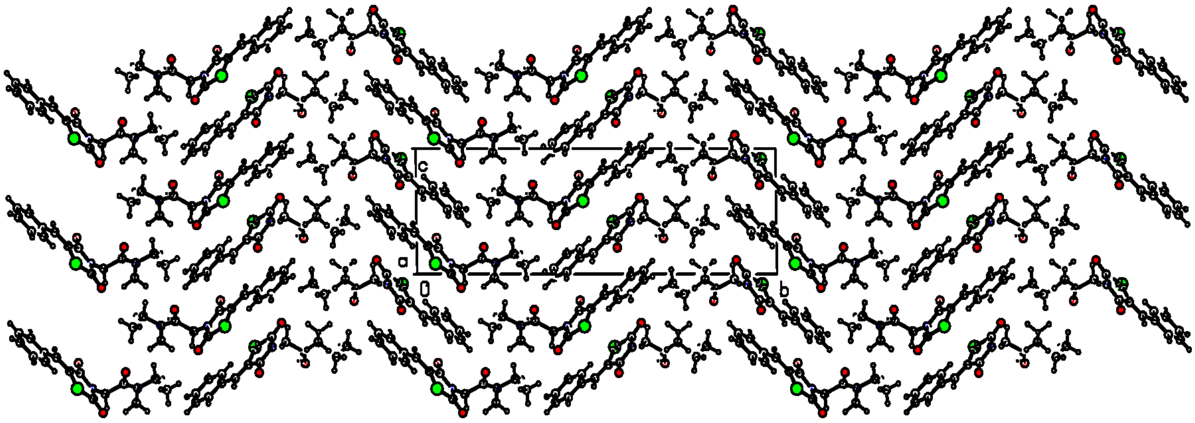

Figure S2. Molecular packing diagram of compound **1i** viewed down the "a" axis and highlighting van der Waals close contacts.

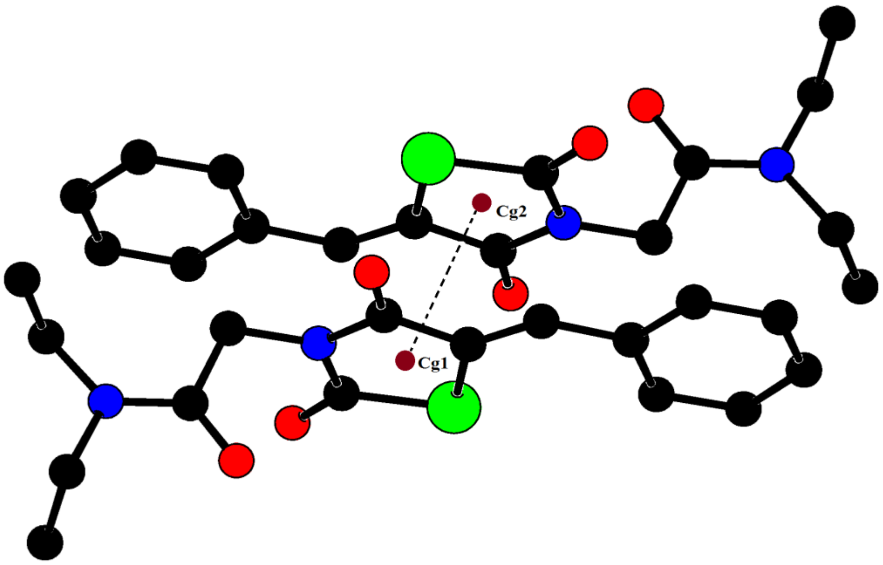

Figure S3. Part of the crystal structure of **1i** showing π-π stacking interaction (centroid-centroid distance 3.7435(13) Å (symmetry code Cg1 = x, y, z; Cg2 = 1-x, -y, 2-z).

General procedure for the preparation of the BTZD derivatives. To the stirred solution of TZD benzylidene derivatives **1-10** in acetonitrile was added a solution of appropriate acetylated amine derivatives **(a-l)** with catalytic amount of triethylamine. The reaction mixture was then refluxed for 16-20 h and cooled at room temperature. After completion of the reaction (monitored by TLC), the reaction mixture was then poured into ice-cooled water. The crude solid obtained after filtration contained the mixture of desired products, which was further purified by recrystallization with hot ethanol as solvent.

Compound **1a** (yield: 72%): ^{1}H NMR (400MHz, DMSO-d_6) : δ 8.21 (s,1H); 7.94 (s, 1H), 7.49-7.64 (m, 5 H), 4.23 (s, 2H) , 3.02 (q, *J*=8 Hz, 2H), 1.38 (q, *J*=8 Hz ,2H), 1.28 (pentate, *J*=8 Hz, 2H), 0.86 (t, *J*=7.6 Hz, 3H). ^{13}C NMR (100MHz, DMSO-d_6): δ 168.34, 167.78, 167.55, 165.76, 165.16, 133.69, 133.46, 133.34, 132.22, 131.18, 130.86, 130.159, 130.45, 129.86, 129.76, 123.97, 121.65, 43.90, 40.55, 40.34, 40.14, 39.72, 39.51, 39.30, 38.46, 31.49, 19.90, 14.07. ESI-MS (m/z): $(M+1)^+$ calcd. for $C_{16}H_{18}N_2O_3S$, 319.10 ; found , 319.10.

Compound **1b** (yield: 65%): ^{1}H NMR (400MHz, DMSO-d_6) : δ 8.34 (s, 1H); 7.95 (s, 1H), 7.48-7.65 (m, 3 H), 4.19 (s, 2H), 2.60-2.63 (m, 1H),0.59- 0.63 (m, 2H),0.37- 0.41 (m, 2H). ^{13}C NMR (100MHz, DMSO-d_6): 167.57, 166.46, 165.76, 133.79, 133.34, 131.24, 130.62, 129.89, 121.64, 43.83, 22.81, 6.01. ESI-MS (m/z): $(M+1)^+$ calcd. for $C_{15}H_{14}N_2O_3S$, 303.07 ; found , 303.1

Compound **1c** (yield: 64%): ^{1}H NMR (400MHz, DMSO-d6) : δ 8.49 (s, 1H); 7.92 (s, 1H), 7.52-7.64 (m, 6 H), 4.23 (s, 2H), 4.20 (m, 1H), 2.17 (d, *J*=8Hz, 2H), 1.88-1.92 (m, 2H), 1.62-1.67 (m, 2H). ^{13}C NMR (100MHz, DMSO-*d6*): 167.67, 167.53, 165.84, 164.25, 133.84, 133.40, 132.22, 132.15, 131.30, 130.70, 129.96, 129.19, 121.67, 67.91, 44.58, 43.90, 38.58, 30.68, 30.30, 28.88, 23.76, 22.93, 15.16, 14.44, 11.34. ESI-MS (m/z): $(M+1)^+$ calcd. for $C_{16}H_{16}N_2O_3S$, 317.09; found, 317.10.

Compound **1d** (yield: 60%): ^{1}H NMR (300MHz, CDCl3) : δ 7.96 (s, 1H),7.29-7.54 (m, 4H), 5.6 (s, 1H), 4.24 (m, 2H), 2.20 (s, 1H), 2.03-2.05 (m, 2H), 1.70-1.74 (m, 2H), 140-1.46 (m, 2H), 0.87-0.90 (t, 2H). ^{13}C NMR (100MHz, DMSO-d_6): 167.59, 165.84, 164.72, 133.94, 133.42, 131.27, 130.69, 129.94, 121.67, 79.70, 51.14, 43.96, 32.72, 23.94. ESI-MS (m/z): $(M+1)^+$ calcd. for $C_{17}H_{18}N_2O_3S$, 330.10; found , 330.2.

Compound **1e** (yield: 78%): ^{1}H NMR (400MHz, DMSO-d6) : δ 8.15 (s, 1H), 7.94 (s, 1H), 7.47-7.64 (m, 5 H), 4.22 (s, 2H), 3.48- 3.50 (m,1H), 1.50-1.72 (m,5H), 1.09-1.25 (m, 5H). ^{13}C NMR (100MHz, DMSO-d6): 167.52, 165.76, 164.23, 133.73, 133.33, 131.21, 130.61, 129.88, 121.60, 49.39, 43.91, 32.74, 25.58, 24.65. ESI-MS (m/z): $(M+1)^+$ calcd. for $C_{18}H_{20}N_2O_3S$, 345.12; found, 345.20.

Compound **1f** (yield: 69%): ^{1}H NMR (400MHz, DMSO-d6) : δ 10.40 (s,1H), 7.98 (s, 1H), 7.04-7.66 (m, 10H), 4.50 (s, 2H).^{13}C NMR (100MHz, DMSO-d6): 168.44, 167.89, 165.84, 164.30, 138.92, 134.15, 133.38, 132.30, 131. 36, 130.75, 130.54, 129.96, 124.24, 121.51, 119.67, 44.88. ESI-MS (m/z): $(M-1)^+$ calcd. for $C_{18}H_{14}N_2O_3S$, 337.07; found, 337.01.

Compound **1g** (yield: 70%): ^{1}H NMR (400MHz, DMSO-d6) : δ 8.78 (s, 1H), 7.99 (s, 1H), 7.21-7.78 (m, 10H), 4.30 (s, 2H), 4.28 (s, 2H). ^{13}C NMR (100MHz, DMSO-d6): 168.41, 167.91, 165. 81, 164.54, 137.86, 134.21, 133.55, 132.29, 131.38, 120.96, 129.97, 129.86, 127.81, 124.09, 121.12, 44.59. ESI-MS (m/z): $(M+1)^+$ calcd. for $C_{19}H_{16}N_2O_3S$, 353.10; found, 353.10.

Compound **1h** (yield: 85%): ^{1}H NMR (400MHz, DMSO-d6) : δ 8.37 (s, 1H), 7.95 (m, 1H), 7.19-7.65 (m, 10H), 4.23 (s, 2H), 3.31 (t, *J*=8Hz, 2H,), 2.71 (t, *J*=8Hz, 2H).^{13}C NMR (100MHz, DMSO-d6): 165.78, 165.42, 133.76, 133.34, 131.24, 130.61, 129.91, 129.11, 128.83, 126.63, 121.68, 40.98, 35.38. ESI-MS (m/z): $(M+1)^+$ calcd. for $C_{20}H_{18}N_2O_3S$, 367.10; found, 367.10.

Compound **1i** (yield: 72%): ^{1}H NMR (400MHz, DMSO-d6) : δ 7.95 (s, 1H), 7.40-7.78 (m, 6H), 4.5 (s, 1 H), 3.40 (q, *J*=8 Hz, 2H), 3.32 (q, *J*=8 Hz, 2H), 1.18 (t, *J*=8 Hz, 3H), 1.01 (t, *J*=8 Hz, 3H).^{13}C NMR (100MHz, DMSO-d6): 167.57, 165.77, 166.02, 133.70, 133.33, 131. 20, 130.60, 129.87, 121.64, 43.91, 40.54, 39.29, 34.13, 22.67, 15.04. ESI-MS (m/z): $(M+1)^+$ calcd. for $C_{16}H_{18}N_2O_3S$, 319.10; found, 319.1.

Compound **1j** (yield: 70%): ^{1}H NMR (400MHz, DMSO-d6) : δ 7.95 (s, 1H), 7.50-7.56 (m, 5H), 4.6 (s, 2 H), 3.61-3.63 (m, 2H), 3.53-3.57 (m, 4H), 3.41-3.43 (m, 2H). ^{13}C NMR (100MHz, DMSO-d6): 167.60, 165.82, 163.90, 134.04, 133.37, 131.33, 130.73, 129.95, 121.51, 66.45, 45.07, 43.13, 42.08. ESI-MS (m/z): $(M+1)^+$ calcd. for $C_{16}H_{16}N_2O_4S$, 333.10; found, 333.10.

Compound **1k** (yield: 72%): ^{1}H NMR (400MHz, DMSO-d6) : δ 7.96 (s, 1H), 7.49-7.67 (m, 5H), 4.5 (s, 2 H), 3.31-3.48 (m, 4H), 1.58-1.60 (m, 4H), 1.44-1.55 (m, 2H). ESI-MS (m/z): $(M+1)^+$ calcd. for $C_{17}H_{18}N_2O_3S$, 331.10; found, 331.

Compound **3a** (yield: 80%): ^{1}H NMR (400MHz, DMSO-d6) : δ 8.23 (s, 1H),7.99 (s, 1H), 7.57-7.77 (m, 4 H), 4.2 (s, 2H), 3.07 (q, *J*=8 Hz, 2H), 1.40 (q, *J*=8 Hz, 2H), 1.29 (pentate, *J*=8 Hz, 2H), 0.86 (t, *J*=8 Hz, 3H). ^{13}C NMR (100MHz, DMSO-d6): 168.11, 167.70, 167.30, 165.12, 135.43, 132.41, 132.25, 130.87, 129.95, 124.85, 119.20, 40.56, 38.86, 31.50, 19.90, 14.06. ESI-MS (m/z): $(M+1)^+$ calcd. for $C_{19}H_{16}N_2O_3S$, 353.06; found, 352.90.

Compound **6a** (yield: 70%): ^{1}H NMR (400MHz, DMSO-d6) : δ 10.38(s, 1H),
8.19 (s, 1H), 7.83 (s, 1H), 6.90-7.50 (m, 4H), 4.5 (s, 2H), 3.06 (t, *J*=8Hz, 2H), 1.39 (q, *J*=8Hz, 2H), 1.29 (pentate, *J*=8Hz, 2H), 0.86 (t, *J*=8Hz, 3H). ^{13}C NMR (100MHz, DMSO-d6): 167.83, 166.05, 165.37, 161.17, 134.27, 133.21, 124.07, 117.04, 116.80, 43.84, 31.59, 19.99, 14.17. ESI-MS (m/z): $(M+1)^+$ calcd. for $C_{16}H_{18}N_2O_4S$, 335.10; found, 334.90.

Compound **8a** (yield: 66%): ^{1}H NMR (400MHz, DMSO-d6) : δ 8.21 (s, 1H), 8.19 (s, 1H), 7.09-7.89 (m, 4 H), 4.2 (s, 2H), 3.80 (s, 2H), 3.07 (q, *J*=8 Hz, 2H), 1.40 (q, *J*=8 Hz, 2H), 1.29 (spentate, *J*=8 Hz, 2H), 0. 86 (t, *J*=8 Hz, 3H).^{13}C NMR (100MHz, DMSO-d6): 168.49, 167.97, 165.97, 161.17, 160.91, 138.99, 133.70, 132.84, 126.01, 125.90, 120.78, 118.43, 115.55, 114.70, 56.05, 55.91, 43.92, 38.93, 31.59, 30.52, 20.12, 14.16, 9.12. ESI-MS (m/z): $(M+1)^+$ calcd. for $C_{17}H_{20}N_2O_4S$, 349.11; found, 349.00.

Compound **9a** (yield: 77%): ^{1}H NMR (400MHz, DMSO-d6) : δ 8.19 (bs, 1H), 7.99 (s, 1H), 7.28-7.90 (m, 4H), 4.2 (s, 2H), 3.07 (q, *J*=8 Hz, 2H), 2.30 (s, 3H), 1.34 (q, *J*=8 Hz, 2H), 1.28 (pentate, *J*=8 Hz, 2H), 0.86 (t, *J*=8 Hz, 3H). ^{13}C NMR (100MHz, DMSO-d6): 167.68, 165.92, 165.27, 141.97, 138.12, 130.28, 119.73, 44.09, 38.44, 31.18, 21.65, 19.46, 13.43. ESI-MS (m/z): $(M+1)^+$ calcd. for $C_{17}H_{20}N_2O_3S$, 333.12; found, 332.90.

Compound **10a** (yield: 90%): ^{1}H NMR (400MHz, DMSO-d6) : δ 8.20 (s, 1H), 8.04 (s, 1H), 7.28-7.73 (m, 3H), 4.2 (s, 2H), 3.07 (q, *J*=8 Hz, 2H), 1.39 (q, *J*=8 Hz, 2H), 1.28 (pentate, *J*=8 Hz, 2H), 0.84 (t, *J*=8 Hz, 3H). ^{13}C NMR (100MHz, DMSO-d6): 166.97, 165.61, 165.26, 137.54, 135.70, 134.26, 129.72,

127.16, 118.89, 44.11, 31.73, 19.98, 14.17. ESI-MS (m/z): $(M+1)^+$ calcd. for $C_{14}H_{16}N_2O_3S_2$, 325.06; found, 325.10.

Compound **2i** (yield: 70%): ^{1}H NMR (400MHz, DMSO-d6) : δ 7.95 (m, 1H), 7.55-7.73 (m, 4H), 4.5 (s, 2 H), 3.40 (q, *J*=8 Hz, 2H), 3.28 (q, *J*=8 Hz, 2H), 1.17 (t, *J*=8 Hz, 3H), 1.01 (t, *J*=8 Hz, 3H). ^{13}C NMR (100MHz, CDCl3): 167.55, 165.93, 163.39, 135.34, 135.10, 132.49, 130.52, 130.08, 127.98, 123.41, 42.58, 41.45, 40.87, 29.79, 22.81, 14.13, 13.008. ESI-MS (m/z): $(M+1)^+$ calcd. for $C_{16}H_{17}ClN_2O_3S$, 353.06; found, 352.90.

Compound **3i** (yield: 70%): ^{1}H NMR (400MHz, DMSO-d6) : δ 7.95 (s, 1H), 7.60-7.67 (m, 4H), 4.5 (s, 2H), 3.40 (q, *J*=8 Hz, 2H), 3.27 (q, *J*=8 Hz, 2H), 1.17 (t, *J*=8 Hz, 3H), 1.01 (t, *J*=8 Hz, 3H). ^{13}C NMR (100 MHz, DMSO-d6): 168.11, 167.70, 167.30, 165.12, 135.43, 132.41, 132.25, 130.87, 129.95, 124.85, 119.20, 40.56, 38.86, 31.50, 19.90, 14.06. ESI-MS (m/z): $(M+1)^+$ calcd. for $C_{16}H_{17}ClN_2O_3S$, 353.06; found, 352.90.

Compound **4i** (yield: 75%): ^{1}H NMR (400MHz, DMSO-d6) : δ 7.96 (s, 1H), 7.37-7.73 (m, 4H), 4.5 (s, 2H), 3.34-3.40 (q, 2H), 3.22-3.27 (q, 2H), 1.17 (t, *J*=7 Hz, 3H), 1 (t, *J*=7 Hz, 3H).^{13}C NMR (100MHz, DM SO-d6): 167.37, 165.70, 164.84, 163.88, 162.23, 133.29, 133.20, 132.96, 130.04, 121.23, 117.25, 117.03, 54.26, 45.86, 43.86, 14.49, 13.41, 8.96, 8.04. ESI-MS (m/z): $(M+1)^+$ calcd. for $C_{19}H_{16}N_2O_3S$, 337.09; found, 336.9.

Compound **5i** (yield: 70%): ^{1}H NMR (400MHz, DMSO-d6) : δ 9.87 (s, 1H), 7.84 (s, 1H), 6.87-7.35 (m, 4 H), 4.5 (s, 2H), 3.38-3.40 (q, *J*=8 Hz, 2H), 3.27 (q, *J*=8 Hz, 2H), 1.17 (t, *J*=8 Hz, 3H), 1.01 (t, *J*=8 Hz, 3 H). ^{13}C NMR (100MHz, DMSO-d6): 167.70, 167.64, 165.89, 165.83, 164.40, 158.52, 158.46, 134.59, 131.03, 121.99, 121.28, 118.67, 118.63, 116.56, 14.54, 14.48, 13.45, 13.41. ESI-MS (m/z): $(M+1)^+$ calcd. for $C_{16}H_{18}N_2O_4S$, 335.10; found, 334.90.

Compound **6i** (yield: 80%): ^{1}H NMR (400MHz, DMSO-d6) : δ 10.35 (s, 1H), 7.84 (s, 1H), 6.90-7.50 (m, 4H), 4.5 (s, 2H), 3.36-3.38 (m, 2H), 3.29 (q, *J*=8 Hz, 2H),), 1.18 (t, *J*=8 Hz, 3H), 1.01(t, *J*=8 Hz, 3H). ^{13}C NMR (100MHz, CDCl3):167.62, 166.03, 163.44, 136.69, 132.76, 131.81, 129.62, 122.27, 42.55, 41.45 , 40.99, 14.23, 13.00. ESI-MS (m/z): $(M+1)^+$ calcd. for $C_{19}H_{16}N_2O_3S$, 335.10; found, 334.90.

Compound **7i** (yield: 69%): ^{1}H NMR (400MHz, DMSO-d6) : δ 7.92 (s, 1H), 7.06-7.48 (m, 4H), 4.5 (s, 2H), 3.79 (s, 3H), 3.40 (q, *J*=8 Hz, 2H), 3.28 (q, *J*=8 Hz, 2H), 1.17 (t, *J*=8 Hz, 3H), 1.01 (t, *J*=8 Hz, 3H).^{13}C NMR (100MHz, CDCl3): 168.04, 166.15, 163.56, 160.04, 134.63, 134.27, 133.85, 130.31, 122.78, 121.94, 116.63, 115.04, 55.45, 42.46, 41.45, 40.97, 14.23, 13.01. ESI-MS (m/z): $(M+1)^{+}$ calcd. for $C_{17}H_{20}N_2O_4S$, 349.11; found, 348.90.

Compound **8i** (yield: 84%): ^{1}H NMR (400MHz, DMSO-d6) : δ 7.90 (s, 1H), 7.09-7.61 (m, 4H), 4.5 (s, 2 H), 3.81 (s, 3H), 3.32-3.38 (q, *J*=8 Hz, 2H), 3.27 (q, *J*=8 Hz, 2H), 1.17 (t, *J*=8 Hz, 3H), 1.01 (t, *J*=8 Hz, 3H). ^{13}C NMR (100MHz, $CDCl_3$): 168.22, 166.38, 163.65, 161.53, 134.17, 132.32, 126.00, 118.58, 114.34, 55.57, 42.46, 41.44, 40.93, 14.23, 13.00. ESI-MS (m/z): $(M+1)^{+}$ calcd. for $C_{17}H_{20}N_2O_4S$, 349.11; found, 348.9.

Compound **9i** (yield: 84%): ^{1}H NMR (400MHz, DMSO-d6) : δ 7.91 (s, 1H), 7.34-7.54 (m, 4H), 4.5 (s, 2H), 3.40 (q, *J*=8 Hz, 2H), 3.27 (q, *J*=8 Hz, 2H), 2.35 (s, 3H), 1.17 (t, *J*=8 Hz, 3H), 1.01 (t, *J*=8 Hz, 3H). ^{13}C NMR (100MHz, CDCl3): 172.45, 170.34, 168.52, 166.56, 138.74, 137.74, 130.60, 122.98, 120.30, 60.81, 47.80, 45.90, 19.24, 18.16. ESI-MS (m/z): $(M+1)^{+}$ calcd. for $C_{17}H_{20}N_2O_3S$, 333.12; found, 333.

Compound **10i** (yield: 78%): ^{1}H NMR (400MHz, DMSO-d6) : δ 8.22 (s, 1H), 7.29-8.05 (m, 3H), 4.5 (s, 2 H), 3.37 (q, *J*=8 Hz, 2H), 3.27 (q, *J*=8 Hz, 2H), 1.17 (t, *J*=7 Hz, 3H), 1.01 (t, *J*=8 Hz, 3H).
^{13}C NMR (100MHz, DMSO-*d6*): 168.20, 167.95, 166.68, 165.92, 164.15, 138.58, 138.08, 134.80, 132.77, 129.24, 127.20, 124.92, 121.13, 118.09, 43.50, 30.82, 14.63, 12.86, 12.36. ESI-MS (m/z): $(M+1)^{+}$ calcd. for $C_{14}H_{20}N_2O_3S_2$, 325.06; found, 324.90.

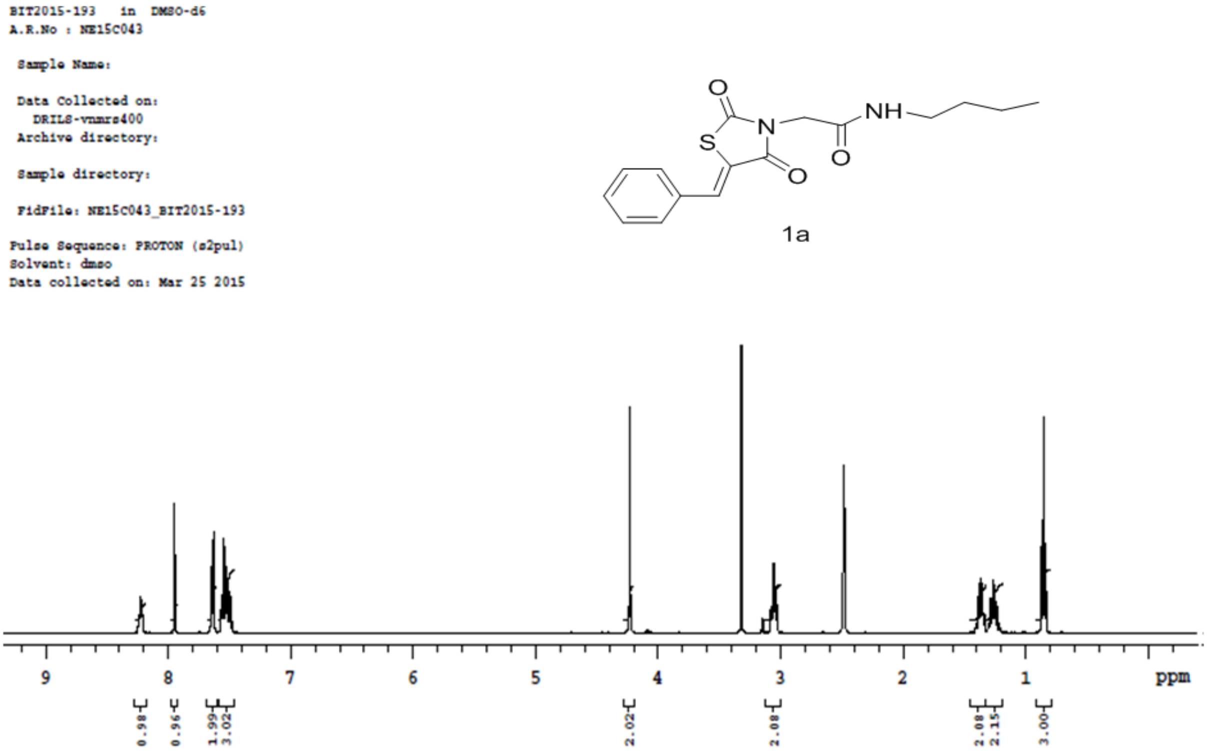

Figure S4. NMR-H spectrum of compound **1a** (DMSO-d_6, 400 MHz)

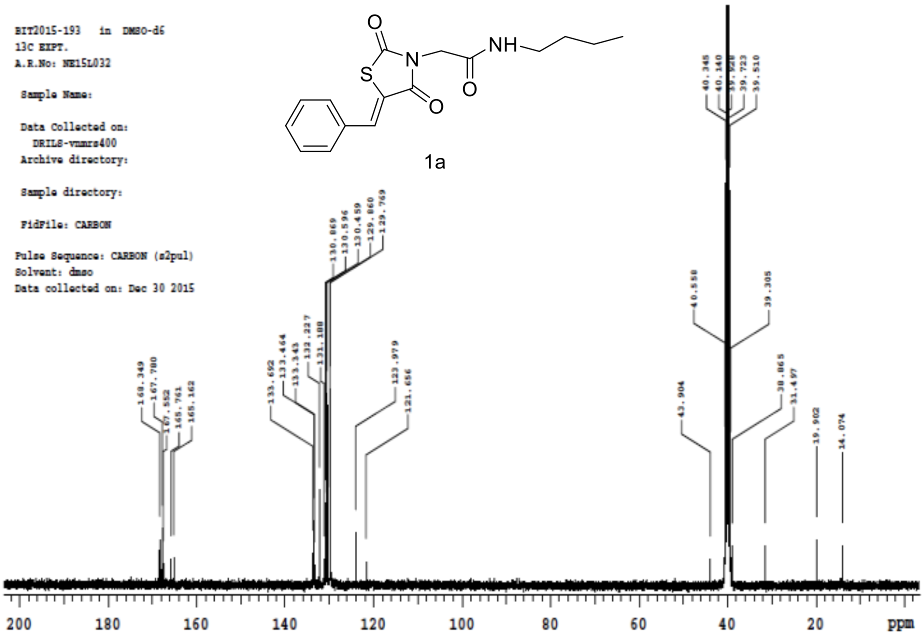

Figure S5. NMR-C^{13} spectrum of compound **1a** (DMSO-d_6, 100 MHz)

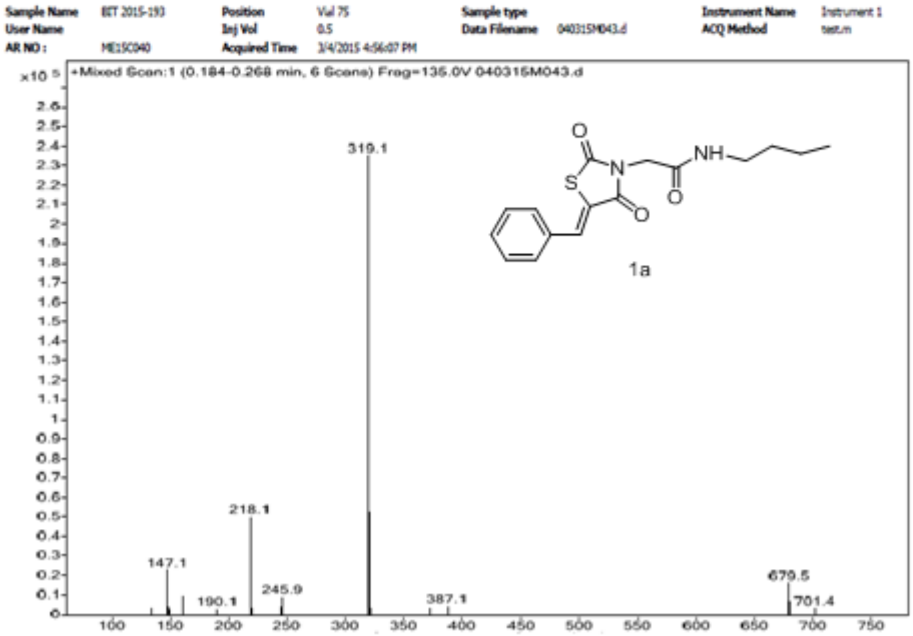

Figure S6. ESI-MS spectrum of compound **1a**

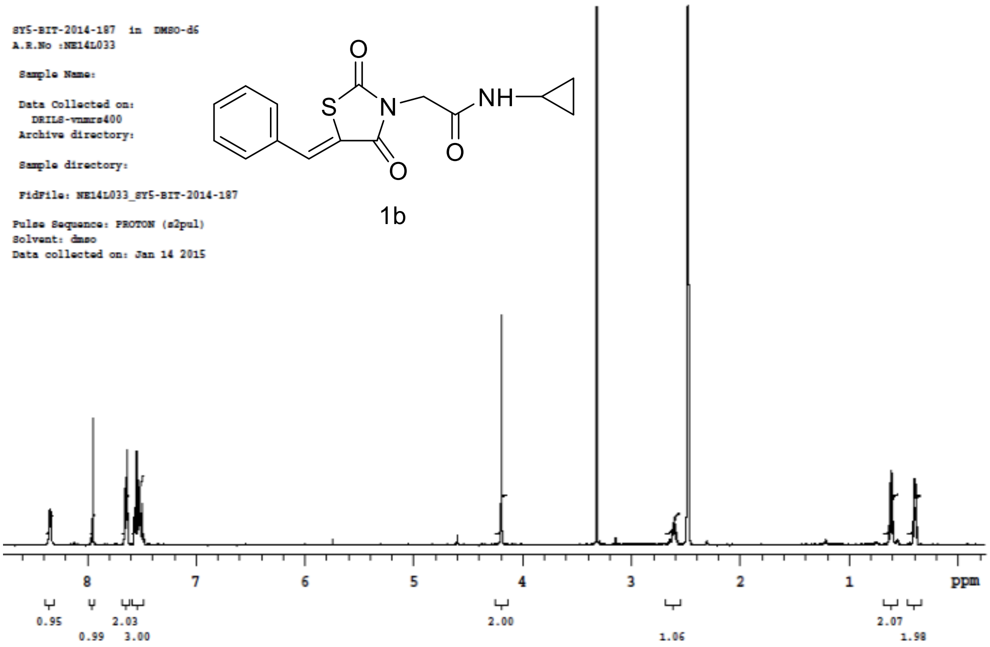

Figure S7. NMR-H spectrum of compound **1b** (DMSO-d_6, 400 MHz)

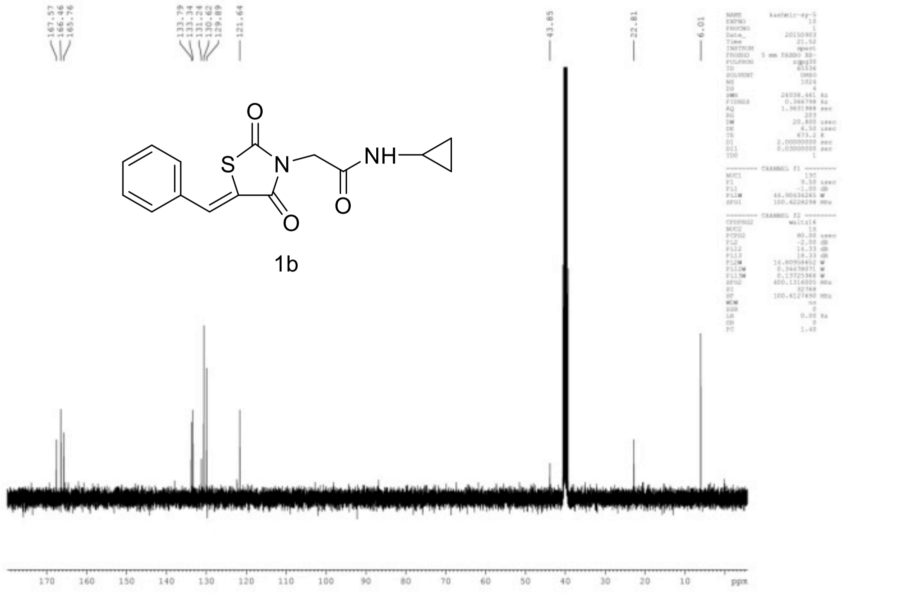

Figure S8. NMR-C[13] spectrum of compound **1b** (DMSO-d_6, 100 MHz)

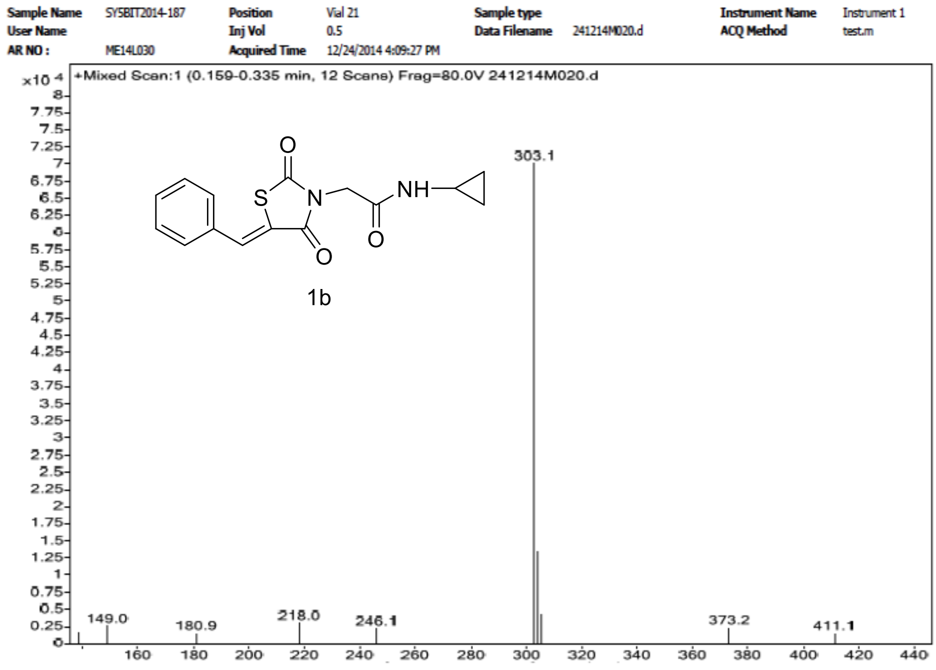

Figure S9. ESI-MS spectrum of compound **1b**

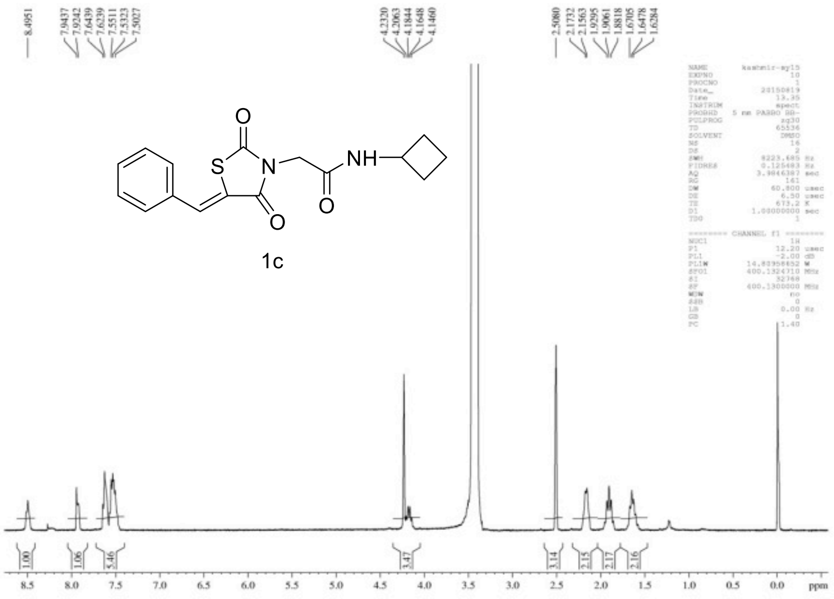

Figure S10. NMR-H spectrum of compound **1c** (DMSO-d_6, 400 MHz)

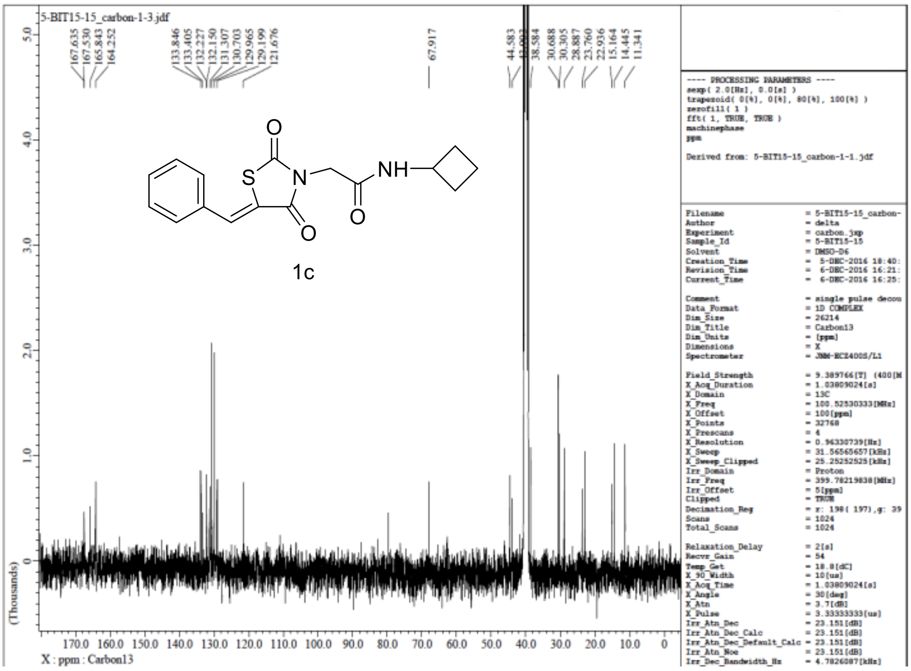

Figure S11. NMR-C[13] spectrum of compound **1c** (DMSO-d_6, 100 MHz)

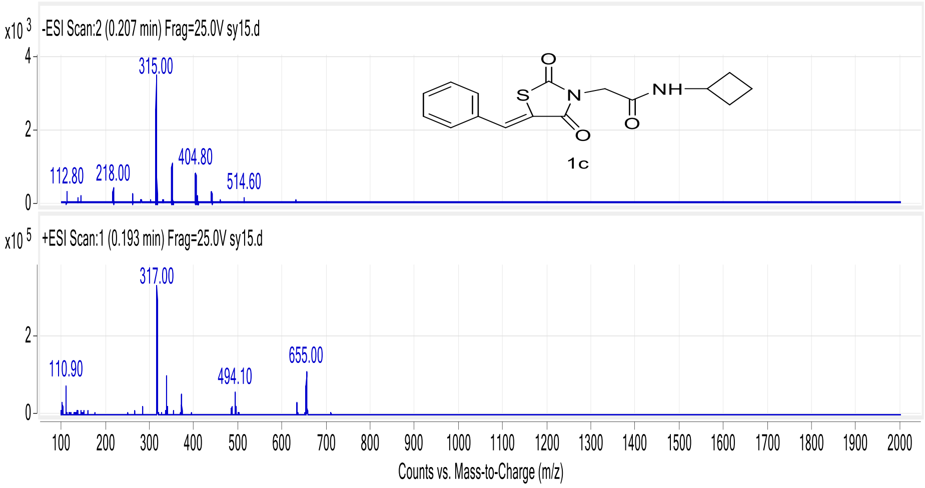

Figure S12. ESI-MS spectrum of compound **1c**

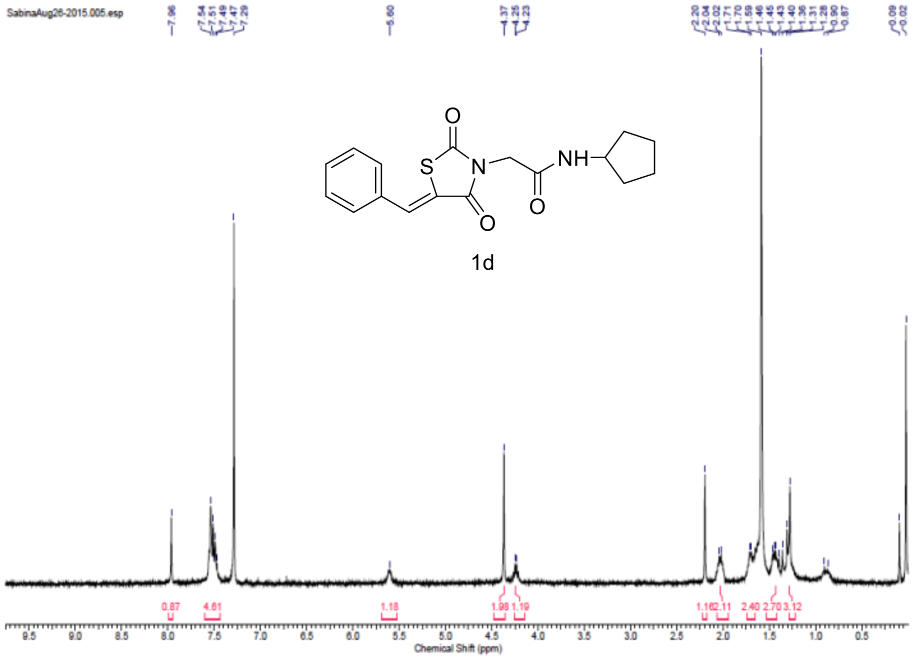

Figure S13. NMR-H spectrum of compound **1d** (300MHz, CDCl3)

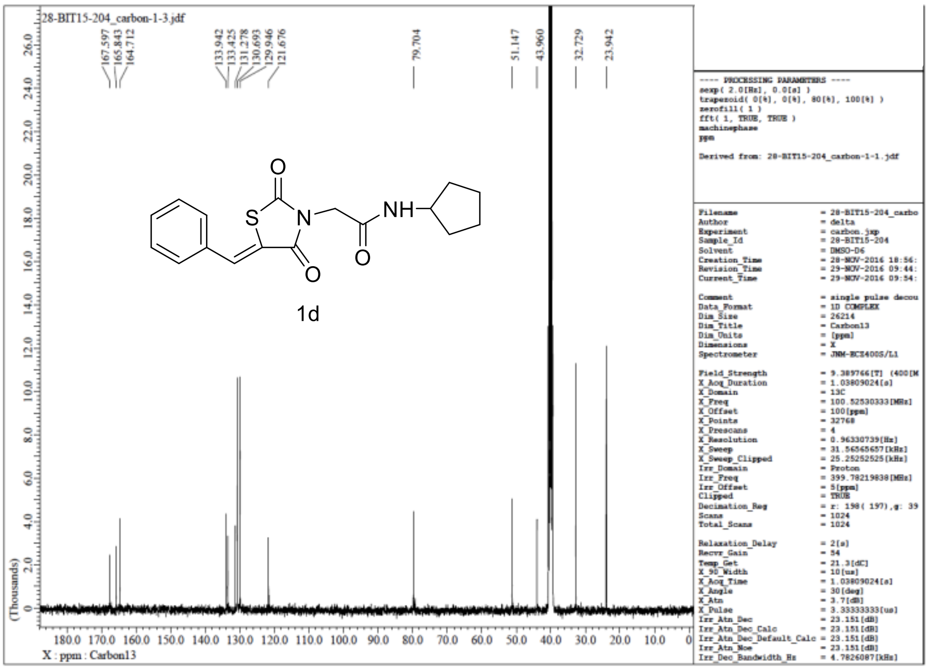

Figure S14. NMR-C[13] spectrum of compound **1d** (DMSO-d_6, 100 MHz)

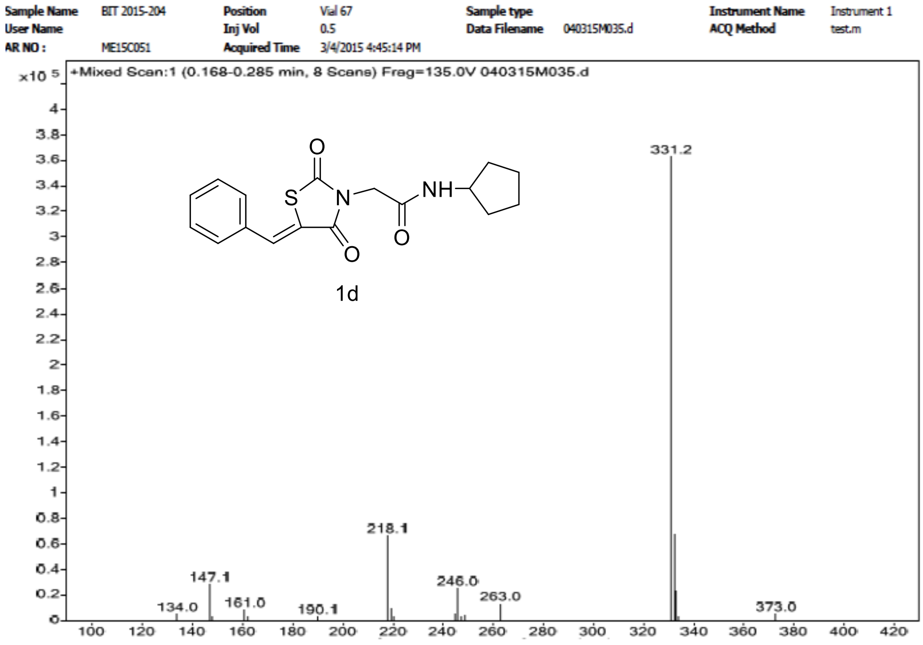

Figure S15. ESI-MS spectrum of compound **1d**

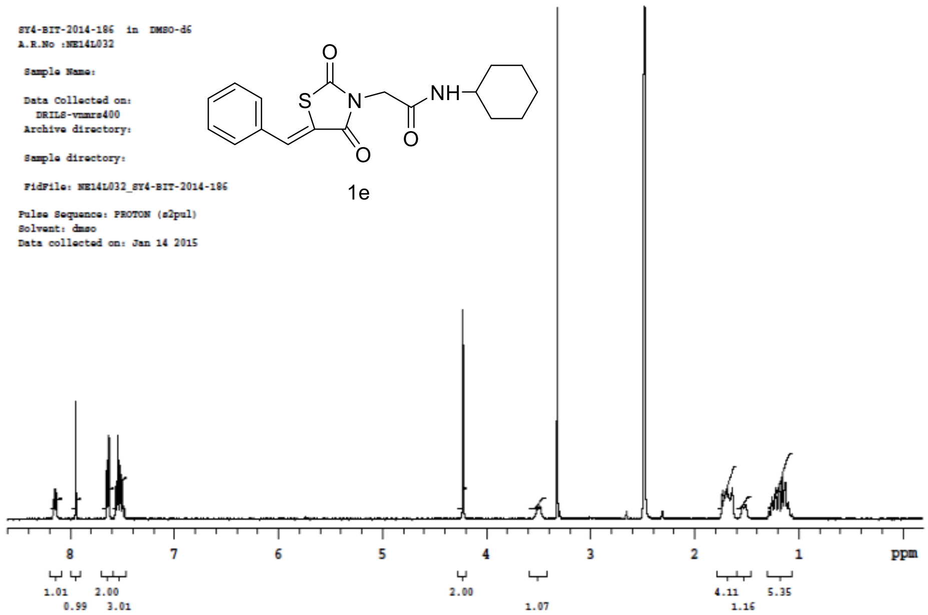

Figure S16. NMR-H spectrum of compound **1e** (DMSO-d_6, 400 MHz)

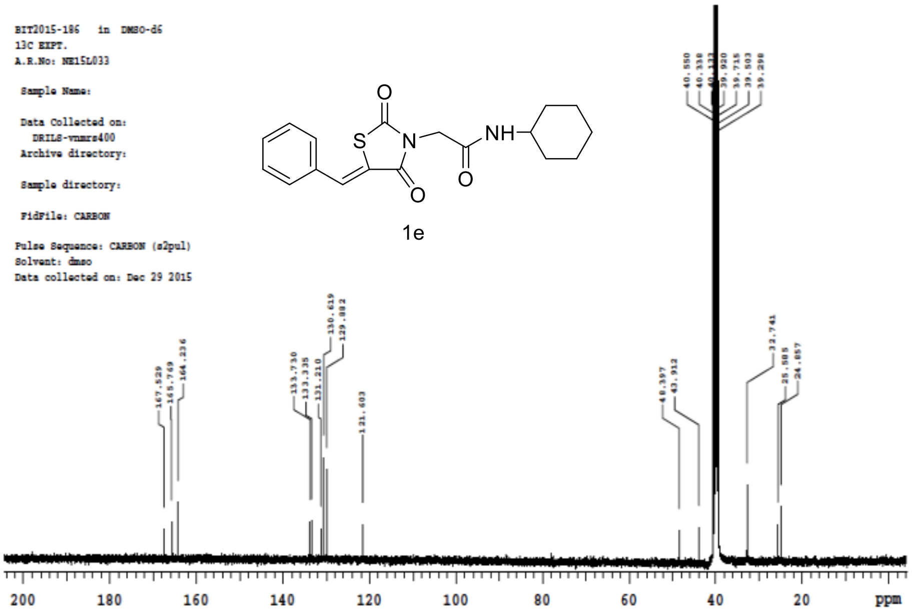

Figure S17. NMR-C[13] spectrum of compound **1e** (DMSO-d_6, 100 MHz)

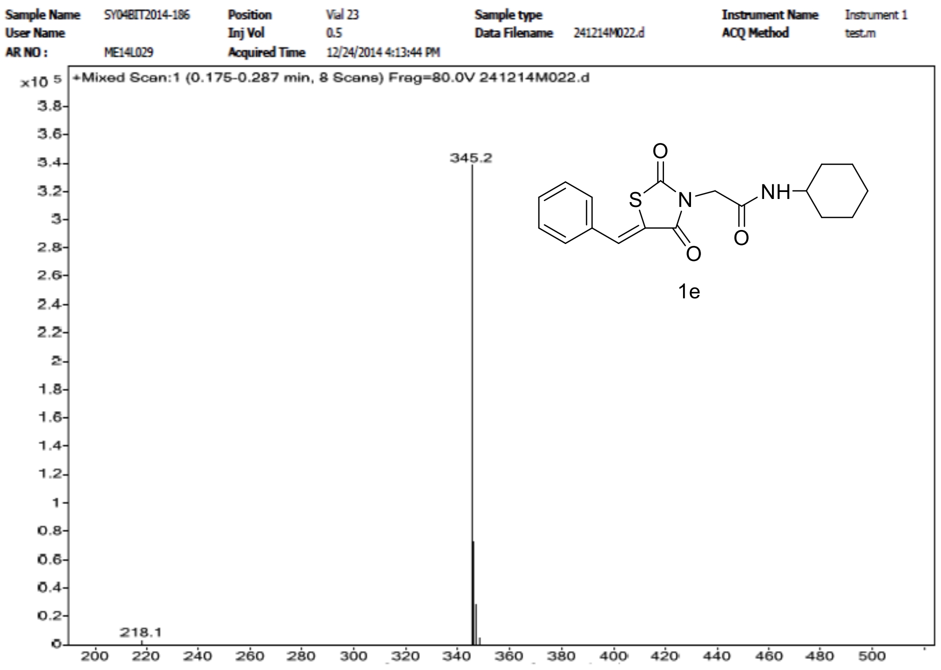

Figure S18. ESI-MS spectrum of compound **1e**

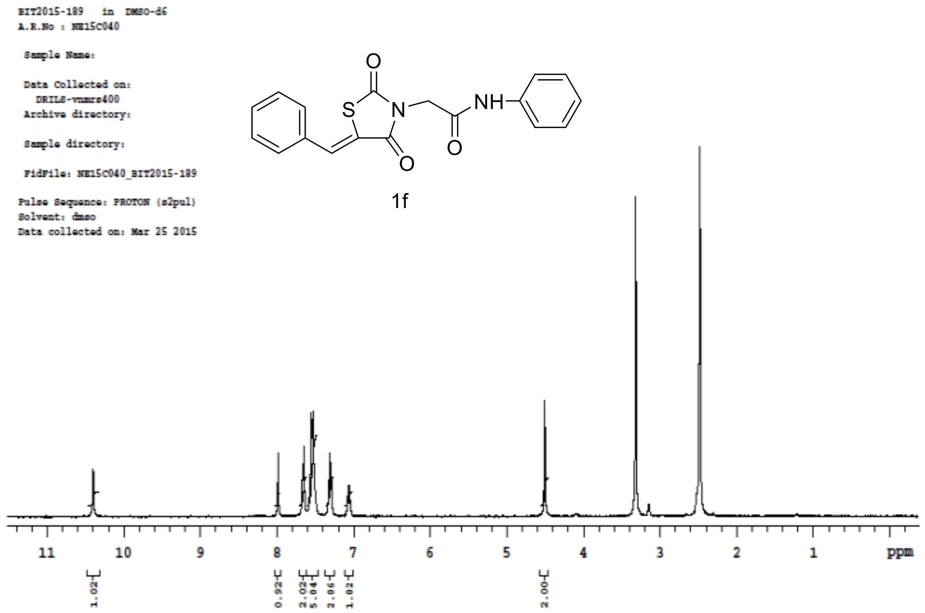

Figure S19. NMR-H spectrum of compound **1f** (DMSO-d_6, 400 MHz)

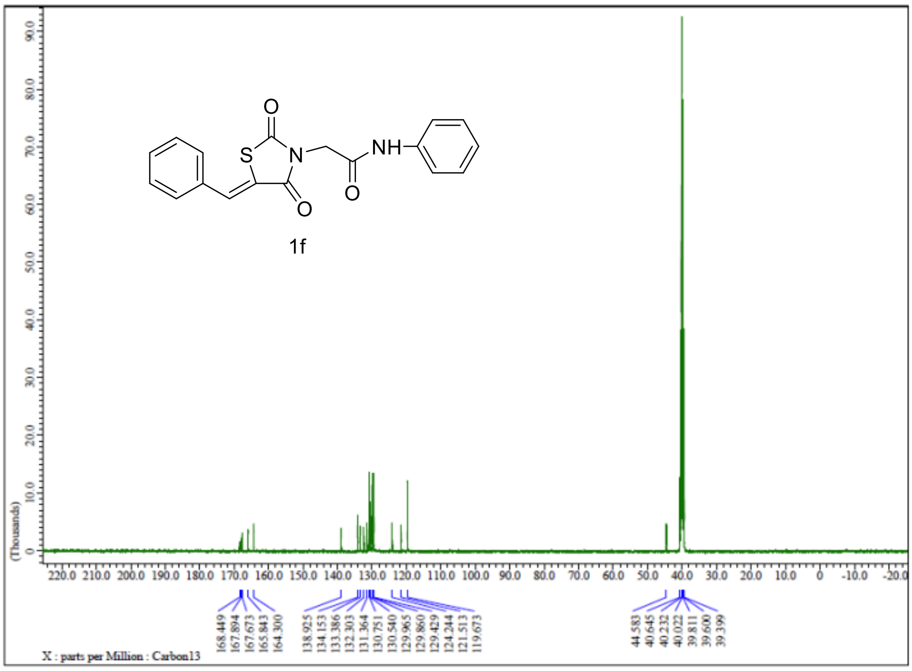

Figure S20. NMR-C[13] spectrum of compound **1f** (DMSO-d_6, 100 MHz)

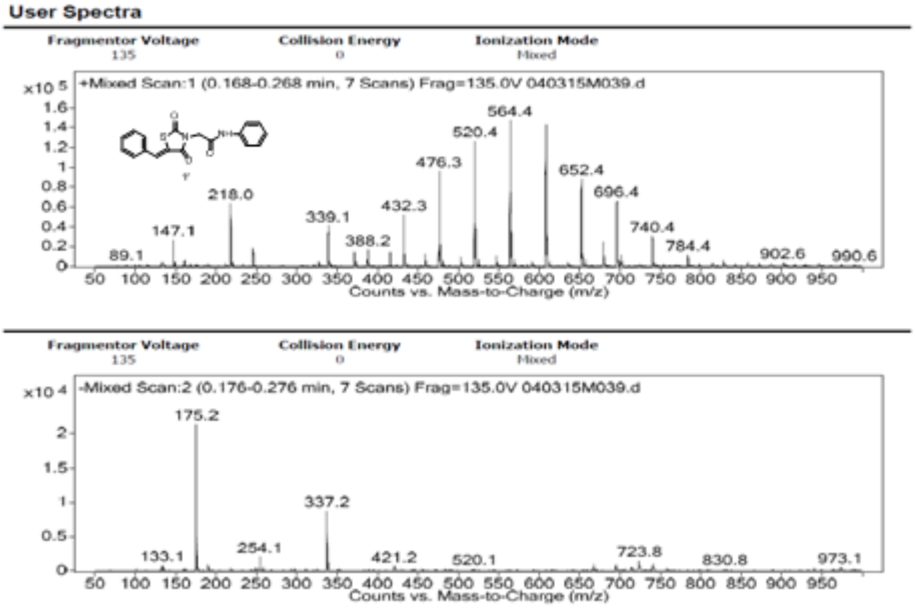

Figure S21. ESI-MS spectrum of compound **1f**

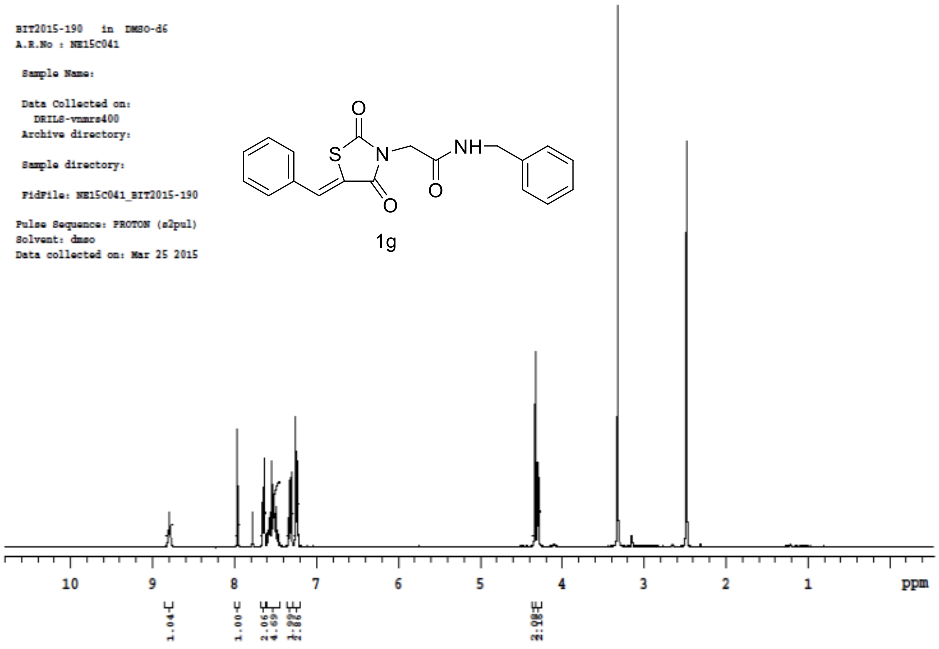

Figure S22. NMR-H spectrum of compound **1g** (DMSO-d_6, 400 MHz)

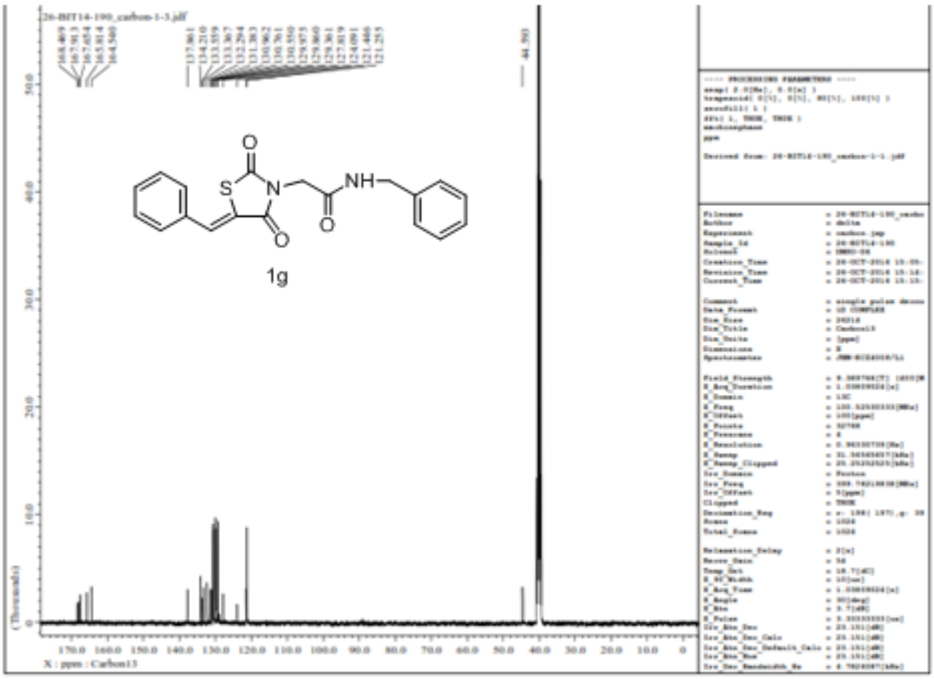

Figure S23. NMR-C^{13} spectrum of compound **1g** (DMSO-d_6, 100 MHz)

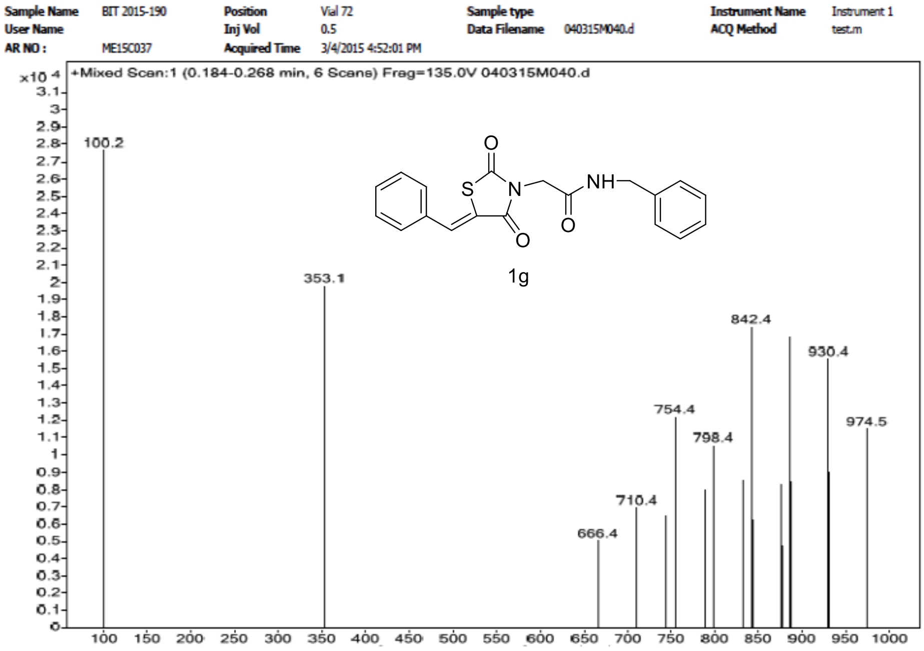

Figure S24. ESI-MS spectrum of compound **1g**

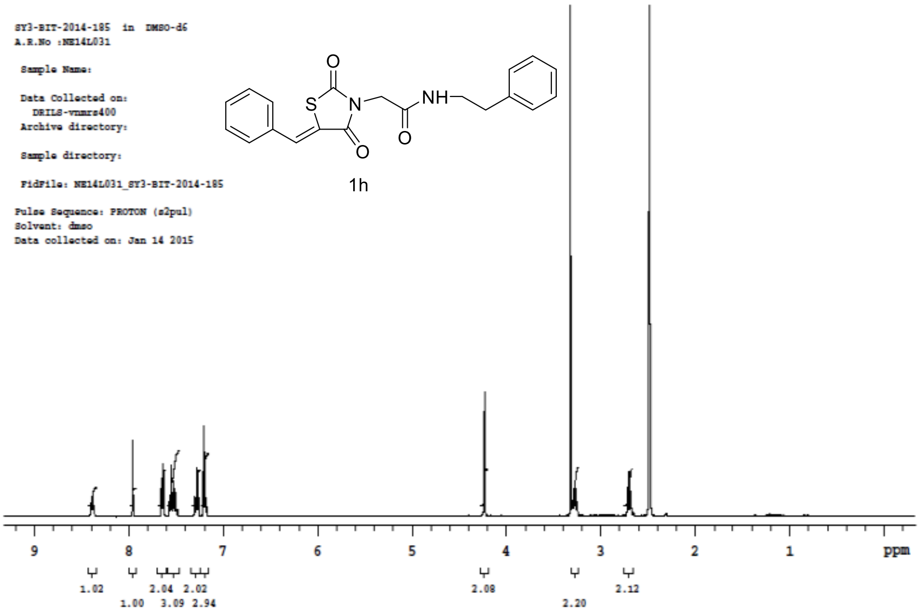

Figure S25. NMR-H spectrum of compound **1h** (DMSO-d_6, 400 MHz)

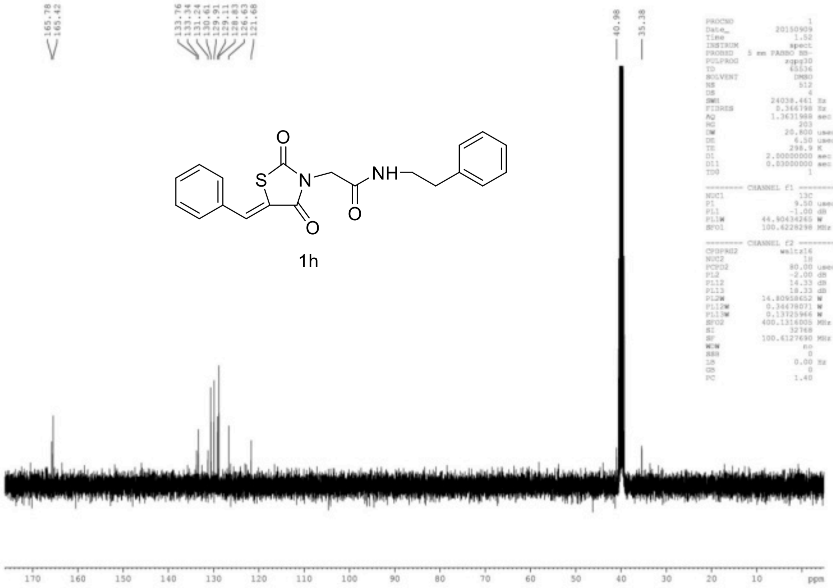

Figure S26. NMR-C[13] spectrum of compound **1h** (DMSO-d_6, 100 MHz)

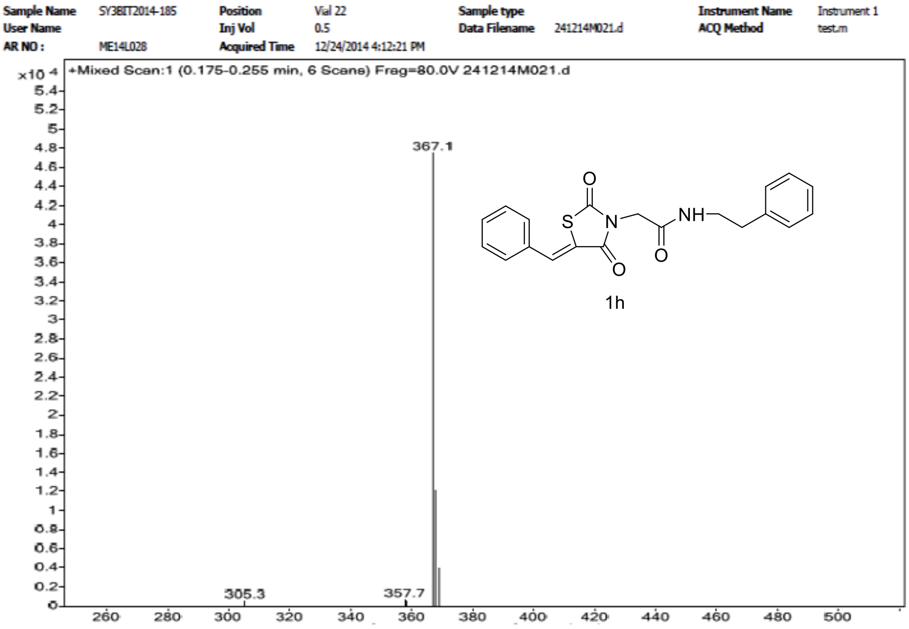

Figure S27. ESI-MS spectrum of compound **1h**

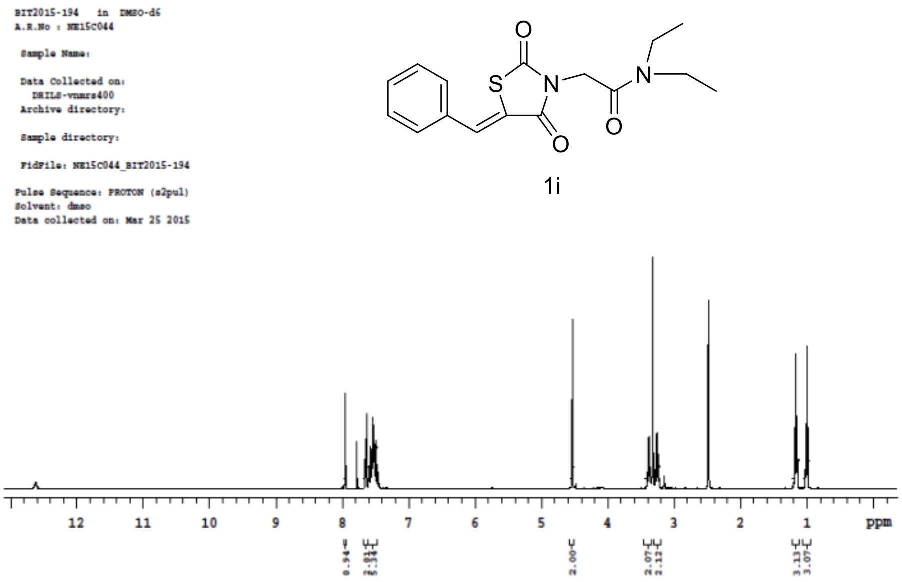

Figure S28. NMR-H spectrum of compound **1i** (DMSO-d_6, 400 MHz)

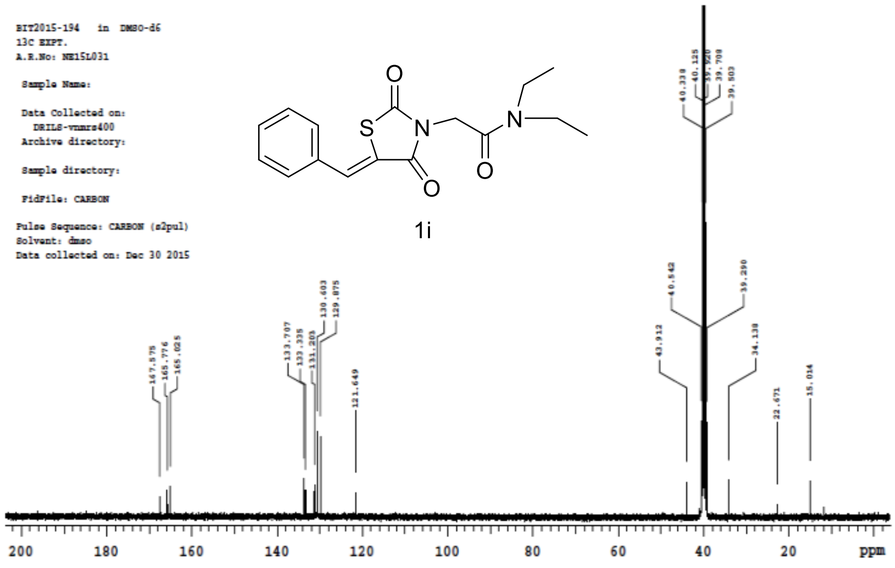

Figure S29. NMR-C^{13} spectrum of compound **1i** (DMSO-d_6, 100 MHz)

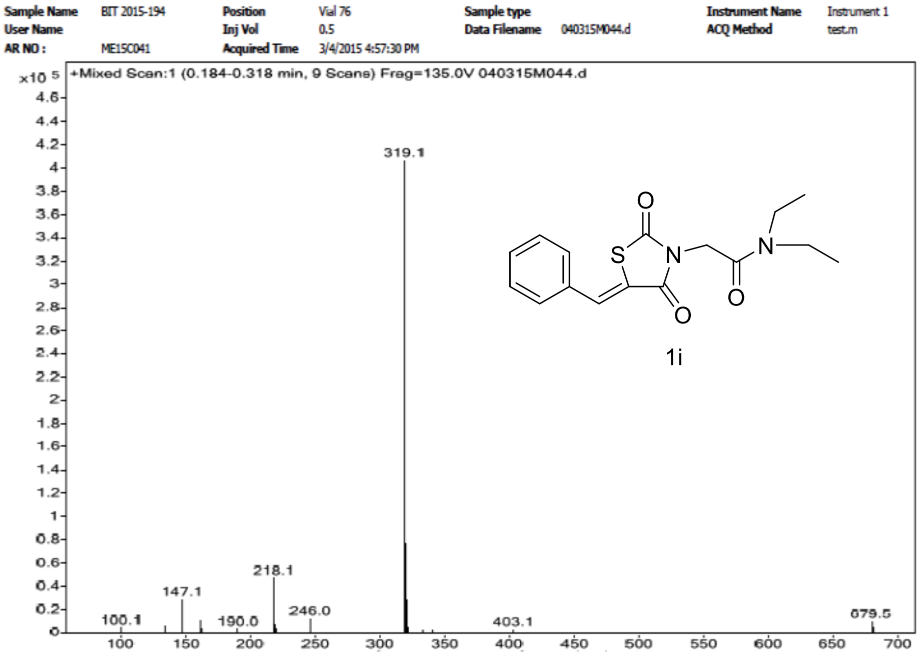

Figure S30. ESI-MS spectrum of compound **1i**

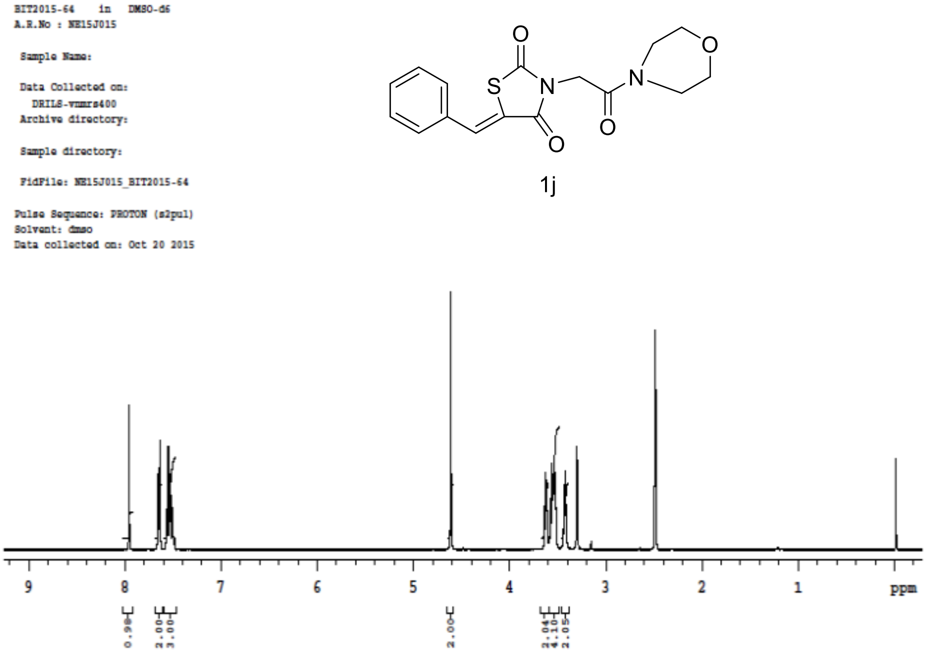

Figure S31. NMR-H spectrum of compound **1j** (DMSO-d_6, 400 MHz)

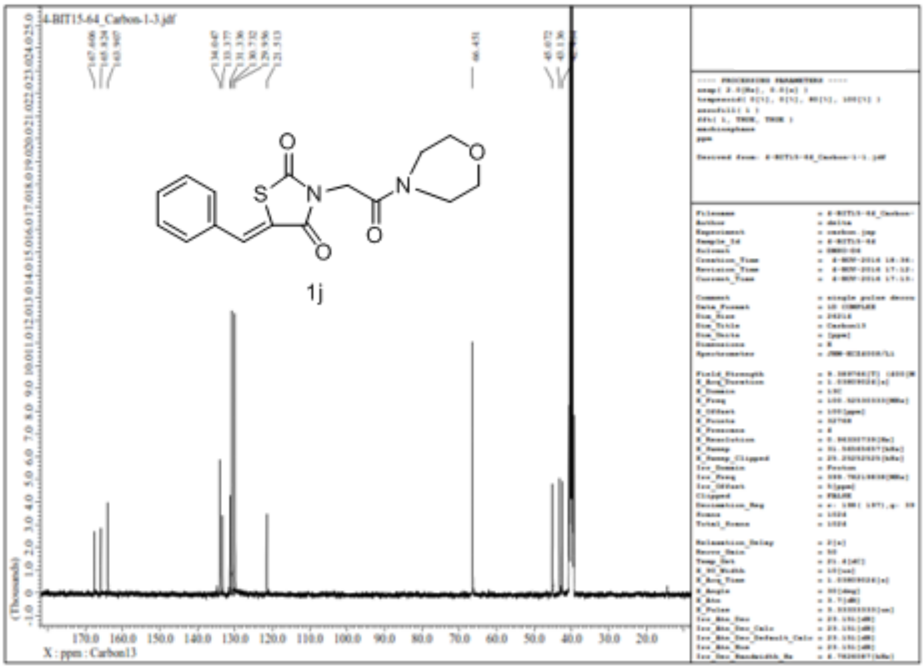

Figure S32. NMR-C^{13} spectrum of compound **1j** (DMSO-d_6, 100 MHz)

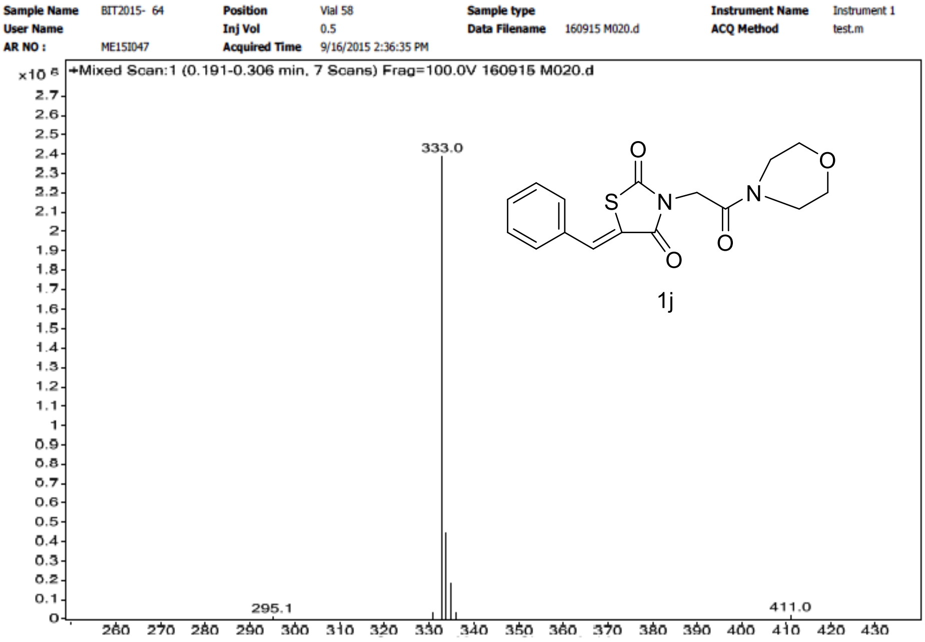

Figure S33. ESI-MS spectrum of compound **1j**

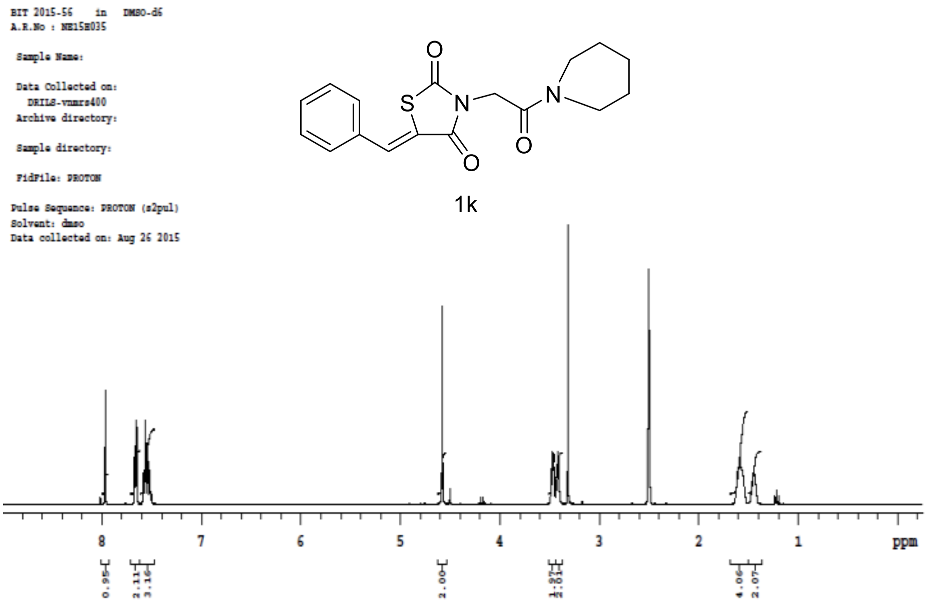

Figure S34. NMR-H spectrum of compound **1k** (DMSO-d_6, 400 MHz)

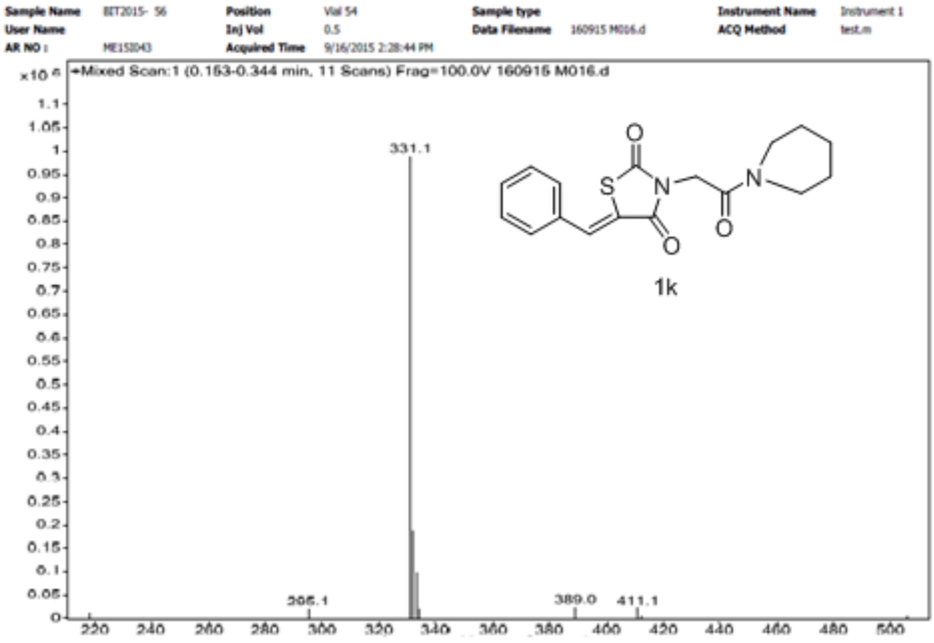

Figure S35. ESI-MS spectrum of compound **1k**

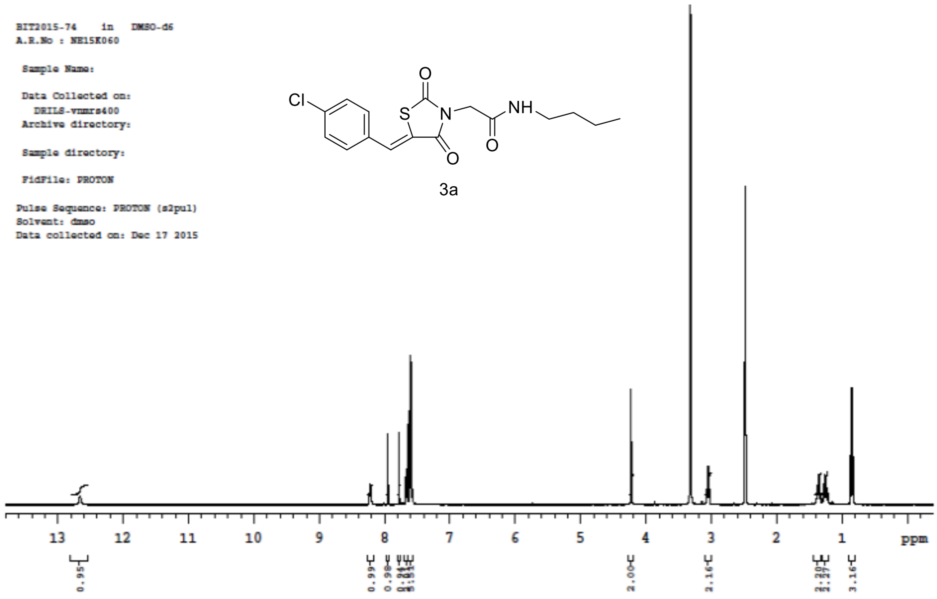

Figure S36. NMR-H spectrum of compound **3a** (DMSO-d_6, 400 MHz)

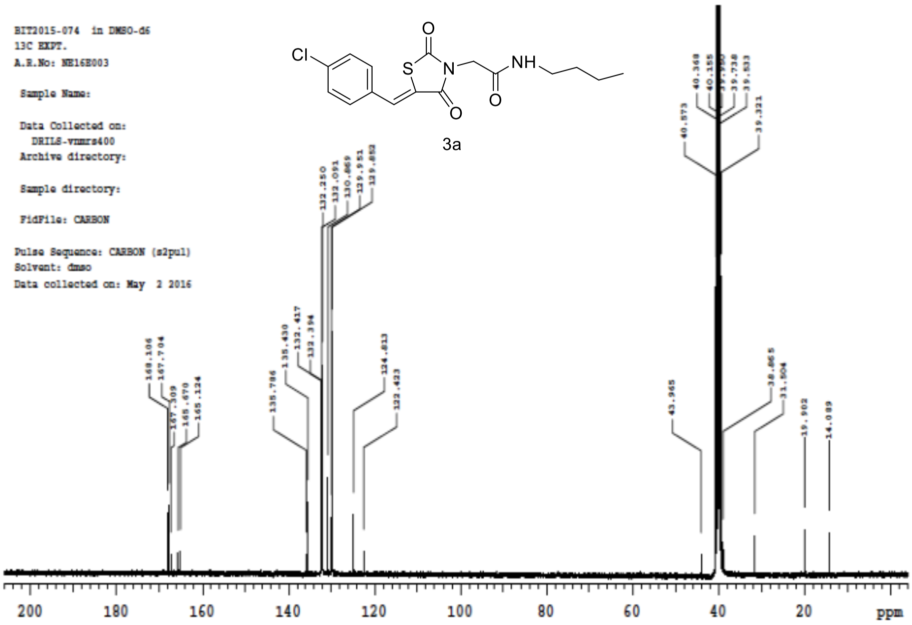

Figure S37. NMR-C[13] spectrum of compound **3a** (DMSO-d_6, 100 MHz)

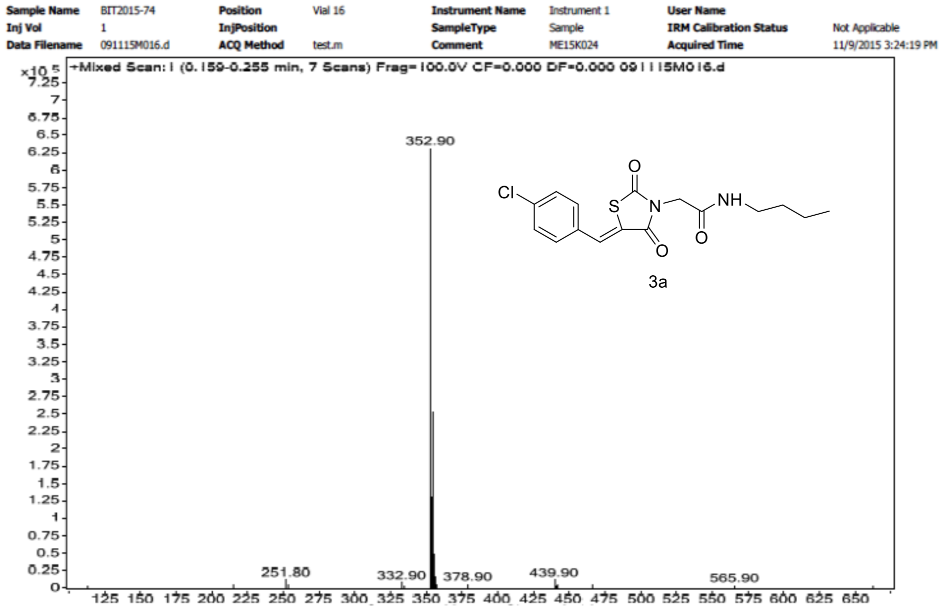

Figure S38. ESI-MS spectrum of compound **3a**

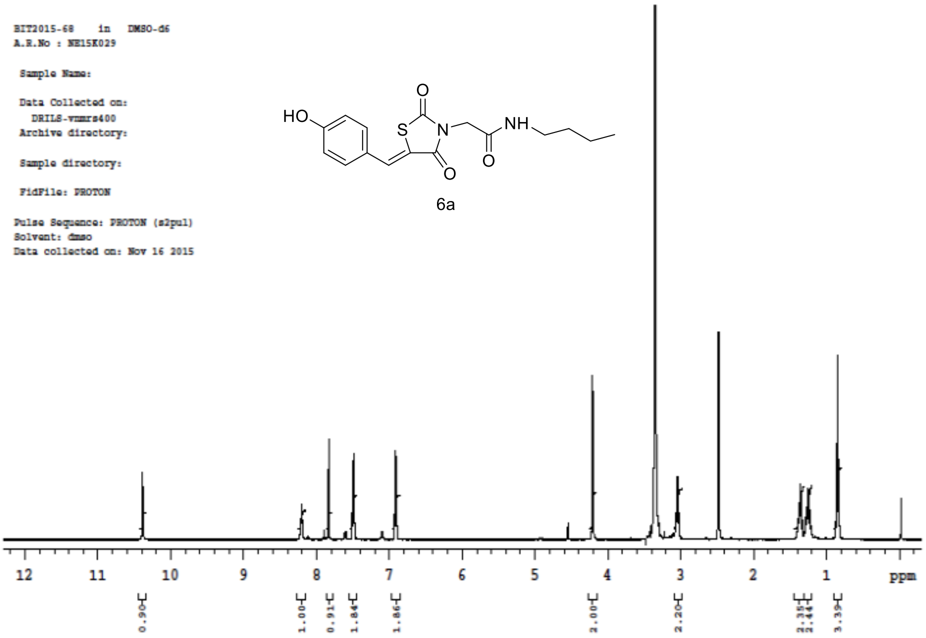

Figure S39. NMR-H spectrum of compound **6a** (DMSO-d_6, 400 MHz)

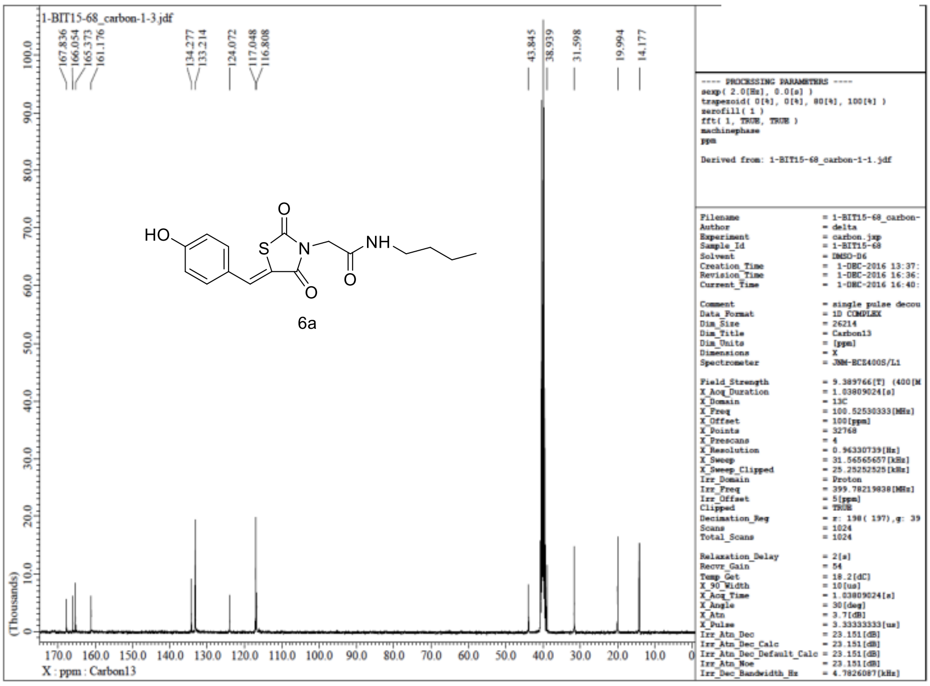

Figure S40. NMR-C[13] spectrum of compound **6a** (DMSO-d_6, 100 MHz)

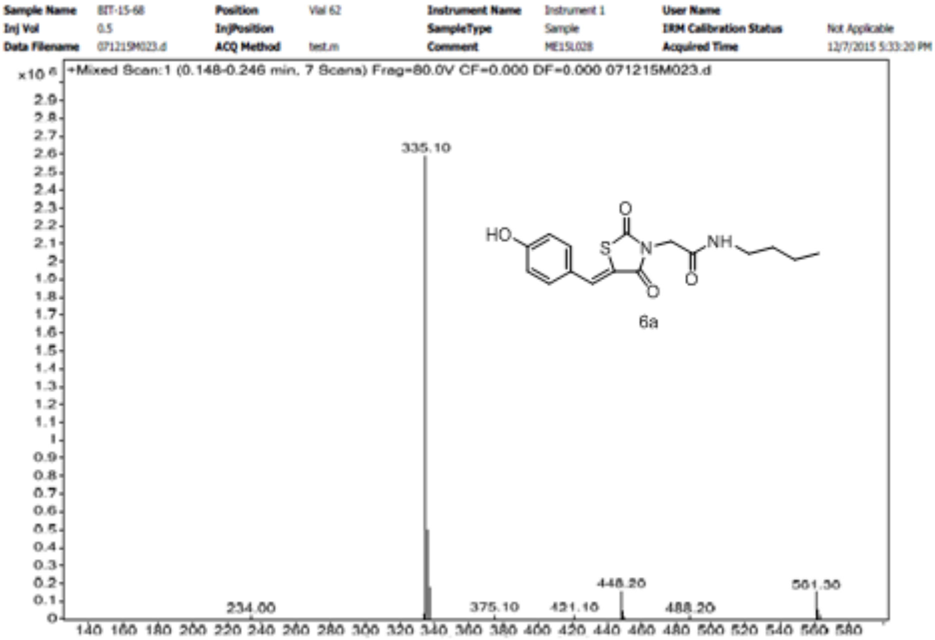

Figure S41. ESI-MS spectrum of compound **6a**

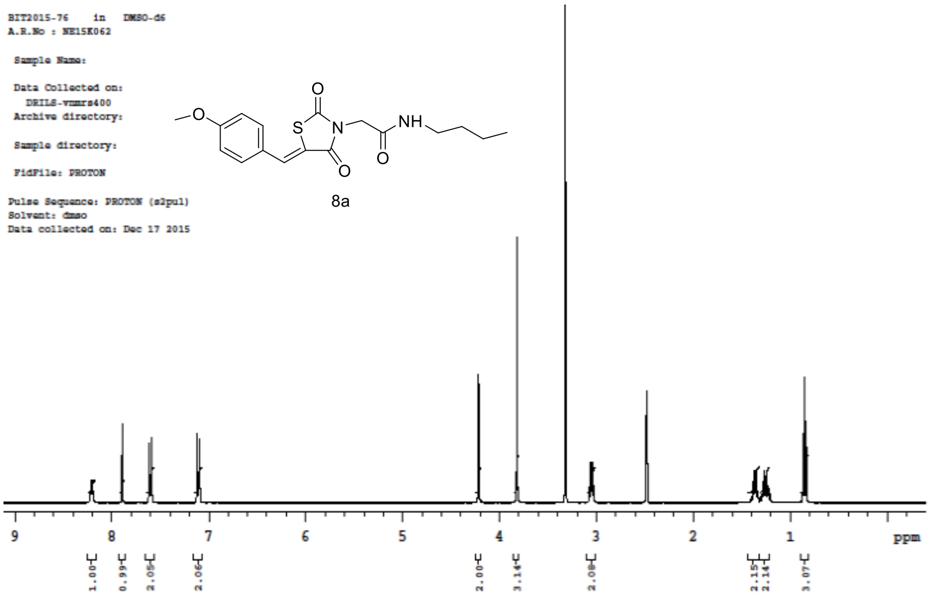

Figure S42. NMR-H spectrum of compound **8a** (DMSO-d_6, 400 MHz)

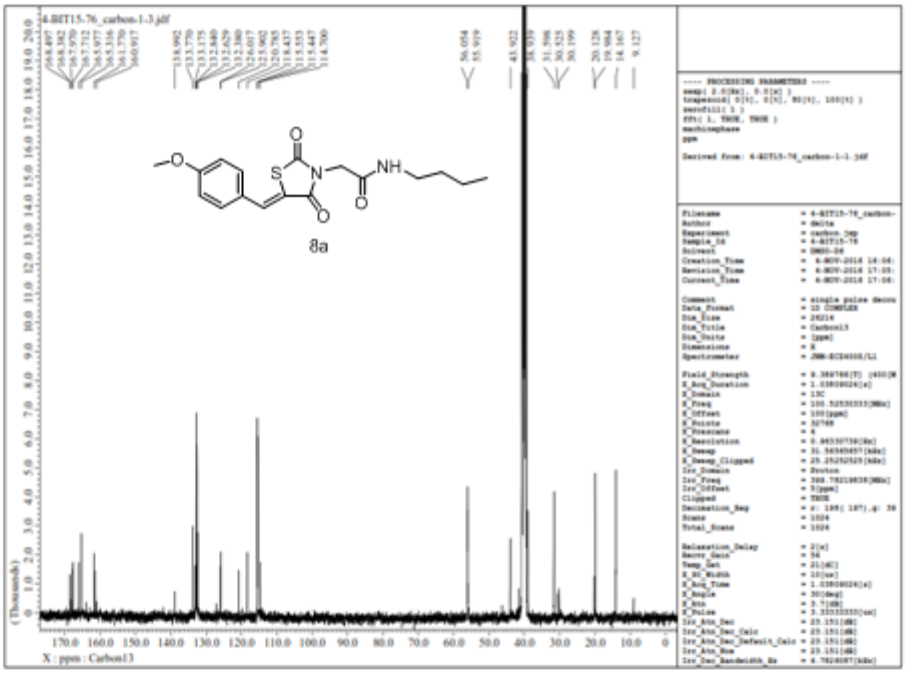

Figure S43. NMR-C[13] spectrum of compound **8a** (DMSO-d_6, 100 MHz)

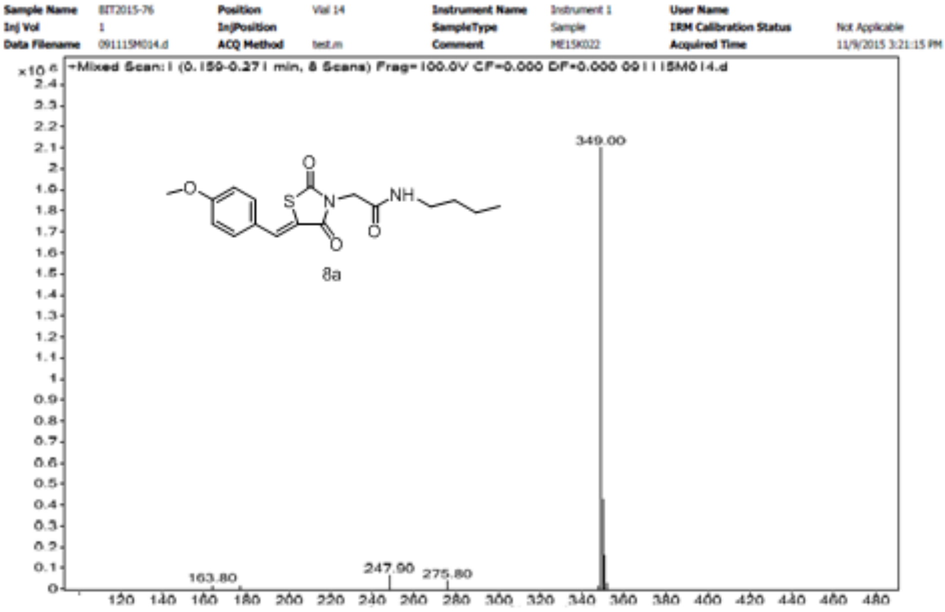

Figure S44. ESI-MS spectrum of compound **8a**

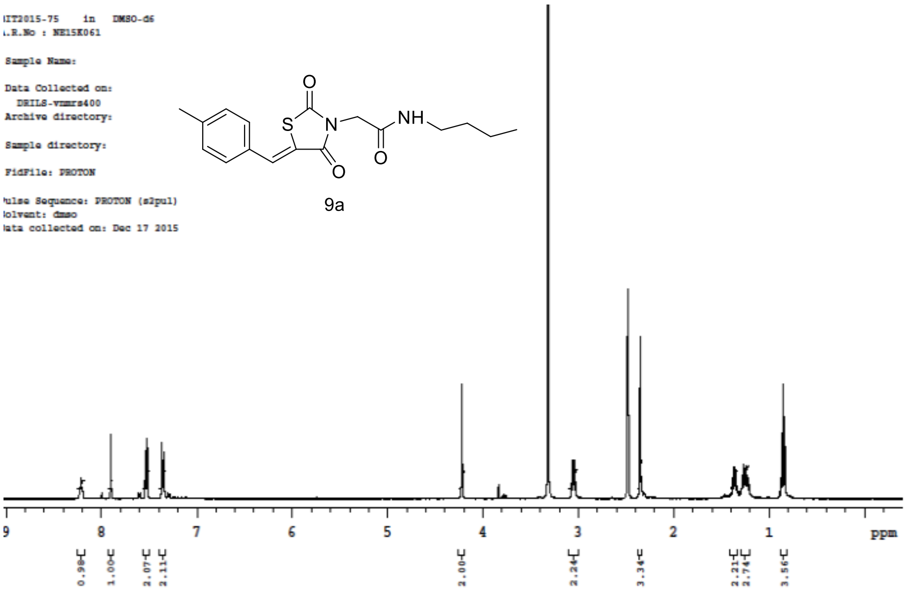

Figure S45. NMR-H spectrum of compound **9a** (DMSO-d_6, 400 MHz)

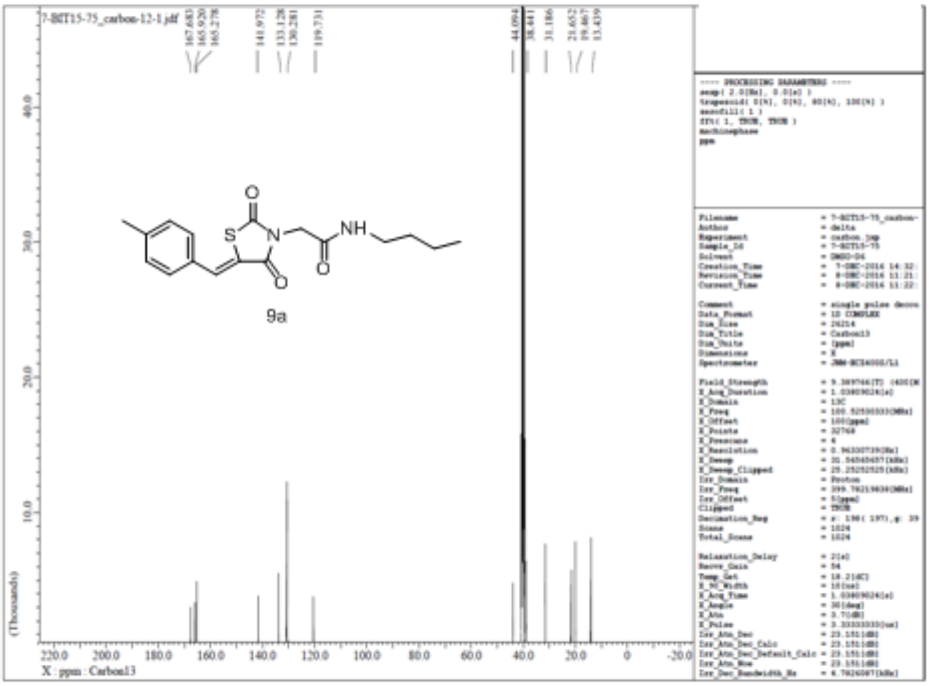

Figure S46. NMR-C[13] spectrum of compound **9a** (DMSO-d_6, 100 MHz)

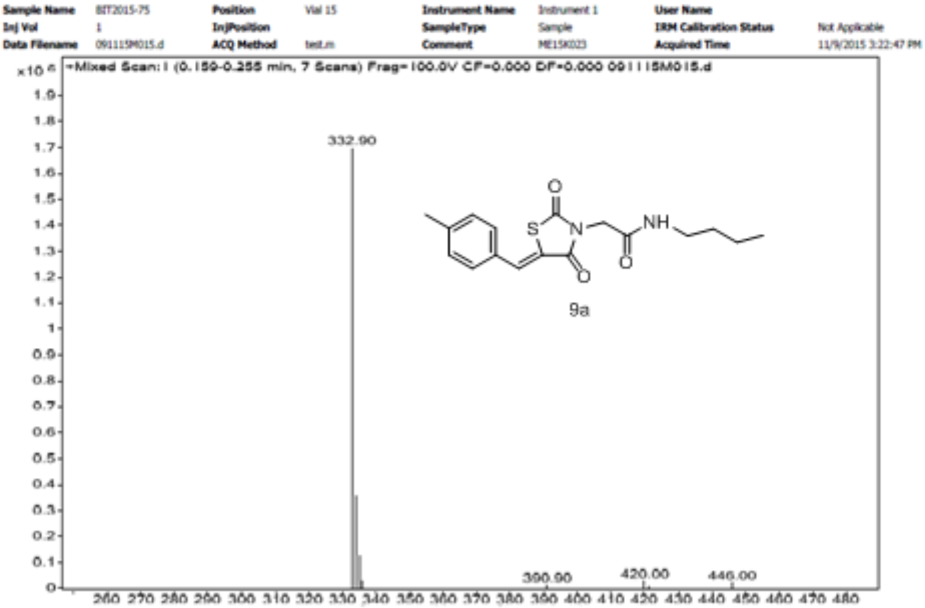

Figure S47. ESI-MS spectrum of compound **9a**

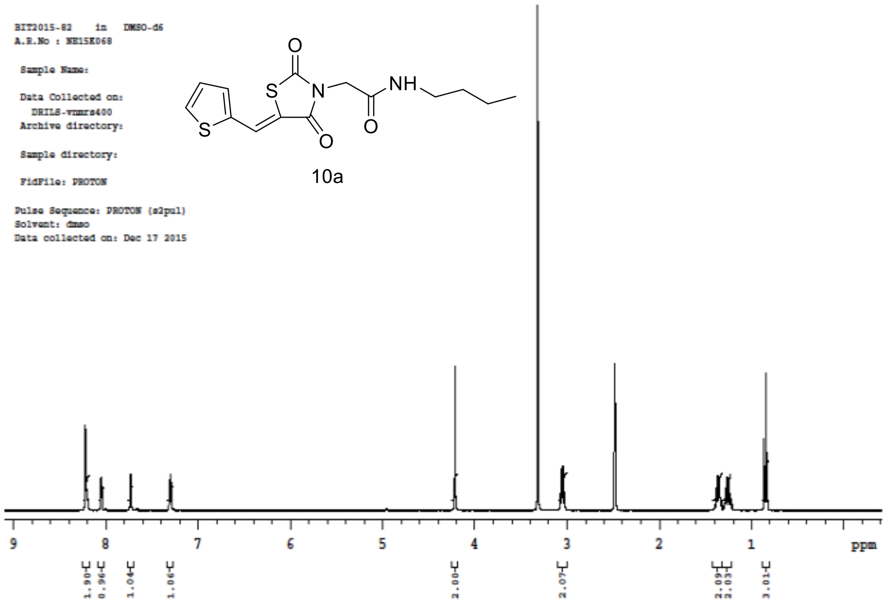

Figure S48. NMR-H spectrum of compound **10a** (DMSO-d_6, 400 MHz)

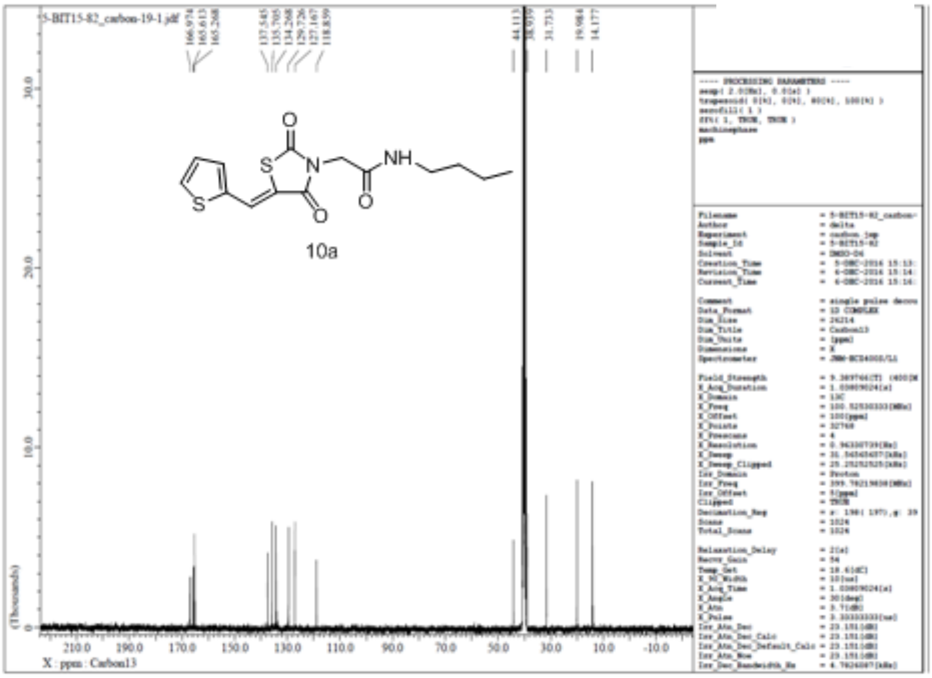

Figure S49. NMR-C[13] spectrum of compound **10a** (DMSO-d_6, 100 MHz)

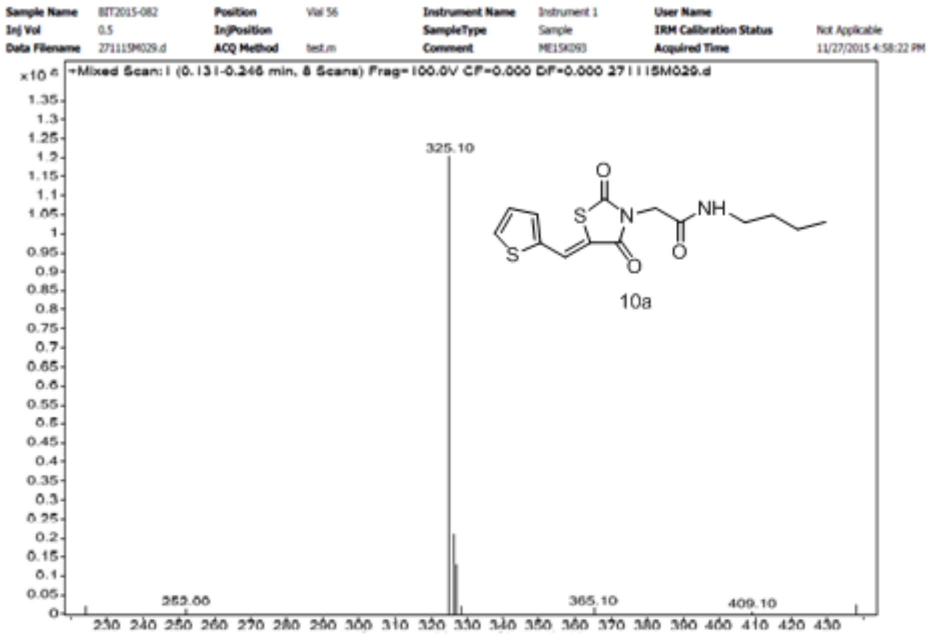

Figure S50. ESI-MS spectrum of compound **10a**

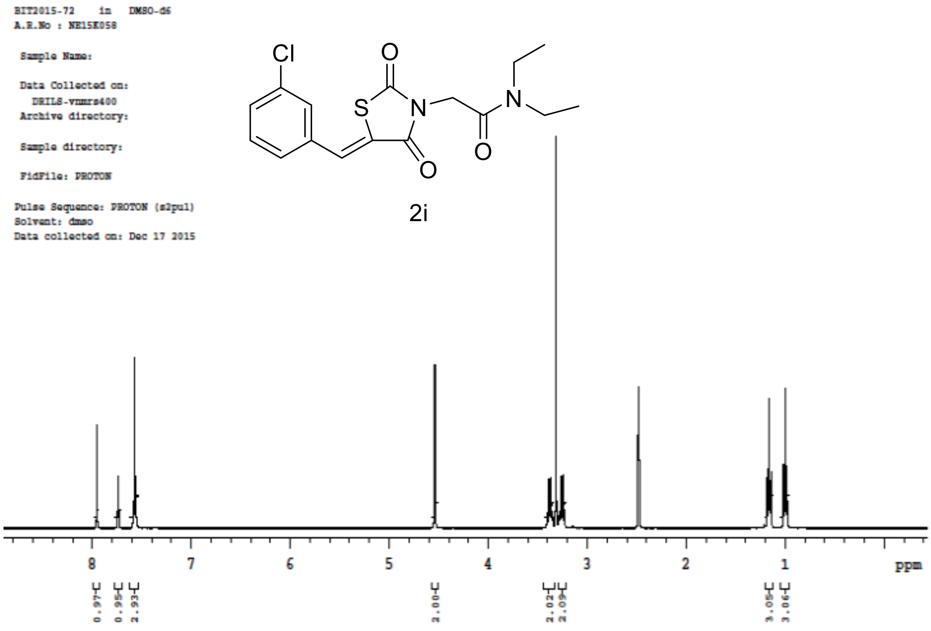

Figure S51. NMR-H spectrum of compound **2i** (DMSO-d_6, 400 MHz)

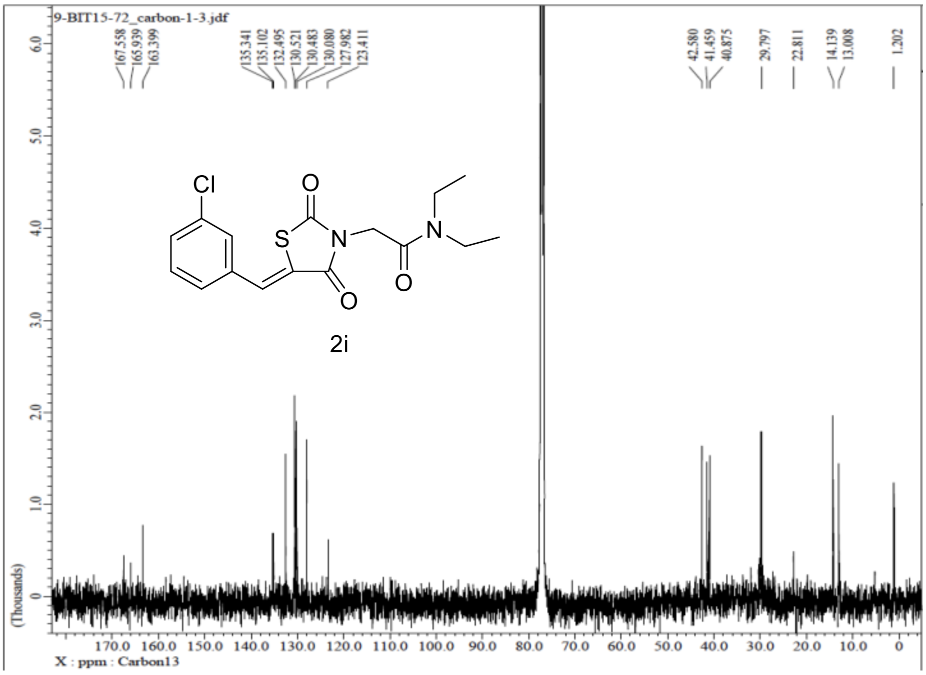

Figure S52. NMR-C^{13} spectrum of compound **2i** (100MHz, CDCl3)

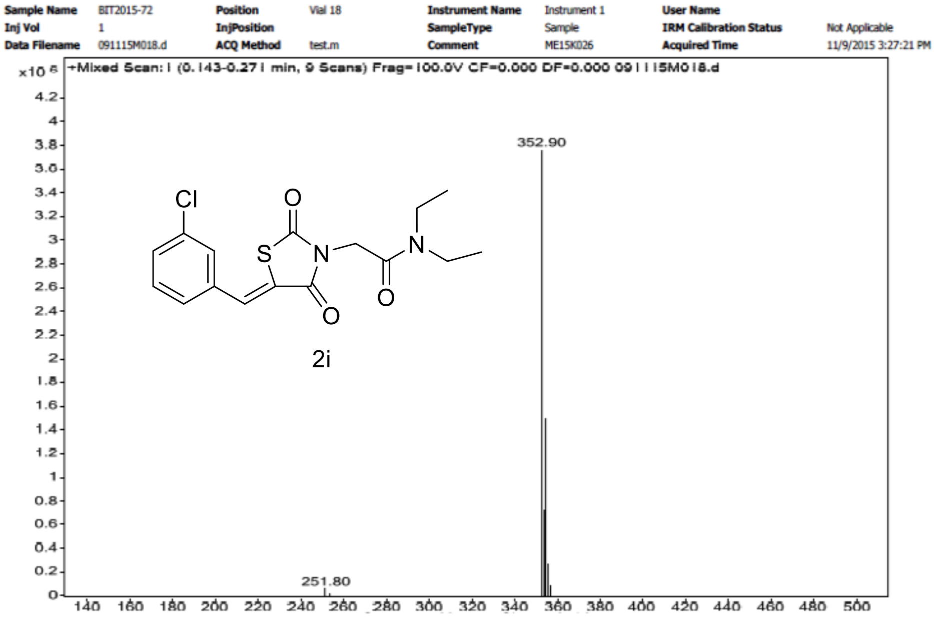

Figure S53. ESI-MS spectrum of compound **2i**

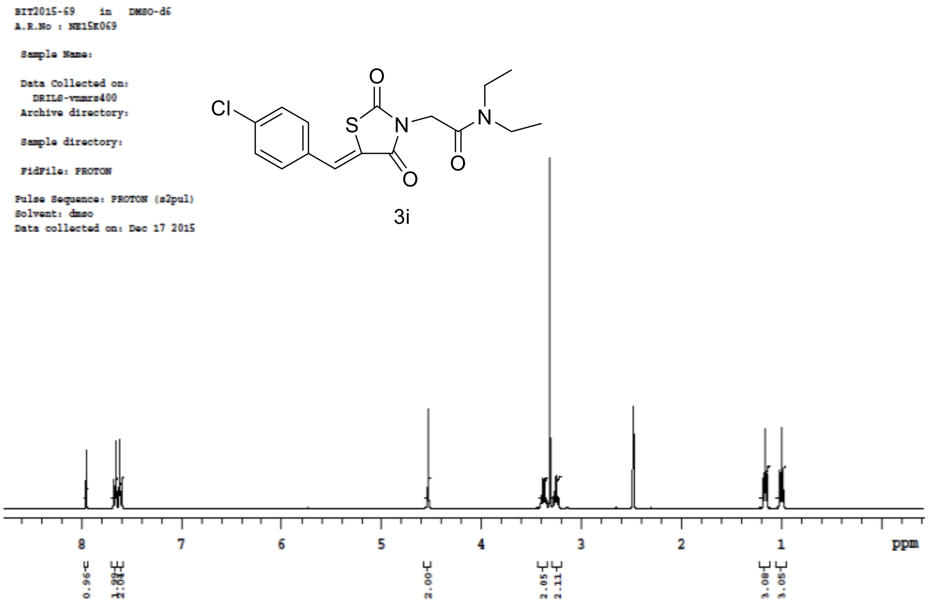

Figure S54. NMR-H spectrum of compound **3i** (DMSO-d_6, 400 MHz)

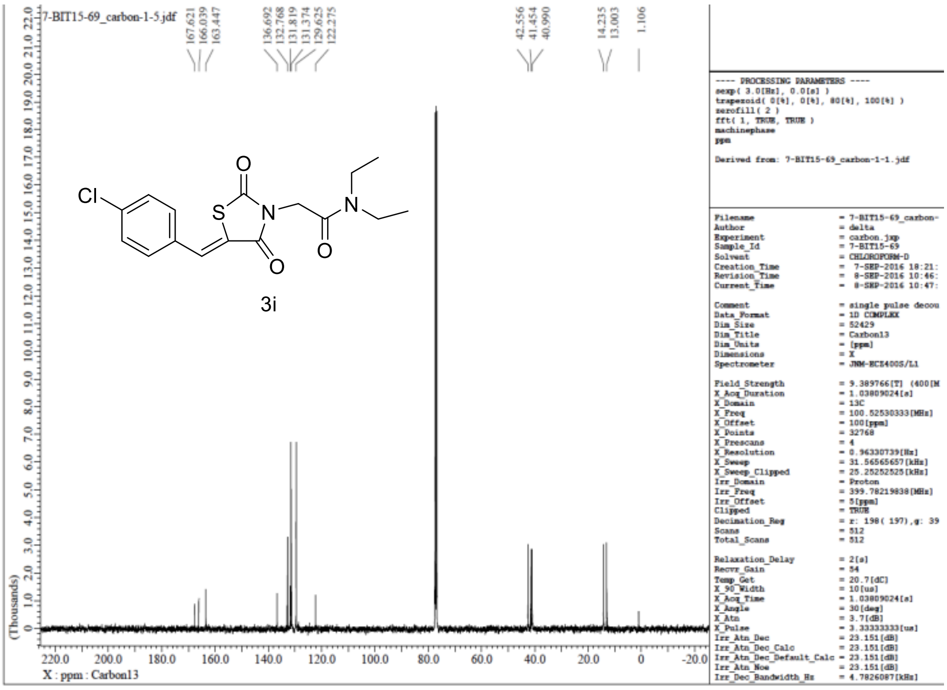

Figure S55. NMR-C[13] spectrum of compound **3i** (DMSO-d_6, 100 MHz)

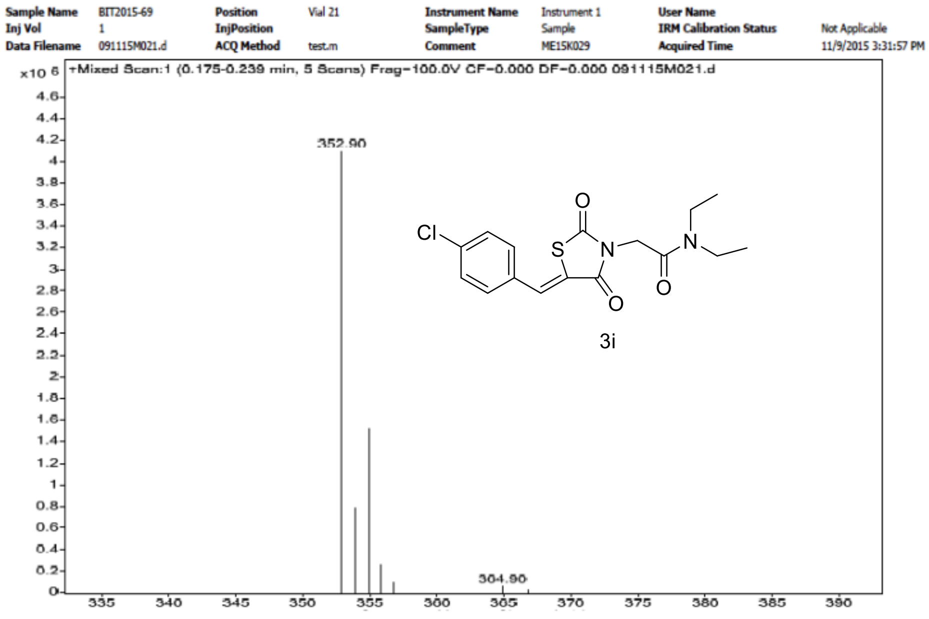

Figure S56. ESI-MS spectrum of compound **3i**

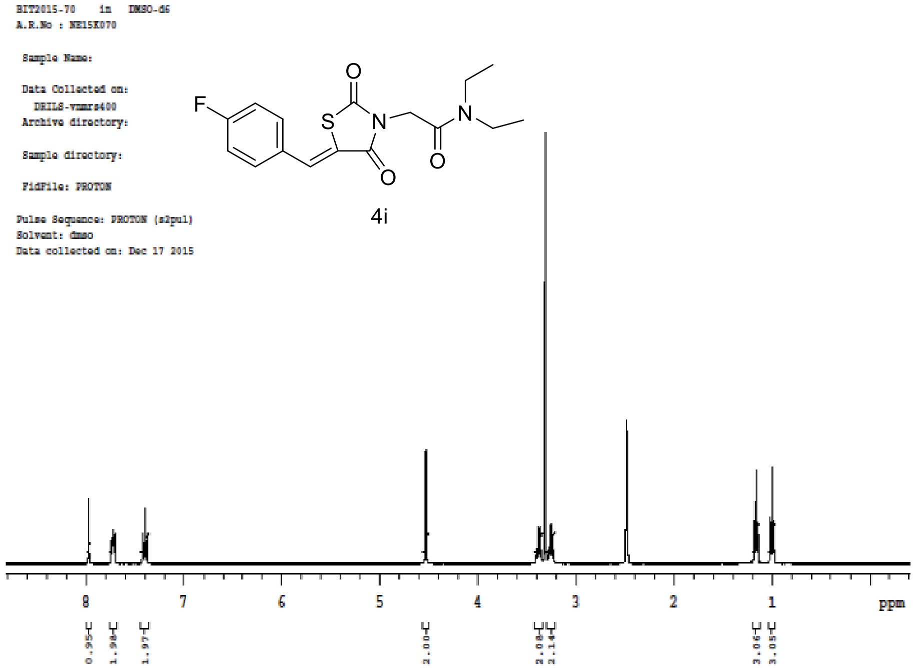

Figure S57. NMR-H spectrum of compound **4i** (DMSO-d_6, 400 MHz)

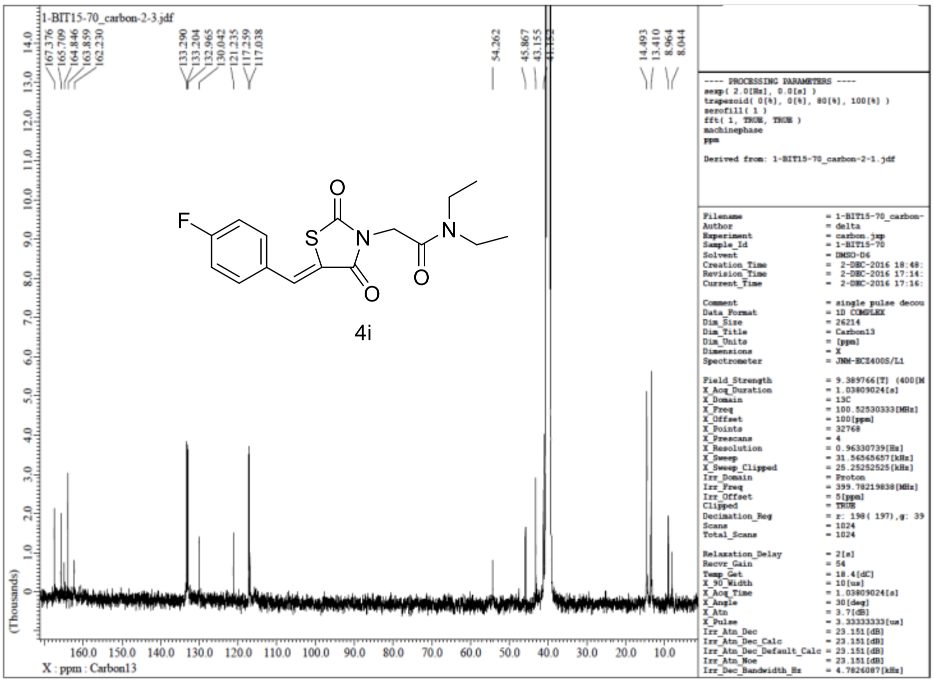

Figure S58. NMR-C[13] spectrum of compound **4i** (DMSO-d_6, 100 MHz)

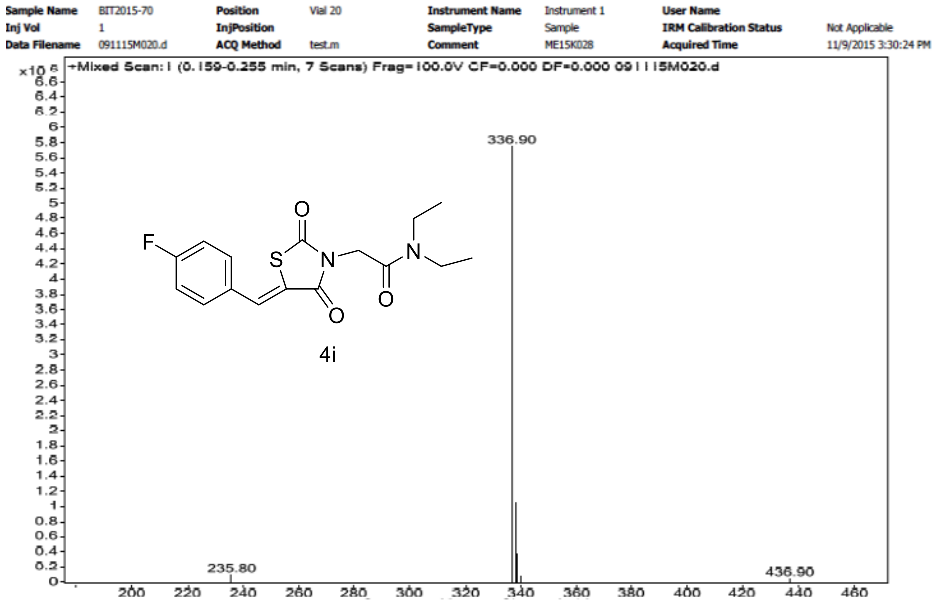

Figure S59. ESI-MS spectrum of compound **4i**

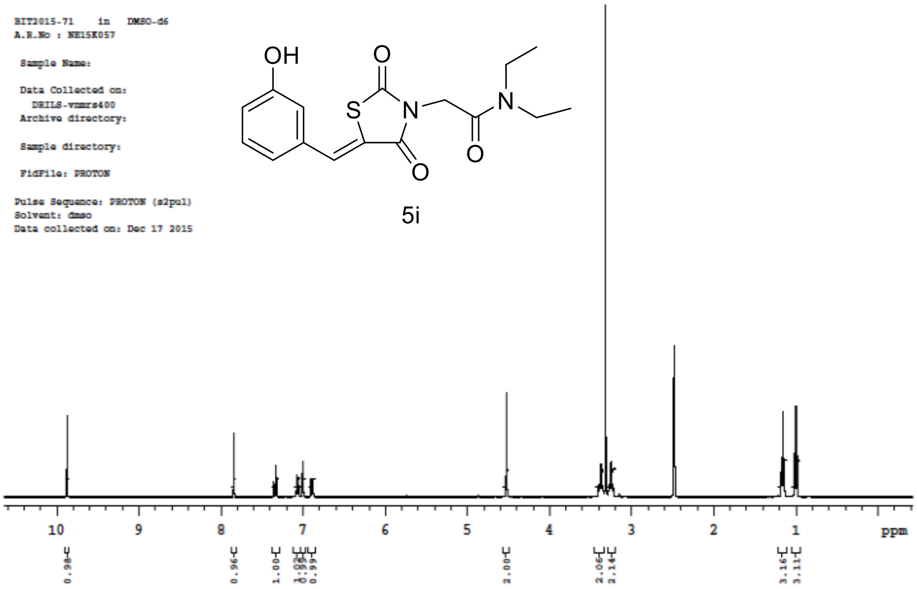

Figure S60. NMR-H spectrum of compound **5i** (DMSO-d_6, 400 MHz)

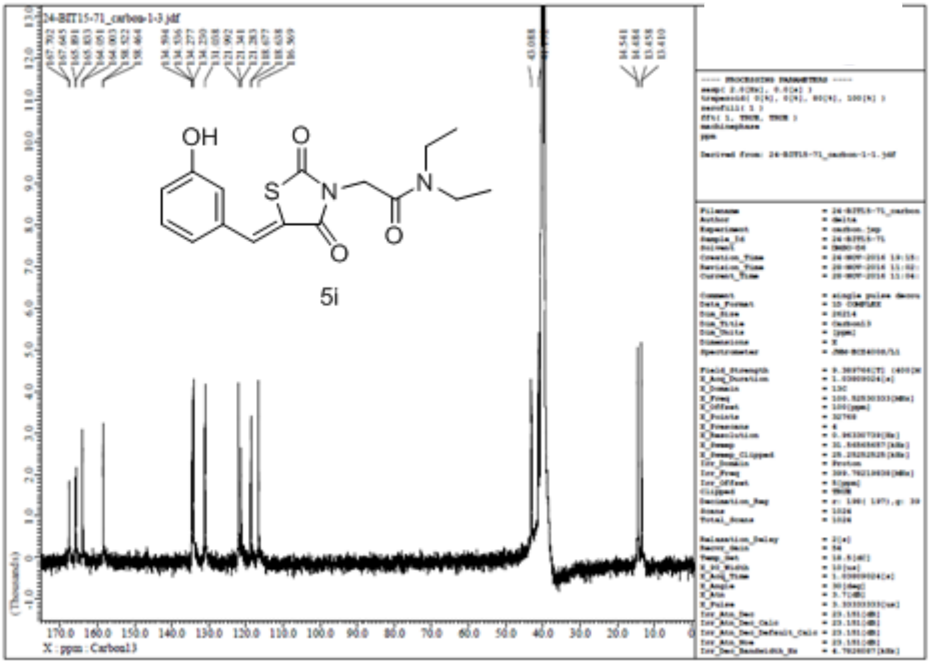

Figure S61. NMR-C^{13} spectrum of compound **5i** (DMSO-d_6, 100 MHz)

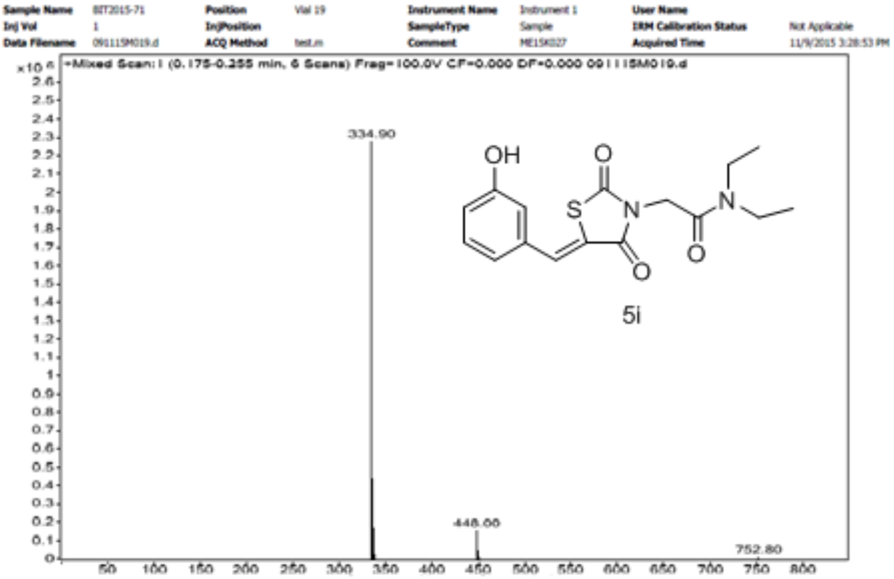

Figure S62. ESI-MS spectrum of compound **5i**

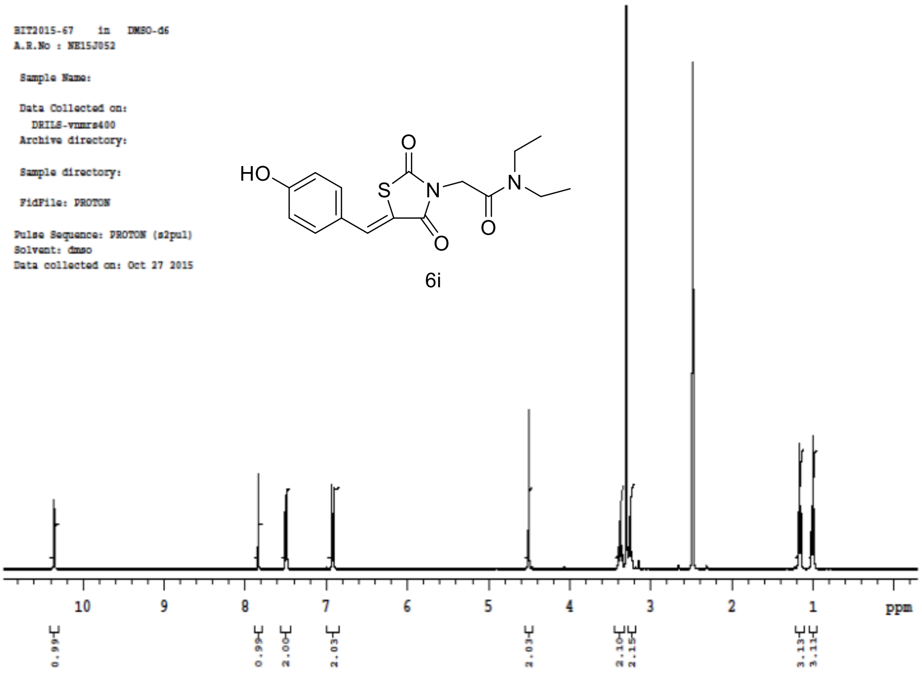

Figure S63. NMR-H spectrum of compound **6i** (DMSO-d_6, 400 MHz)

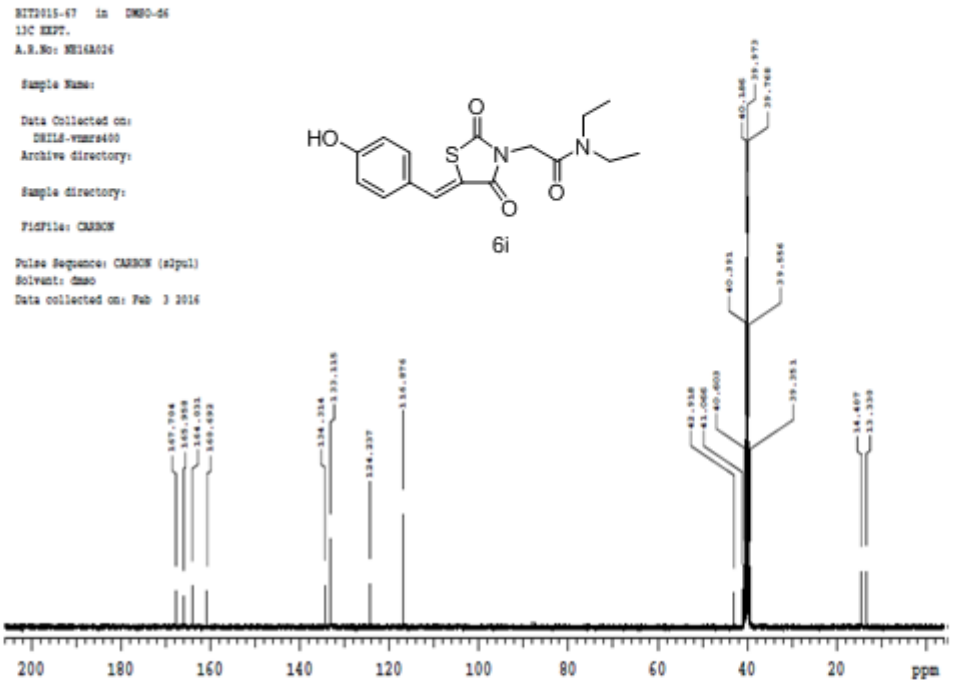

Figure S64.NMR-C[13] spectrum of compound **6i** (100MHz, CDCl3):

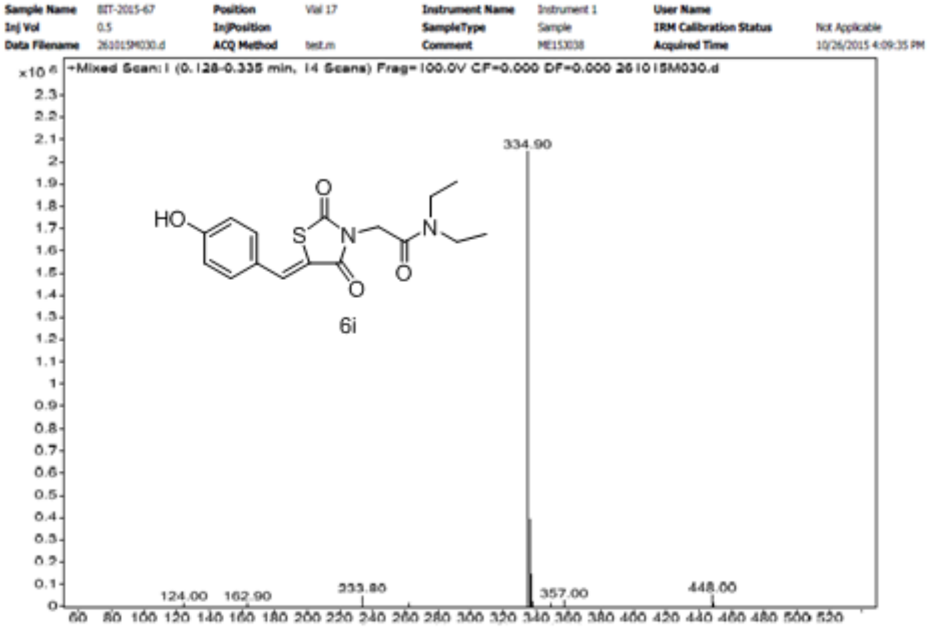

Figure S65. ESI-MS spectrum of compound **6i**

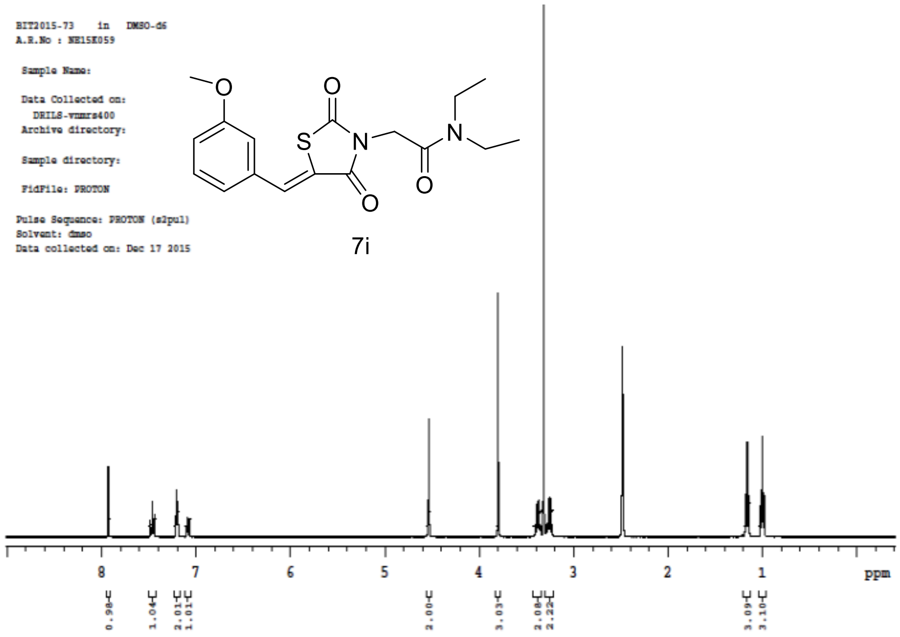

Figure S66. NMR-H spectrum of compound **7i** (DMSO-d_6, 400 MHz)

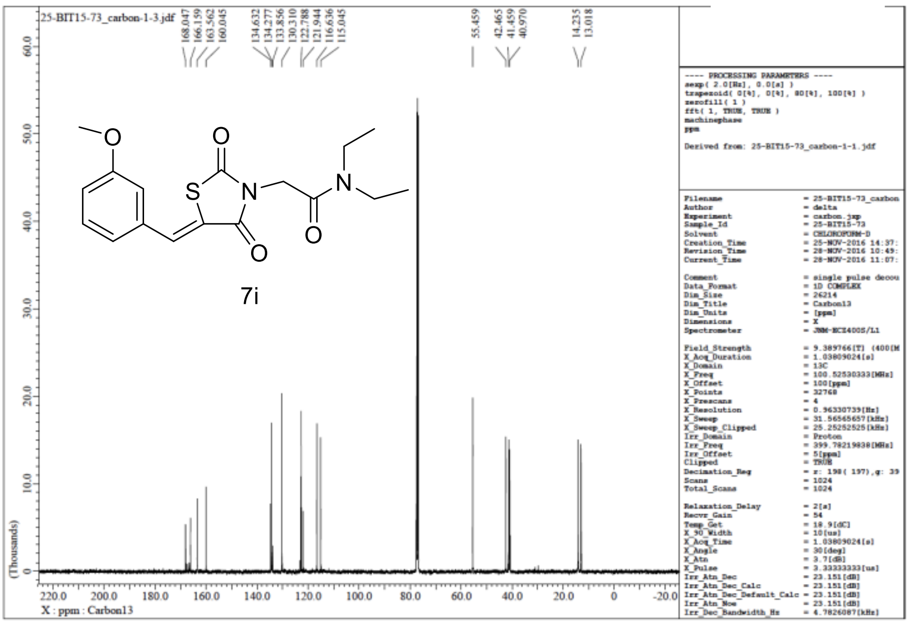

Figure S67. NMR-C[13] spectrum of compound **7i** (100MHz, CDCl3)

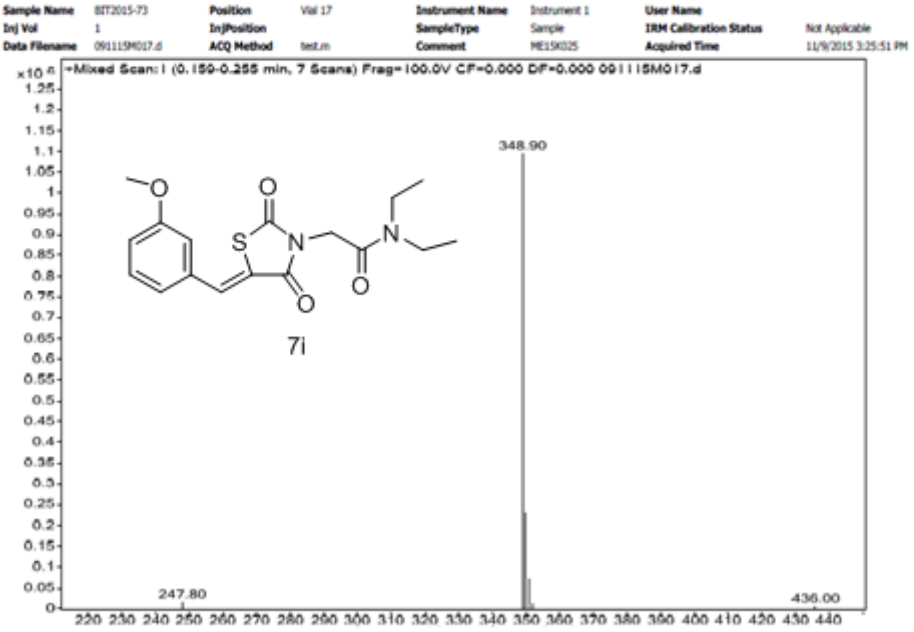

Figure S68. ESI-MS spectrum of compound **7i**

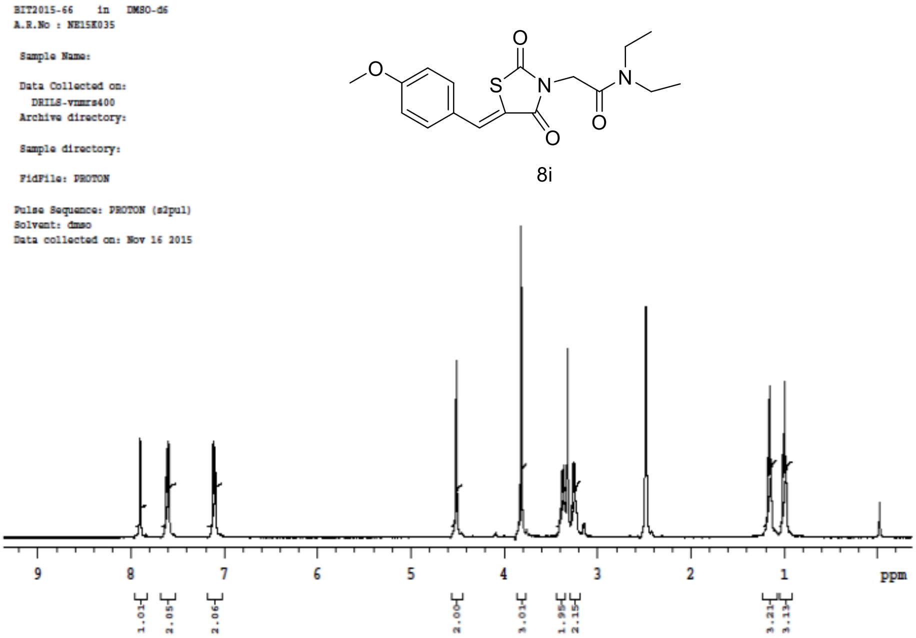

Figure S69. NMR-H spectrum of compound **8i** (DMSO-d_6, 400 MHz)

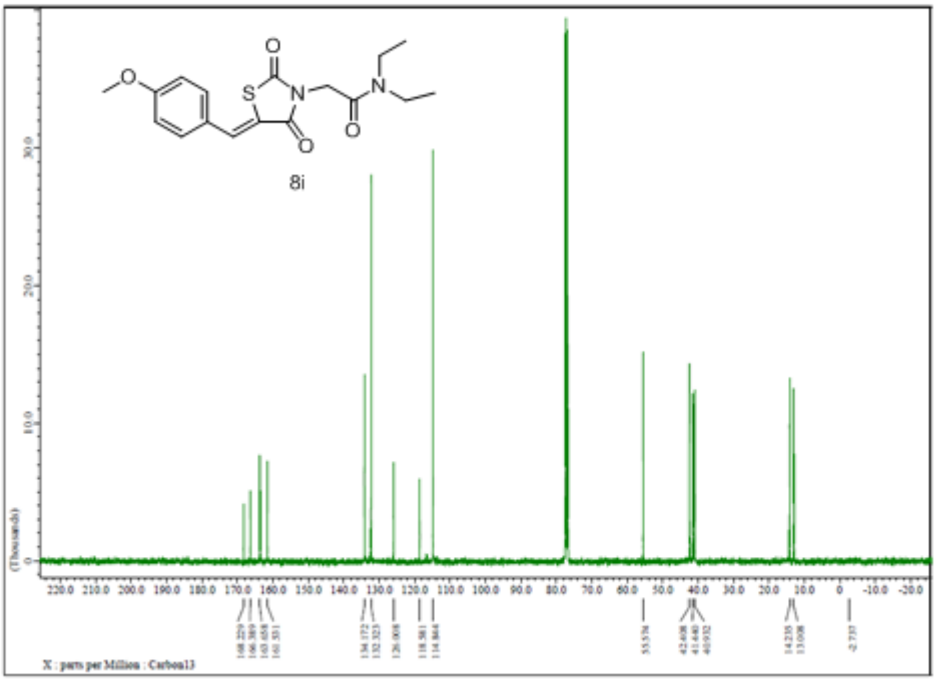

Figure S70. NMR-C^{13} spectrum of compound **8i** (100MHz, $CDCl_3$)

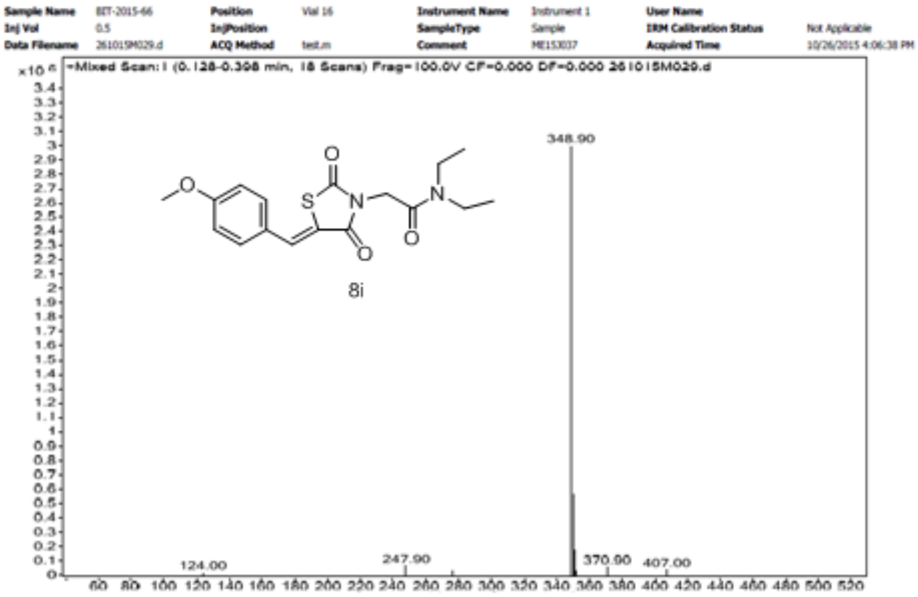

Figure S71. ESI-MS spectrum of compound **8i**

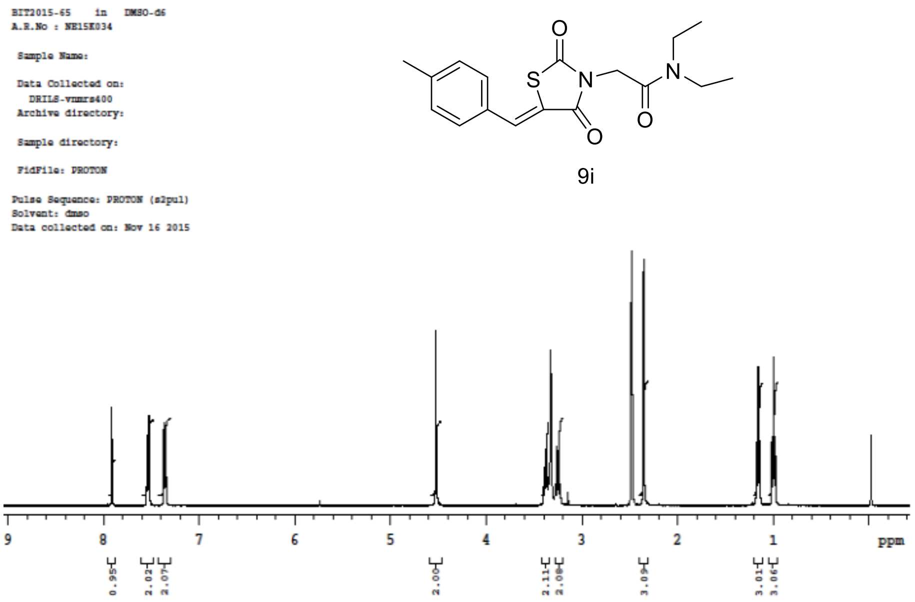

Figure S72. NMR-H spectrum of compound **9i** (DMSO-d_6, 400 MHz)

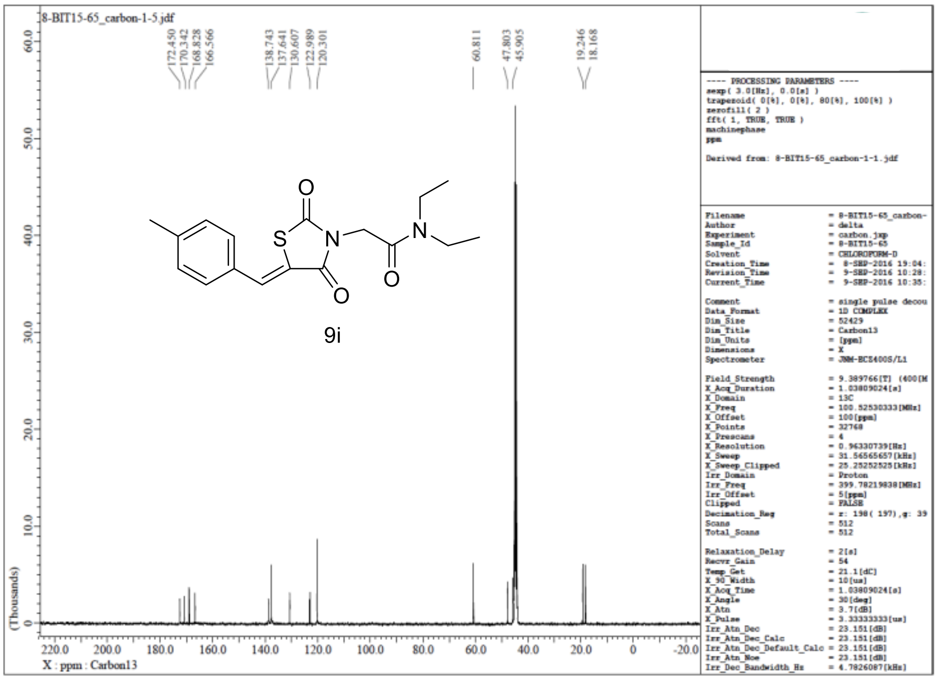

Figure S73. NMR-C[13] spectrum of compound **9i** (100MHz, $CDCl_3$)

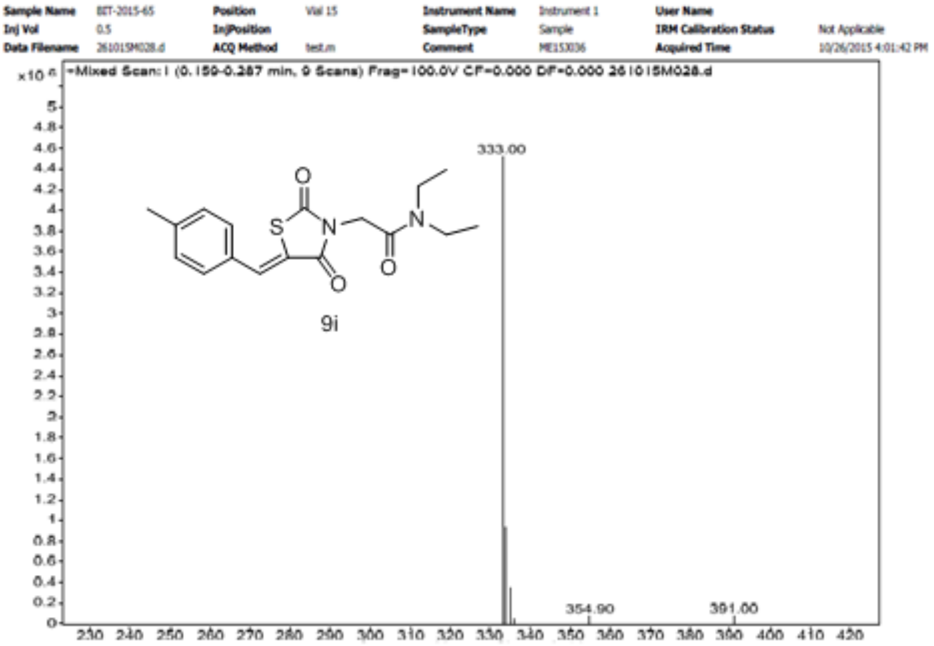

Figure S74. ESI-MS spectrum of compound **9i**

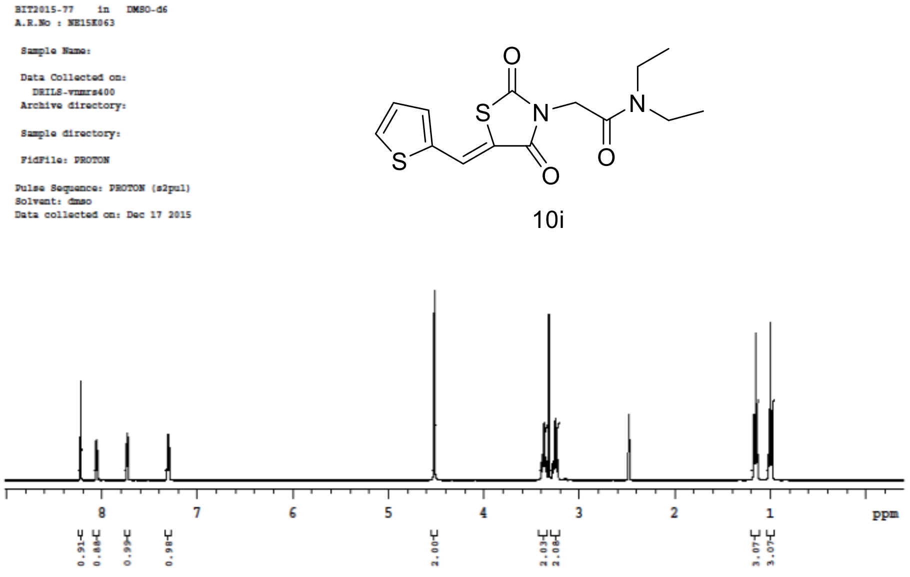

Figure S75. NMR-H spectrum of compound **10i** (DMSO-d_6, 400 MHz)

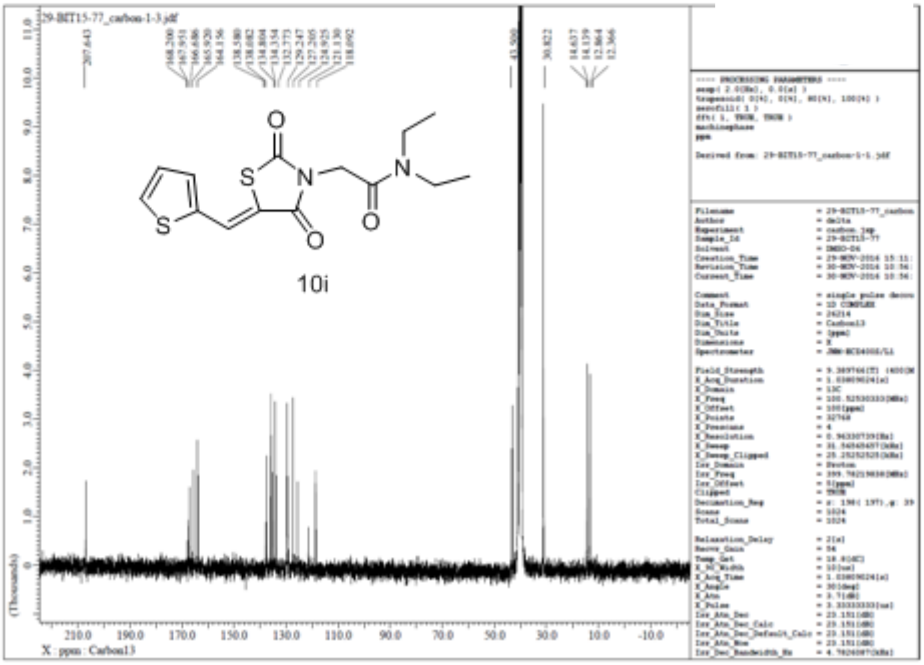

Figure S76. NMR-C[13] spectrum of compound **10i** (DMSO-d_6, 100 MHz)

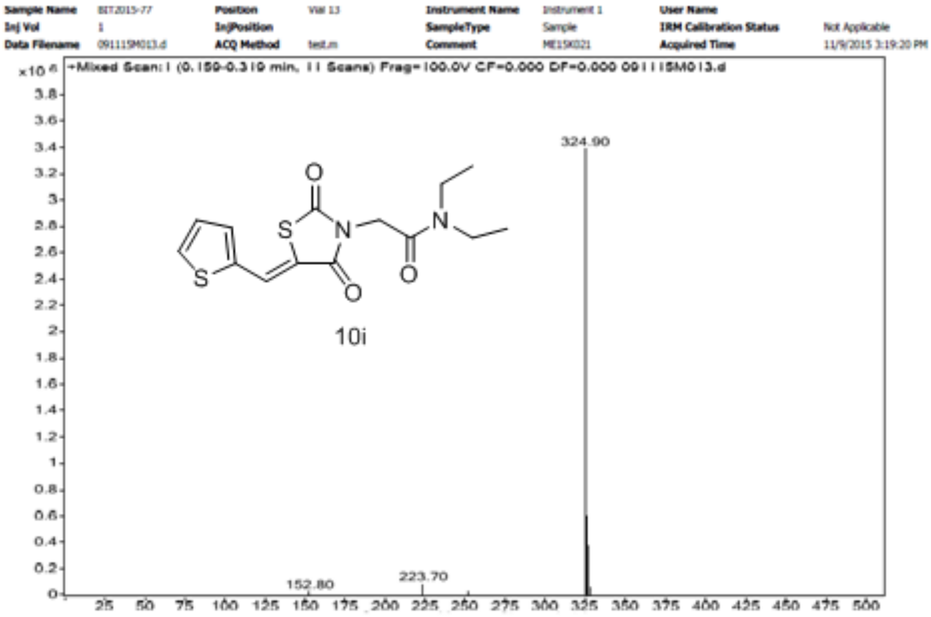

Figure S77. ESI-MS spectrum of compound **10i**

X-ray crystal structure of compound 1i. X-ray diffraction intensity data were collected at room temperature (293K) on a Bruker axs SMART APEXII single crystal X-ray diffractometer equipped with graphite monochromatic MoKα (λ=0.71073 Å) radiation and CCD detector. Crystal dimensions of **1i** are 0.260 × 0.200 × 0.130 mm3. Single Crystal was mounted on a glass fiber using cyanoacrylate adhesive. The unit cell parameters were determined from 36 frames measured (0.5° phi-scan) from three different crystallographic zones using the method of difference vectors. The intensity data were collected with an average four-fold redundancy per reflection and optimum resolution (0.75 Å). The intensity data collection, frames integration, Lorentz and polarization corrections and decay correction were carried out using SAINT-NT (version 7.06a) software[1]. An empirical absorption correction (multi-scan) was performed using the SADABS program[2]. The crystal structure was solved by direct methods using SHELXS-97 and refined by full-matrix least-squares using SHELXL-2014[3] to a final R-value 5.54 %. Molecular geometry was calculated using PARST[4]. All non-hydrogen atoms were refined using anisotropic thermal parameters. The hydrogen atoms were included in the structure factor calculation at idealized positions by using a riding model, but not refined. Images were created with the ORTEP-PLATON program[5]. Refinement: In the **1i** crystals, the hydrogen atoms were placed in calculated positions with C-H = 0.93 Å to 0.97 Å and refined in the riding model with fixed isotropic displacement parameters: $U_{iso}(H) = 1.5U_{eq}(C)$ for the methyl group and $U_{iso}(H) = 1.2U_{eq}(C)$ for the remaining H atoms.

References

1 Schulz, T. *et al.* A comparison of a microfocus X-ray source and a conventional sealed tube for crystal structure determination. *Journal of Applied Crystallography* **42**, 885-891 (2009).

2 Sheldrick, G. M. A short history of SHELX. *Acta Crystallographica Section A: Foundations of Crystallography* **64**, 112-122 (2008).

3 Sheldrick, G. M. Crystal structure refinement with SHELXL. *Acta Crystallographica Section C: Structural Chemistry* **71**, 3-8 (2015).

4 Nardelli, M. PARST95–an update to PARST: a system of Fortran routines for calculating molecular structure parameters from the results of crystal structure analyses. *Journal of Applied Crystallography* **28**, 659-659 (1995).

5 Farrugia, L. J. WinGX and ORTEP for Windows: an update. *Journal of Applied Crystallography* **45**, 849-854 (2012).

Table S1. Crystal data and structure refinement data.

Parameters	Compound 1i
Empirical formula	$C_{16} H_{18} N_2 O_3 S$
Formula weight	318.38
Temperature	293(2) K
Wavelength	0.71073 Å
Crystal system	Monoclinic
Space group	P 2_1/c
Unit cell dimensions	a = 7.1761(4) Å<br>b = 26.1554(11) Å<br>c = 9.0667(4) Å<br>β = 112.127(3) °.
Volume	1576.43(13) A^3
Z, Calculated density	4, 1.341 Mg/m^3
Absorption coefficient	0.219 mm^{-1}
F(000)	672
Crystal size	0.260 x 0.200 x 0.130 mm
Theta range for data collection	1.557 to 28.370 °.
Limiting indices	-9<=h<=9, -34<=k<=31, -12<=l<=11
Reflections collected / unique	15370 / 3929 [R(int) = 0.0382]
Completeness to theta	100.00%
Absorption correction	Semi-empirical from equivalents
Refinement method	Full-matrix least-squares on F^2
Data / restraints / parameters	3929 / 0 / 201
Goodness-of-fit on F^2	1.036
Final R indices [I>2sigma(I)]	R1 = 0.0554, wR2 = 0.1383
R indices (all data)	R1 = 0.0992, wR2 = 0.1616
Largest diff. peak and hole	0.509 and -0.244 e.A^{-3}

Table S2. Hydrogen bond geometry for compound **1i** [Å and °].

D—H…A	D—H(Å)	H…A(Å)	D…A(Å)	D—H…A[°]
C2—H2...S1 (i)	0.93	2.52	3.471(3)	133
C7—H7...O2 (i)	0.93	1.95	2.888(3)	103

Symmetry codes: i) x, y, z.
